# A Stress-Induced Small RNA Modulates Alpha-Rhizobial Cell Cycle Progression

**DOI:** 10.1371/journal.pgen.1005153

**Published:** 2015-04-29

**Authors:** Marta Robledo, Benjamin Frage, Patrick R. Wright, Anke Becker

**Affiliations:** 1 LOEWE Center for Synthetic Microbiology and Faculty of Biology, Philipps-University Marburg, Marburg, Germany; 2 Bioinformatics Group, Department of Computer Science, Albert-Ludwigs-University Freiburg, Freiburg, Germany; University of Geneva Medical School, SWITZERLAND

## Abstract

Mechanisms adjusting replication initiation and cell cycle progression in response to environmental conditions are crucial for microbial survival. Functional characterization of the *trans*-encoded small non-coding RNA (*trans*-sRNA) EcpR1 in the plant-symbiotic alpha-proteobacterium *Sinorhizobium meliloti* revealed a role of this class of riboregulators in modulation of cell cycle regulation. EcpR1 is broadly conserved in at least five families of the Rhizobiales and is predicted to form a stable structure with two defined stem-loop domains. In *S*. *meliloti*, this *trans*-sRNA is encoded downstream of the *divK-pleD* operon. *ecpR1* belongs to the stringent response regulon, and its expression was induced by various stress factors and in stationary phase. Induced EcpR1 overproduction led to cell elongation and increased DNA content, while deletion of *ecpR1* resulted in reduced competitiveness. Computationally predicted EcpR1 targets were enriched with cell cycle-related mRNAs. Post-transcriptional repression of the cell cycle key regulatory genes *gcrA* and *dnaA* mediated by mRNA base-pairing with the strongly conserved loop 1 of EcpR1 was experimentally confirmed by two-plasmid differential gene expression assays and compensatory changes in sRNA and mRNA. Evidence is presented for EcpR1 promoting RNase E-dependent degradation of the *dnaA* mRNA. We propose that EcpR1 contributes to modulation of cell cycle regulation under detrimental conditions.

## Author Summary

Microorganisms frequently encounter adverse conditions unfavorable for cell proliferation. They have evolved diverse mechanisms, including transcriptional control and targeted protein degradation, to adjust cell cycle progression in response to environmental cues. Non-coding RNAs are widespread regulators of various cellular processes in all domains of life. In prokaryotes, *trans*-encoded small non-coding RNAs (*trans*-sRNAs) contribute to a rapid cellular response to changing environments, but so far have not been directly related to cell cycle regulation. Here, we report the first example of a *trans*-sRNA (EcpR1) with two experimentally confirmed targets in the core of cell cycle regulation and demonstrate that in the plant-symbiotic alpha-proteobacterium *Sinorhizobium meliloti* the regulatory mechanism involves base-pairing of this sRNA with the *dnaA* and *gcrA* mRNAs. Most trans-sRNAs are restricted to closely related species, but the stress-induced EcpR1 is broadly conserved in the order of Rhizobiales suggesting an evolutionary advantage conferred by *ecpR1*. It broadens the functional diversity of prokaryotic sRNAs and adds a new regulatory level to the mechanisms that contribute to interlinking stress responses with the cell cycle machinery.

## Introduction

Non-coding RNAs (ncRNAs) have shot to prominence as significant and ubiquitous regulators that are involved in the control of various cellular processes in most eukaryotic and prokaryotic organisms. Although the development of deep-sequencing technologies has allowed for the identification of an ever-growing number of ncRNAs the biological functions and regulatory mechanisms of the vast majority remain veiled. In eukaryotes, short-interfering RNAs (siRNA) and microRNAs (miRNAs) have emerged as a priority research area in biomedicine [1] since they control crucial cellular processes, such as cell development, differentiation and oncogenic transformation [2]. For instance, the miR-34 family mimics p53 activity, inducing cell-cycle arrest and apoptosis [3]. Plant ncRNAs have been reported to regulate stress adaptation and defence responses, but also cell differentiation, such as miR169 that was associated with nodule development in legumes [4,5]. In the fission yeast *Schizosaccharomyces pombe*, meiRNA plays a role in recognition of homologous chromosomes for pairing and thus is essential for progression of meiosis [6,7].

Prokaryotic *tran*s-encoded small RNAs (*trans*-sRNAs) may be considered functional analogs of eukaryotic siRNAs and miRNAs in their ability to post-transcriptionally control gene expression by modulating mRNA translation and stability. The canonical regulatory mechanism of bacterial *trans*-sRNAs involves pairing with a single short binding site within the 5’-untranslated region (UTR) of the target mRNA, which results in formation of an sRNA-mRNA duplex blocking the ribosome binding site (RBS) and/or promoting degradation by RNases [8]. Expression of bacterial sRNAs is commonly stimulated under stress conditions and contributes to the rapid cellular response and adaptation to changing environments. The majority of functionally characterized bacterial sRNAs controls crucial physiological processes like metabolism, transport, chemotaxis, virulence, and quorum sensing [9].

Regulation of DNA replication and cell cycle progression in response to environmental cues is critical to ensure cell survival. Mechanisms involving small molecule-based signaling, protein-protein interactions or regulated proteolysis have been implicated with a delay of replication initiation or septum formation upon facing hostile factors [10]. It is tempting to speculate that *trans*-sRNA-mediated post-transcriptional regulation may also contribute to rapid adaptive stress responses of the cell cycle control circuit in bacteria.

The α-proteobacterium *Caulobacter crescentus* is an important model organism for studying cell cycle regulation. In this bacterium, replication is initiated only once per cell cycle [11,12]. This tight control and exact timing is governed by oscillating concentrations of at least three master regulators, DnaA, GcrA, and CtrA that coordinate the spatio-temporal pattern of phase-specific events ultimately leading to asymmetric cell division [13,14]. DnaA mediates replication initiation and activates *gcrA* expression. GcrA controls components of the replication and segregation machinery and finally induces expression of *ctrA*. CtrA blocks replication initiation by binding to the origin of replication and regulates more than 100 genes. Among these are genes involved in cell division, cell wall metabolism, and motility [15,16]. CtrA activation is driven by the essential CckA-ChpT phosphorelay, which further inactivates CpdR-mediated CtrA proteolysis by phosphorylating this response regulator. When activated by its principal kinase DivJ, DivK silences the CckA-ChpT relay through DivL, allowing for CtrA degradation and replication initiation. Subsequently, DivK is inactivated by dephosphorylation through its primary phosphatase PleC [17].

In the class of α-proteobacteria, several surveys of the non-coding RNome delivered a plethora of *trans*-sRNAs [18–20]. The most comprehensive inventories were performed for members of the *Rhizobiaceae* including *Sinorhizobium meliloti* [21,22]. *S*. *meliloti* exists either in a free-living lifestyle in the soil or in root nodule symbiosis with a leguminous host plant [23,24]. It has emerged as model organism to study adaptation to stress conditions and switching between complex lifestyles. The cell cycle of *C*. *crescentus* and free-living *S*. *meliloti* shows striking similarities that include initiation of replication only once per cell cycle and asymmetric cell division. In spite of species-specific rearrangements of the α-proteobacterial cell cycle regulon, a transcriptional analysis of synchronized *S*. *meliloti* cells has recently identified a conserved core of cell cycle regulated transcripts shared with *C*. *crescentus* [25] and confirmed previous computational comparisons of cell cycle-related genes in α-proteobacteria [26].

Taking advantage of the comprehensive data resource of *trans*-sRNAs in *S*. *meliloti* and related α-proteobacteria, we aimed at identifying riboregulators that post-transcriptionally affect bacterial cell cycle progression. Here, we report on the functional analysis of the stress-induced *trans*-sRNA EcpR1 that is conserved in several members of the Rhizobiales. We present evidence for EcpR1 negatively regulating *dnaA* and *gcrA* at the post-transcriptional level mediated by base-pairing between a strongly conserved loop of this sRNA and the target mRNAs. Our data suggests that EcpR1 contributes to a regulatory network connecting stress adaptation and cell cycle progression.

## Results

### EcpR1 target prediction shows enrichment of cell cycle-related genes

Hypothesizing that riboregulators affecting cell cycle control are more likely to be found among phylogenetically conserved *trans*-sRNAs we performed mRNA target predictions for 27 previously defined RNA families with members in at least two species [27] applying CopraRNA [28]. The predicted targets were screened for an enrichment of cell cycle-related genes. The CopraRNA algorithm considers base pairing strength, hybridization free energy and accessibility of the interaction sites, and integrates phylogenetic information to predict conserved sRNA-mRNA interactions. Many sRNAs base pair at the RBS, however, translation can also be blocked when the pairing region is located 50 or more nucleotides (nt) upstream the RBS or in the open reading frame [29,30]. As suggested by Wright et al. [28], predictions were therefore based on sequences 200 nt upstream and 100 nt downstream of the annotated start codons.

**Fig 1:**
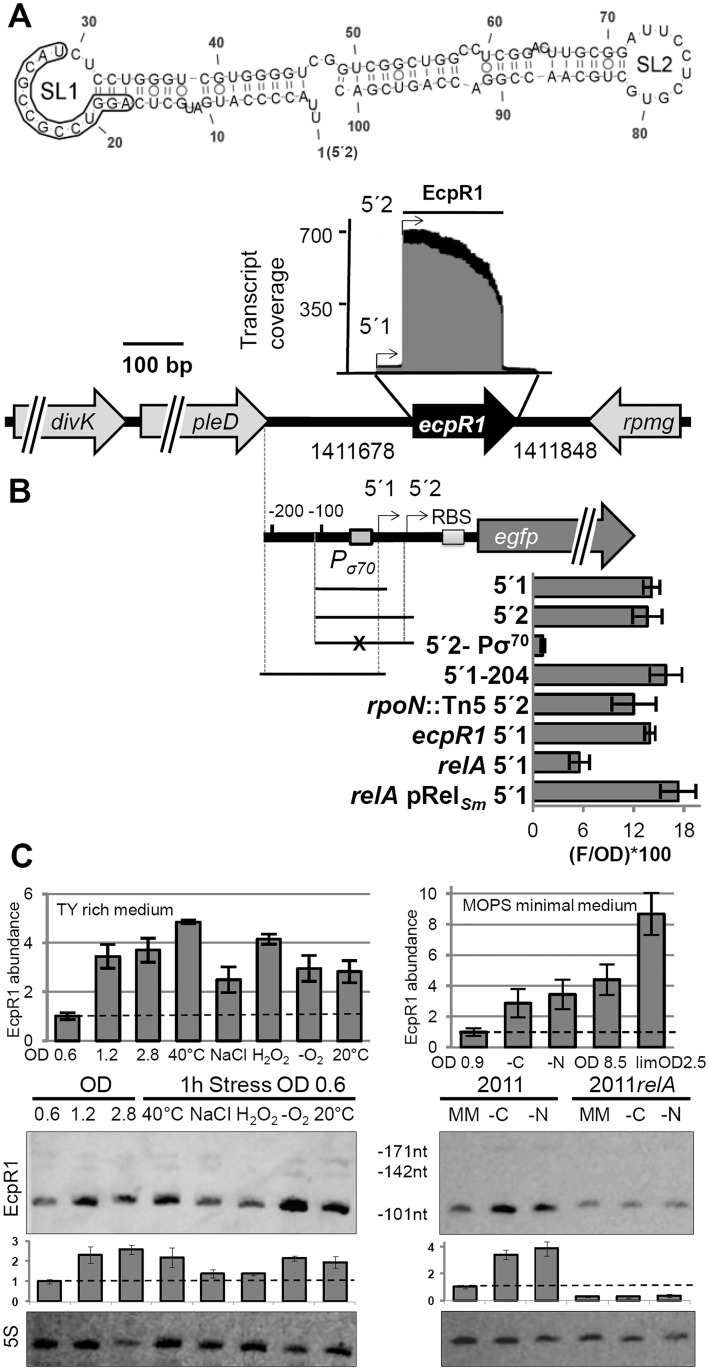
*ecpR1* genomic locus and transcriptional regulation. **(A)** Secondary structure of the dominant EcpR1 101 nt variant with a minimum free energy of -50.20 kcal/mol. Nucleotide positions relative to the second 5’-end are denoted. SL, stem loop domain. The 13 nt region predicted to bind the *gcrA* mRNA is boxed. Below, chromosomal region including the *ecpR1* gene and RNAseq coverage profile of the EcpR1 sRNA in *S*. *meliloti* Rm1021. Genome coordinates of the full length *ecpR1* variant are denoted. Black and grey areas represent coverages from samples enriched for processed and primary transcripts, respectively [21]. Detected EcpR1 5’-ends are depicted by arrows and the dominant 101 nt EcpR1 variant used for structure prediction is marked by the bar. **(B)** Schematic representation of the fragments included in the *ecpR1* transcriptional fusions and fluorescence values of stationary phase Rm2011 wild type and derivative cells harbouring the indicated constructs: 5’1, pP*ecpR1*_5’1; 5’2, pP*ecpR1*_5’2; 5’2-Pσ70, pP*ecpR1*_5’2-Pσ70; 5’1–204, pP*ecpR1*_5’1–204. Specific activities were normalized to OD_600_ to yield fluorescence units per unit of optical density (F/OD). Shown are means and standard deviation values of at least three independent measurements of three transconjugants grown in six independent cultures. **(C)** qRT-PCR analysis and Northern blot detection of EcpR1 transcript abundance in Rm2011 and the *relA* mutant under different growth and stress conditions in TY (left) and MOPS minimal and MOPSlim medium (MM, right). 40°C, heat stress; NaCl, 0.4 mM sodium chloride (osmotic stress); H_2_O_2_, 10mM hydrogen peroxide (oxidative stress); -O_2_, microoxic conditions; 20°C, cold stress; -C and -N, growth in MM until OD_600_ of 0.9 and then MM depleted for 1 hour for carbon or nitrogen. qRT-PCR values were normalized to the SMc01852 transcript and the levels of EcpR1 in Rm2011 growing in TY rich medium at OD_600_ of 0.6 (left) or MOPS minimal medium at OD_600_ of 0.9 (right, dashed line). Plots underneath the Northern blots represent relative hybridization signal intensities. The basal level of EcpR1 in Rm2011 growing in TY rich medium at OD_600_ of 0.6 or MOPS minimal medium at OD_600_ of 0.9 (right) has been normalized to 1 (dashed line) and the sRNA levels in other conditions have been correlated to this value. Mean results from three experiments are shown. Error bars indicate the standard deviation. Exposure times were optimized for each panel.

Targets predicted for the sRNA family established by the *S*. *meliloti trans*-sRNA SmelC291 show a significant enrichment (P-value = 2.5*10^-5^) of cell cycle-related mRNAs (n = 7) among the top-ranked candidates (P≤0.01, n = 89; S1 Table) [27]. The 23 family members are broadly distributed among the Rhizobiales including members in the *Rhizobiaceae*, *Phyllobacteriaceae*, *Xanthobacteriaceae*, *Beijerinckaceae*, and *Hyphomicrobiaceae*. SmelC291, previously named SmrC10 or Sra33, was first identified by comparative genomic predictions of sRNAs [31] and confirmed by RNAseq [21]. In this study we renamed it EcpR1 (elongated cell phenotype RNA1) according to the phenotype induced by its overproduction (see below). In *S*. *meliloti*, *ecpR1* is located in the intergenic region between the *divK-pleD* operon coding for an essential cell cycle response regulator and a diguanylate cyclase, respectively [32] and *rpmG* encoding the 50S ribosomal protein L33 (Fig 1A). In the *Rhizobiacea*, this genomic locus is highly microsyntenic [27]. Northern blot hybridizations confirmed *ecpR1* expression from an independent transcription unit [33] and RNAseq coverage data suggested variants of different length with a dominant 101 nt sRNA [21] which is predicted to form a stable structure with two defined stem-loop domains, SL1 and SL2 (Fig 1A, S1A Fig). SL1 is strongly conserved and positions C_16_ to G_36_ (according to the numbering of EcpR1 nucleotides in Fig 1A) including the loop sequence are identical in all species with EcpR1 homologs analyzed by Reinkensmeier et al. [27]. The 3’-region harbors a putative Rho-independent terminator and 4 terminal U residues (S1A Fig).

In the *Rhizobiaceae*, *gcrA*, *dnaA*, and *pleC* mRNAs appeared among the five top predicted targets (positions 1, 3 and 5, respectively). Furthermore, the two *ftsZ* homologs *(ftsZ1* and *ftsZ2)*, *ctrA* and *minD* encoding a close homolog of the *Escherichia coli* cell division inhibitor [34] were in the top 40 list (P<0.005) of EcpR1 targets (S1 Table). Although there was less agreement with targets predicted in more distantly related members of the Rhizobiales, *gcrA* and *minD* mRNAs were also assessed as highly probable targets when predictions included *Mesorhizobium* strains belonging to the *Phyllobacteriaceae* (P<0.001) or members of the *Xanthobacteriaceae* (P<0.007). Finally, the *pleC* mRNA was still among the top target candidates (P<0.0001) when members of the *Xanthobacteriaceae*, *Beijerinckiaceaceae*, and *Hyphomicrobiaceae* were analyzed.

The GC-rich conserved region within SL1 of EcpR1 was predicted to base pair with all cell cycle-related target mRNA candidates (Fig 1A, S1B Fig). The interacting sequences predicted by CopraRNA were found in different positions of the *S*. *meliloti* mRNAs: for *gcrA*, a 13 nt stretch from position -109 to -95 relative to the start codon (S9D Fig); for *ctrA*, a 8 nt sequence from position -21 to -12 located close to the RBS; and for *pleC* and *minD*, discontinuous base-pairing over a 13 nt stretch overlapping the start codon and the *minC-minD* intergenic region, respectively (S7A–S7C Fig). The putative binding sites in the *dnaA* (S1C Fig; BS5) and *ftsZ* mRNAs (S7D Fig) map to positions about 60 to 70 nt downstream of the start codon. Additionally, the mRNA sequences ranging from the mapped *S*. *meliloti* transcriptional start site (TSS) [22] to 100 nt downstream of the annotated start codon were scanned for further sequences that may interact with EcpR1 applying IntaRNA [35]. This approach suggested three additional putative EcpR1 binding sites in the *dnaA* mRNA with E <-10 kcal/mol (S1C Fig): two at positions -140 and -70 relative to the AUG (BS1 and BS2), and a sequence overlapping the start codon region (BS3). The RNAup webserver [36] also identified these putative EcpR1 binding sites together with a sequence overlapping the RBS (BS4) (S1C Fig).

### *ecpR1* is expressed upon entry into stationary phase and under stress conditions

Microarray-based transcriptome profiling detected EcpR1 upon heat, cold, acidic, alkaline, salt, and oxidative stresses [21,37]. In the *S*. *meliloti* Rm2011 wild type, Northern blots revealed a dominant ~100 nt EcpR1 transcript and two less abundant larger variants corresponding to the prevalent 101 nt species, a 142 nt transcript, and the full length 171 nt variant deduced from the RNAseq data (Fig 1A and 1C; S1A Fig). In TY rich medium EcpR1 was barely detected in exponentially growing bacteria (OD_600_ of 0.2 to 0.9), and levels increased during early and late stationary phases (OD_600_ of 1.2 to 2.8) (Fig 1C, S2A Fig). The amount of EcpR1 also increased after shifting exponential phase cultures for one hour to 40°C, 20°C, or microoxic conditions, and after adding salt or hydrogen peroxide (~1.5 to 2 fold induction) (Fig 1C). qRT-PCR quantification of EcpR1 transcripts including the sequence region of the 101 nt variant even suggested higher induction levels (up to ~5-fold upon temperature upshift) (Fig 1C). EcpR1 levels also increased when exponential phase cells growing in MOPS minimal medium were shifted to carbon or nitrogen depleted medium for one hour (~3.5-fold induction) (Fig 1C). Higher induction rates were observed in MOPS and nutrient-limited MOPS (MOPSlim) stationary phase cultures (up to ~8.5-fold, Fig 1C). Under these conditions, the stationary phase was reached at OD_600_ of 8.5 and 2.5, respectively. EcpR1 was not detected in total RNA isolated from 28 days old mature symbiotic nodules of *Medicago sativa* (S2B Fig).

RNAseq identified two distinct 5’-ends of the *ecpR1* mRNA varying by 29 nt [22] (Fig 1A). Although these 5’-ends were associated to σ^70^- (ATTGAT-N17-CAATGC) (Fig 1B) and σ^54^-type (AGGAAGG-AAAC-TTCCA) promoter motifs (S2C Fig), the alternative 5’2-end may either be generated by the activity of the putative σ^54^-dependent promoter or by post-transcriptional processing of the EcpR1 primary transcript. To determine promoter activities associated with *ecpR1*, different DNA fragments from the *ecpR1* upstream region including up to 12 nt downstream of the TSS were fused to *egfp* in a replicative low copy plasmid (Fig 1B). Matching the results from the Northern hybridizations and qRT-PCR, the pP*ecpR1*_5’2 construct showed very low activities, just surpassing background fluorescence in the exponential growth phase of Rm2011 cultures in TY rich medium, while in stationary phase activities strongly increased (S2D Fig). Microscopy of Rm2011 single cells carrying pP*ecpR1*_5’2 showed that overall fluorescence homogeneously increased in stationary phase (S2E Fig), further confirming the growth phase-dependent pattern of *ecpR1* expression. All constructs including the σ^70^ promoter motif (pP*ecpR1*_5’1, pP*ecpR1*_5’2, and pP*ecpR1*_5’1–204) showed similar activities in stationary phase (Fig 1B). Mutations in the -10 region of the σ^70^-type promoter abolished fluorescence activity of the reporter plasmid pP*ecpR1*_5’2-Pσ^70^ (Fig 1B) and EcpR1 was not detected by Northern hybridizations in stationary growing and oxygen depleted 2011Pσ^70^ *ecpR1* bacteria carrying these promoter mutations in the genome (S2F Fig). Furthermore, in stationary cultures an *rpoN* mutation did not reduce the reporter gene activity mediated by the pP*ecpR1*_5’2 construct including both putative promoters (Fig 1B). This suggests that the predicted σ^54^-type promoter is non-functional under the conditions tested and implies that the prominent 5’-end of EcpR1 was probably generated by ribonucleolytic activity. *ecpR1* was not required for stimulation of *ecpR1* promoter activity in the stationary phase excluding a positive feedback involving the EcpR1 sRNA (Fig 1B). In trans overproduction of PleD or DivK, encoded upstream of *ecpR1* (Fig 1A), did not affect activity of any of the reporter gene constructs (S2G Fig).

Since the predicted promoter motifs provide no hints to extracytoplasmic function sigma factors being involved in stress-induced stimulation of *ecpR1* expression, we assayed the role of the stringent response alarmone ppGpp in regulation of *ecpR1*. Previously reported transcriptome data of cultures shifted to nitrogen or carbon starvation indicated a 20-fold and 4-fold increase in EcpR1 levels in the wild type and *relA* mutant, respectively [38]. Compared to the wild type, stimulation of *ecpR1* expression was reduced more than two-fold in a *relA* mutant that is unable to synthesize ppGpp and was fully restored by ectopic *relA* expression driven by the basal activity of the non-induced *lac* promoter [38] (Fig 1B). This result is in agreement with comparable levels of EcpR1 in the *relA* mutant under nutrient-sufficient and nitrogen- or carbon-limiting conditions as inferred from Northern hybridizations (Fig 1C) suggesting that EcpR1 is part of the stringent response regulon in *S*. *meliloti*.

### Overexpression of *ecpR1* leads to cell cycle defects in several related α-proteobacteria

To study the biological function of EcpR1, growth and morphology phenotypes were monitored in *S*. *meliloti* either overexpressing *ecpR1* or lacking a functional copy of this sRNA gene.

**Fig 2:**
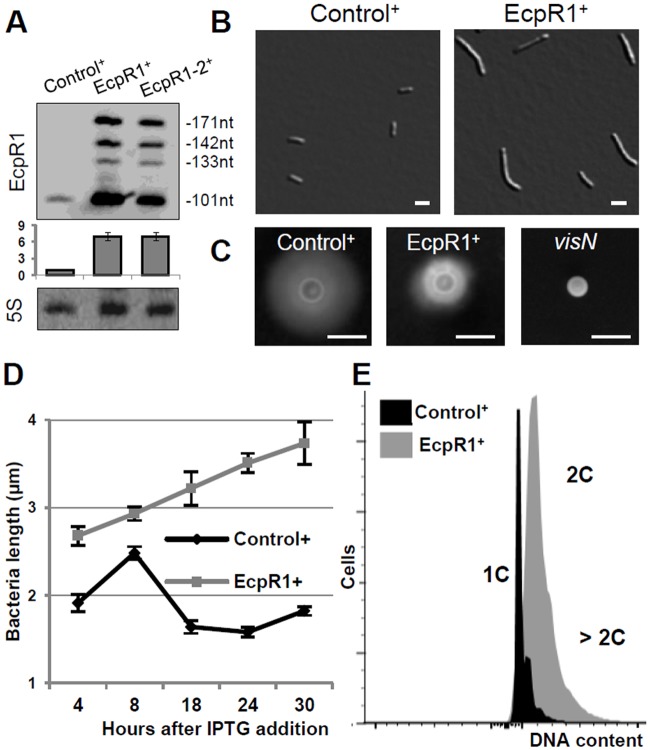
Elongated cell phenotype induced by *ecpR1* overexpression. **(A)** Northern blot detection of EcpR1 RNA variants in Rm4011 strains carrying either pSKControl^+^ (Control^+^), pSKEcpR1^+^ (EcpR1^+^), or pSKEcpR1-2^+^ (EcpR1-2^+^) 4 hours after induction with IPTG. Below, relative hybridization signals derived from the 101 nt EcpR1 species are plotted. The wild type level of EcpR1 in Control^+^ cells (OD_600_ of ~0.9) has been normalized to 1 (dashed line) and the sRNA levels in other conditions are correlated to that value. Mean results from three experiments are shown. Error bars indicate the standard deviation. **(B)** Cell morphology, **(C)** motility assay, **(D)** cell length, and **(E)** DNA content of *S*. *meliloti* strains overexpressing *ecpR1* or the SmelC812 control antisense RNA gene. The 2011*visN* mutant was used as negative control for swimming motility. 1C and 2C indicate one and two genome equivalents, respectively. Bars correspond to 2 μm in *B* and 5 mm in *C*. Error bars in *D* represent standard errors (n = 100 cells).

IPTG-induced overexpression of *ecpR1* was mediated by construct pSKEcpR1^+^ in strain Rm4011 carrying mutations that prevent background activity of the applied inducible expression system (see materials and methods). Northern hybridizations verified IPTG-driven overexpression of *ecpR1* from plasmid pSKEcpR1^+^. Due to the overall stronger signals, the three less abundant EcpR1 variants matching the RNAseq data [21] were clearly detected in addition to the dominant 101 nt EcpR1 transcript (Figs 1A and 2A; S1A Fig, S2A Fig). IPTG-driven overexpression of the SmelC812 RNA gene from plasmid pSKControl^+^ served as control in all *ecpR1* overexpression assays because it did not affect the overall integrity of the cell, as growth phenotype and transcriptome profiles did not significantly deviate from the wild type properties. SmelC812, an antisense RNA of insertion sequence ISRm19, was postulated to prevent translation of its associated TRm19 transposase mRNA [21].

Induced overexpression of *ecpR1* led to abnormal cell elongation (Fig 2B). The mean cell length progressively increased after exposure to IPTG (Fig 2D). 30 hours post-induction 90% of the *ecpR1* overexpressing cells were abnormally long and 3% of the population additionally showed a branched morphology (sampling of 1000 cells). Similar abnormal cell morphologies have previously been reported in response to a variety of cell cycle perturbations that inhibit or overstimulate either DNA replication or cell division [32,34,39–41]. *ecpR1* overexpressing cells showed a ~2-fold decrease in generation time (~4 hours) compared to those overproducing the control sRNA (~2 hours), measured as the average time between two cell divisions monitored by time-lapse microscopy on TY rich medium (S3A Fig). Time-lapse microscopy also showed that after 30 hours of growth in presence of IPTG 38% of the elongated cells (n = 500) were not able to proceed to cell division and to resume growth after transfer to fresh medium lacking the inductor, compared to 4% of equally treated pSKControl^+^ cells (S3B Fig). Furthermore, after three cycles of regrowing EcpR1 overproducing cultures on TY rich medium supplemented with IPTG, a 64% decrease in viable cells was observed (S3C Fig). Cells overproducing EcpR1 spread to a smaller halo (diameter 8 ± 2 mm) than the control (16 ± 1 mm) on soft agar (Fig 2C), but were still motile compared to a *visN* mutant incapable of swimming [42]. Finally, we checked alterations of the DNA content by fluorescence-activated cell sorting (FACS) analysis. 4 hours post-induction, cells with two genome copies started to accumulate in comparison to the control, and after 20 hours the majority of cells contained 2 or more genome equivalents (Fig 2E), further suggesting perturbations of the cell cycle.

Because homologs of EcpR1 and cell cycle-related target candidates were also found in other members of the Rhizobiales, we asked whether overproduction of *S*. *meliloti* EcpR1 also leads to cell cycle defects in related species. This phenotype was conserved in the genera *Sinorhizobium* and *Rhizobium*, as IPTG-induced overexpression of *ecpR1* in *S*. *medicae*, *S*. *fredii*, *R*. *tropicii*, and *R*. *radiobacter* carrying plasmid pSKEcpR1^+^ led to a similar proportion of elongated and branched cells as observed in *S*. *meliloti* (S4A Fig). In *R*. *etli* and *A*. *tumefaciens*, cell cycle associated defects were less abundant but FACS analysis confirmed an increased proportion of cells with more than two genome copies (S4B Fig).

### Deletion of *ecpR1* attenuates competitiveness

The markerless 2011*ecpR1* mutant, missing the sequence of the full length 171 nt *ecpR1* variant, did not show distinct phenotypes in that it grew similarly to the wild type, even under the stress conditions which stimulated *ecpR1* expression (S5A–S5D Fig). After growth in rich medium or defined nutrient-limited minimal media until late stationary phase or after application of stress conditions growth recovery and cell viability (CFU/ml) were also not significantly affected compared to the wild type. Furthermore, the *ecpR1* deletion mutant was not impaired in symbiosis with its host plant *M*. *sativa* (S5E–S5H Fig).

**Fig 3:**
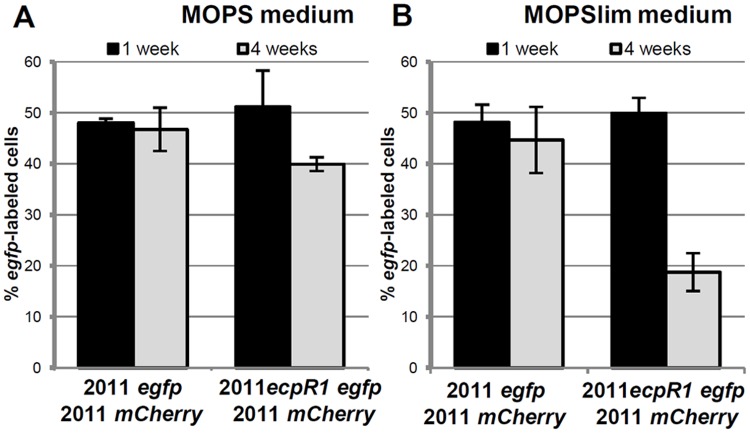
Lack of *ecpR1* reduces competitiveness of Rm2011. Mean percentage of *egfp*-labeled cells 1 and 4 weeks after mixing 2011*mCherry* with either 2011*egfp* or 2011*ecpR1 egfp* cells at a 1:1 ratio in MOPS (A) or MOPSlim media (B). Every week the mixed population was diluted 1000-fold in fresh media. The percentage of *egfp*-labeled cells was determined by microscopy. Error bars indicate the standard deviation of 3 biological replicates.

The strong conservation and microsynteny suggests an evolutionary advantage conferred by the *ecpR1* locus. To support this hypothesis we determined whether the Rm2011 wild type has a fitness advantage over the *ecpR1* mutant. For this competitive growth assay, strains were labeled by a stable genomic integration of plasmids carrying either *egfp* or *mcherry* driven by a constitutive promoter. MOPS or Nutrient-limiting MOPS (MOPSlim) minimal media were inoculated with 2011 *mCherry* cells and either 2011 *egfp* or 2011*ecpR1 egfp* cells in a ratio of 1:1. eGFP:mCherry fluorescence ratios of the mixed cultures were measured and microscopy images were taken to determine the percentage of *egfp*-labeled bacteria (Fig 3, S6 Fig). After 7 days of cultivation, the 1:1 ratio was maintained indicating that all strains grew similarly, as we have previously observed when single-strain liquid cultures were grown in these conditions (S5 Fig). However, after the 7 days-old mixed cultures were diluted in fresh media, the proportion of the 2011*ecpR1 egfp* strain progressively decreased in the MOPSlim medium (S6C and S6D Fig). The mixture of 2011*egfp* and 2011*mCherry* cultures further on maintained the ~1:1 ratio, confirming that the fluorescence markers are neutral in the conditions tested (Fig 3). After three consecutive sub-cultivations, the *ecpR1* mutant only reached ~40% and ~20% of the population in MOPS and MOPSlim media, respectively (Fig 3). This implies a disadvantage of the *ecpR1* deletion mutant in recovery from late stationary cultures as compared to the wild type, particularly under nutrient limitation.

### *ecpR1* overexpression or deletion alters expression of genes related to cell cycle regulation

To obtain further clues to putative target genes of EcpR1 the cellular responses of the *S*. *meliloti* EcpR1 overproducing strain and the *ecpR1* deletion mutant (2011*ecpR1*) were characterized by microarray-based transcriptome profiling.

**Fig 4:**
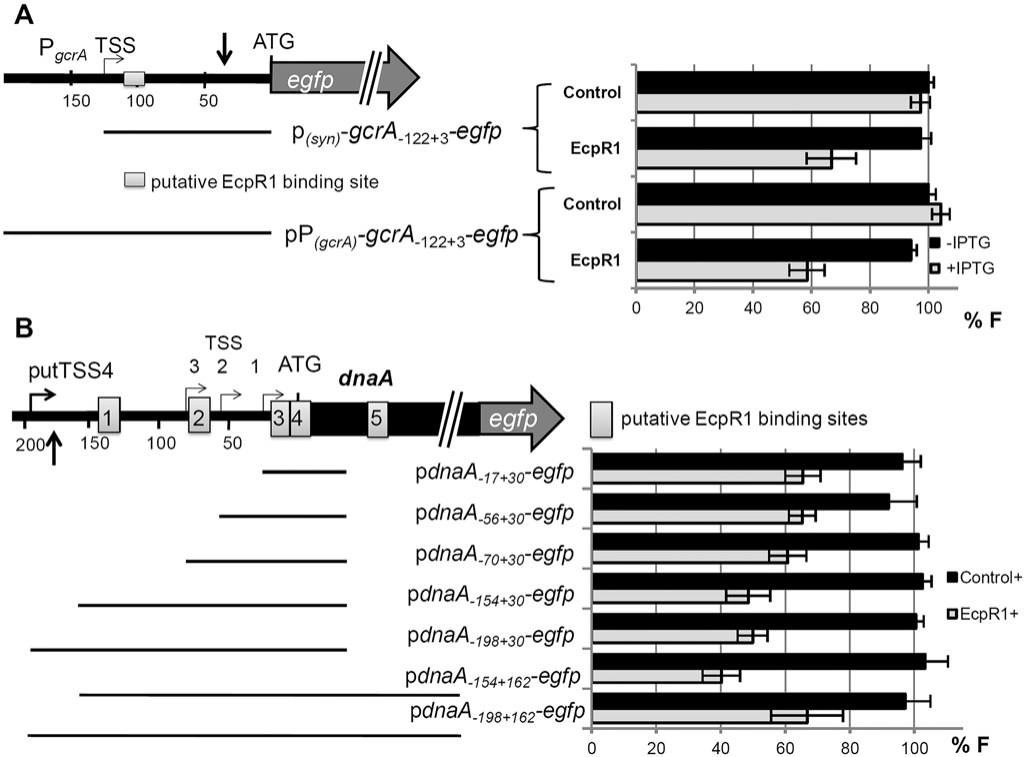
EcpR1 post-transcriptionally represses *gcrA* (A) and *dnaA* (B). Schematic representations of the genomic regions and the fragments (indicated by bars) translationally fused to *egfp*. Positions are denoted relative to the AUG; A is +1. Grey boxes indicate potential EcpR1-binding sites. Vertical arrows mark the regions covered by the oligonucleotide probes displaying altered signal intensities in the microarray hybridizations after *ecpR1* overexpression (see details in text). Means of relative fluorescence intensity values of Rm4011*ecpR1* co-transformed with the *ecpR1* or control SmelC812 overexpression plasmid, and the indicated reporter plasmid are shown below. The standard deviation represents at least three independent determinations of three double transconjugants grown in six independent cultures. Specific activities were normalized to the levels of the strain carrying the vector with the control RNA gene without IPTG added to yield percent relative fluorescence (% F).

Differential gene expression upon EcpR1 overproduction: Genes displaying differential expression 15 minutes, 1 hour, and 4 hours post-induction of *ecpR1* overexpression in TY medium are listed in S2–S6 Tables. Only reporter oligonucleotides associated to the open reading frame or UTRs of 6 (15 minutes post-induction) and 20 (1 hour post-induction) protein-coding genes indicated transcript levels at least 1.6-fold lower than in the control. No genes were found to be upregulated after 15 minutes (except for *ecpR1* that was overexpressed) whereas RNA levels associated to 35 coding regions or UTRs including a number of ribosomal genes were upregulated after 1 hour. 4 hours post-induction, which corresponds to completion of one cell cycle in EcpR1 overproducing cells, transcript levels of 77 protein-coding genes were found to be changed (25 increased and 51 decreased). Several downregulated genes were related to cell cycle regulation and motility, which is in accordance with the observed phenotypes (Fig 2B–2E). Among these were *divJ* as well as the SMc00887-SMc00888 operon of unknown function that shares similarities with the *pleD-divK* operon located upstream of the *ecpR1* gene (Table 1). Previously, a decrease in SMc00887 and SMc00888 transcript levels was also found to be caused by mutation of *podJ* encoding a polarity factor [43]. The putative cell cycle-related SMc00888 gene was among the predicted EcpR1 targets (Table 1, position 22). Our transcriptome study also indicated lower representation of the *gcrA* 5’-UTR and increased levels of the long putative *dnaA* 5’-UTR region upstream of the predicted EcpR1 binding sites (Table 1 and Fig 4, vertical arrows), both among the top three ranked candidates of the computational EcpR1 target predictions (S1 Table). Most of the genes strongly differentially expressed upon EcpR1 overproduction are related to metabolism. We also found reduced levels of the 5’-UTR sequence of the ribonuclease gene *rne* 1 hour (M = -0.77) and 4 hours (M = -1.78) after *ecpR1* overexpression. Moreover, the 5’-UTR sequence of *xerC* (M = +2.40), probably involved in chromosome segregation, and *mepA* (M = +1.15) encoding a homolog of peptidoglycan hydrolases, stood out among the upregulated transcripts 4 hours post-induction. qRT-PCR confirmed the observed changes in transcript levels of *dnaA*, *gcrA*, *divJ*, and SMc00888 in response to EcpR1 overproduction. Although not detected as differentially expressed in the microarray hybridizations, qRT-PCR showed reduced levels of the *ctrA*, *ftsZ1*, *pleC*, and *minD* transcripts in EcpR1 overproducing cells (Table 1).

**Table 1:** qRT-PCR based verification of putative EcpR1 target genes displaying changes in transcript levels upon overproduction of EcpR1 as detected by global transcriptome profiling.

	Ratio of transcript levels: EcpR1 vs. SmelC812 overproduction		
Gene	Description*	Log_2_ ratio (qRT-PCR)	M value (microarray)
5’-UTR *gcrA* (-61 to -20)	cell cycle regulator GcrA	-1.03 ± 0.04	-0.76 ± 0.37
*gcrA*	cell cycle regulator GcrA	-2.06 ± 0.10	-0.41 ± 0.27
5’-UTR *dnaA*_5561 (-372 to -319)	chromosomal replication initiator DnaA	+1.23 ± 0.07	1.42 ± 0.40
5’-UTR *dnaA*_5562 (-222 to -174)	chromosomal replication initiator DnaA	+1.06 ± 0.04	0.74 ± 0.41
*dnaA*	chromosomal replication initiator DnaA	-1.54± 0.05	-
*ctrA*	cell cycle transcriptional regulator CtrA	-1.29 ± 0.04	-0.48 ± 0.32
*divJ*	sensor histidine kinase DivJ	-1.76 ± 0.10	-0.71 ±0.39
5’-UTR SMc00888 (-236 to -188)	2-component receiver domain protein SMc00888	-4.83 ± 0.17	-2.15 ± 0.63
SMc00888	2-component receiver domain protein SMc00888	-5.11 ± 0.24	-0.99 ±0.34
*ftsZ1*	cell division protein FtsZ1	-1.33 ± 0.05	-0.46 ± 0.42
*pleC*	sensor histidine kinase PleC, DivK phosphatase	-2.25 ± 0.07	-
*minD*	putative cell division inhibitor MinD	-0.46 ± 0.01	-

Log_2_ change in transcript amount normalized to levels of the SMc01852 mRNA. Errors represent the standard deviation of three replicates. Positions of microarray reporter oligonucleotides relative to the start codon are given in brackets for 5’-UTR regions.

*Description of gene product or associated gene product.

Differential gene expression in the *ecpR1* deletion mutant compared to the wild type: The transcriptomes of Rm2011 and Rm2011*ecpR1* cells were compared during stationary growth in MOPS and MOPSlim media (S7–S10 Tables) since *ecpR1* expression is stimulated in the wild type under these conditions (Fig 1C, right panel). Reporter oligonucleotides associated to the open reading frame or UTRs of 18 (MOPS medium) and 17 (MOPSlims medium) protein-coding genes indicated transcript levels at least 1.6-fold lower than in the wild type control. Among them were reporters for the *dnaA* 5’-UTR region (positions -158 to -121 in MOPS and -222 to -174 in MOPSlim media), and *mepA*, both upregulated 4 hours after *ecpR1* overexpression.

In contrast, transcript levels of 27 (MOPS medium) and 44 (MOPSlims medium) protein-coding genes were found to be upregulated. Under both conditions *pleC*, ranking in the 5^th^ position of the computationally predicted EcpR1 targets (S1 Table), displayed significantly higher transcript levels and was downregulated in EcpR1 overproducing cells (Table 1). Although *gcrA*, *dnaA*, and *pleC* microarray reporter signals did not pass all criteria set for the identification of differentially expressed genes, qRT-PCR indicated higher transcript levels of these genes in 2011*ecpR1* compared to the wild type (Table 2). This is in agreement with downregulation of these cell cycle-related genes upon *ecpR1* overexpression (Table 1).

**Table 2:** qRT-PCR based verification of putative EcpR1 target genes displaying expression changes in 2011*ecpR1* vs. Rm2011 wild type growing in MOPS or MOPSlim media.

		Ratio of transcript levels: EcpR1 vs. SmelC812 overproduction	
Gene	Description*	Log_2_ ratio (qRT-PCR)	M value (microarray)
5’-UTR *gcrA* (-61 to -20)	cell cycle regulator GcrA	0.81 ± 0.07 (MOPS)	-
		0.62 ± 0.06 (MOPSlim)	0.50 ± 0.28 (MOPSlim)
*gcrA*	cell cycle regulator GcrA	0.70 ± 0.10 (MOPS)	-
		1.31 ± 0.21 (MOPSlim)	-
*dnaA*	chromosomal replication initiator DnaA	0.80 ± 0.08 (MOPS)	0.67 ± 0.49 (MOPS)
		1.64 ± 0.33 (MOPSlim)	-
*pleC*	sensor histidine kinase PleC, DivK phosphatase	0.77 ± 0.07 (MOPS)	0.75 ± 0.09 (MOPS)
		1.28 ± 0.13 (MOPSlim)	1.02 ± 0.75 (MOPSlim)

Log_2_ change in transcript amount normalized to levels of the SMc01852 mRNA. Errors represent the standard deviation of three replicates. Positions of microarray reporter oligonucleotides relative to the start codon are given in brackets for 5’-UTR regions.

*Description of gene product or associated gene product.

In MOPSlim medium, several upregulated genes were related to cell division and cell wall degradation. Among those involved in cell division we found the *mraZ-mraW* genes (M = +1.30; +1.34) forming an operon with *ftsI*. The first gene of the *dll-ftsQ-ftsA* operon upstream of *ftsZ* (*dll*; M = +1.16) and *mltB2* (M = +1.18), both encoding homologs of peptidoglycan hydrolases, also appeared among the upregulated genes. Interestingly, several differentially expressed genes in the 2011*ecpR1* mutant harbour CtrA binding sites upstream the coding region, like *pleC*, *mraZ*, *mltB2*, and the genes coding for the PilZ-like protein SMc00999, the adenosylhomocystein hydrolase SMc02755, the putative transcriptional regulator SMc01842 and the hypothetical protein SMc03149. Beside this, in both media most of the differentially expressed genes with known functions were also related to metabolism. Among the strongly upregulated genes were the SMb20155-8 operon encoding the components of an ABC transporter (M = +2.57 to +3.22) and SMc03253 coding for an l-proline hydroxylase (M = +2.31). The latter was downregulated 15 min and 1 hour after induction of EcpR1 overproduction (M = -2.98 and -0.84, respectively).

However, looking for an overlap between the top target mRNA predictions (P<0.005) (S1 Table) and genes differentially expressed in the *ecpR1* overexpression or deletion strain (S2–S10 Tables) only genes related to cell cycle were identified.

### EcpR1 post-transcriptionally represses the cell cycle master regulatory genes *gcrA* and *dnaA*

For experimental investigations, we restricted the set of EcpR1 target candidates to genes that fulfilled the following two criteria: (i) prediction by CopraRNA in the *Rhizobiaceae* with P<0.005 and (ii) decrease in transcript abundance upon *ecpR1* overexpression. These included *gcrA*, *dnaA*, *pleC*, *ftsZ*, *ctrA*, *minD*, and SMc00888. To this set we added *divK*, situated in the vicinity of the *ecpR1* locus (Fig 1A), and *divJ*. The corresponding mRNA sequences contain putative thermodynamically favored antisense interactions regions (S8 Fig).

To validate target mRNA candidates of EcpR1 *in vivo*, a double plasmid reporter assay was employed [44]. Target fragments comprising the native 5’-UTR [22] extended by the start codon or by a short 5’-part of the coding region were translationally fused to *egfp* in plasmid pR_EGFP and placed under the control of the constitutive synthetic P_Syn_ promoter [45]. All selected fragments contained the predicted EcpR1 interaction sequences. These plasmids were applied as reporter constructs to determine the post-transcriptional effect of induced EcpR1 overproduction on target mRNAs, while overexpression of the antisense RNA gene SmelC812 was used as control. This approach revealed EcpR1-induced down-regulation of reporter constructs corresponding to the top ranked predicted targets *gcrA* and *dnaA* (P<0.0001, Fig 4) but did not confirm the predicted regulatory effect of EcpR1 on the other cell cycle related target candidates (S7 Fig and S8 Fig). Since fluorescence mediated by the pSMc00888_-235+57_-*egfp* reporter construct did not exceed the background level derived from the empty vector, we were unable to test this gene for EcpR1-induced regulation.

The EcpR1 binding region within the *gcrA* mRNA is located 13 nt downstream the TSS (position -122 relative to the AUG) (Fig 4A). The regulatory effect of EcpR1 on *gcrA* was assessed applying two different reporter constructs comprising the complete 5’-UTR fused to *egfp* either under the control of the constitutive P_Syn_ (plasmid p*gcrA*_-*122+3*_-* egfp*) or the native *gcrA* promoter (plasmid pP*gcrA*_-*122+3*_-*egfp*) (Fig 4A). Compared to the control, induced overexpression of *ecpR1* reduced p*gcrA*_-*122+3*_-*egfp* and pP*gcrA*_-*122+3*_-*egfp* mediated fluorescence to 34% and 42%, respectively (Fig 4A). Furthermore, activity of a chromosomally integrated *gcrA* 3’-*egfp* translational fusion [46] was reduced to 75% in response to *ecpR1* overexpression, validating the two-plasmid assay and confirming that posttranscriptional repression of EcpR1 results in reduction of GcrA protein level.

Five putative EcpR1 binding sites were identified within the *dnaA* mRNA (S1C Fig). Since different alternative ATG start codons have been assigned to *dnaA* in various rhizobial genomes, we affirmed the annotated ATG as translational start of *dnaA* in the Rm1021 genome [47]. None of the alternative start codons were functional when translationally fused to *egfp*. To test the post-transcriptional effect of EcpR1 overproduction on *dnaA* expression various fragments including all predicted binding sites or different subsets were translationally fused to *egfp* under the control of the constitutive P_Syn_ promoter (Fig 4B). Compared to the control, EcpR1 overproduction resulted in decreased activity of all reporter constructs, even when the shortest fragment was tested that only included the putative binding sites 3 and 4, overlapping the RBS and the start codon (Fig 4B).

### A conserved GC-rich loop motif is essential for the regulatory function of EcpR1

Typically, sRNA sequences involved in mRNA base pairing are highly conserved, especially when binding multiple targets [48]. EcpR1 is predicted to fold into a secondary structure consisting of two hairpins (Fig 1A, S1A Fig): the 5’ SL1 domain has a structurally conserved stem loop and a strongly conserved GC-rich loop motif (UCCGCCGCAUCU), which is predicted to be unpaired, while the SL2 domain includes a highly variable stem and a loop that contains the less conserved motif UCCUCG [27]. The predicted interaction region of EcpR1 mapped to the strongly conserved loop motif of SL1 which is part of the prevalent processed 101 nt transcript (Fig 1A, S1A Fig). Overproduction of an EcpR1 version starting from its second 5’-end (EcpR1_5’2_) caused accumulation of this 101 nt core variant and the 142 nt version including the transcription termination sequence, and resulted in cell elongation (S9A and S9C Fig). This indicates that the 29 nt 5’-sequence of the full-length version is not required for provoking this phenotype.

**Fig 5:**
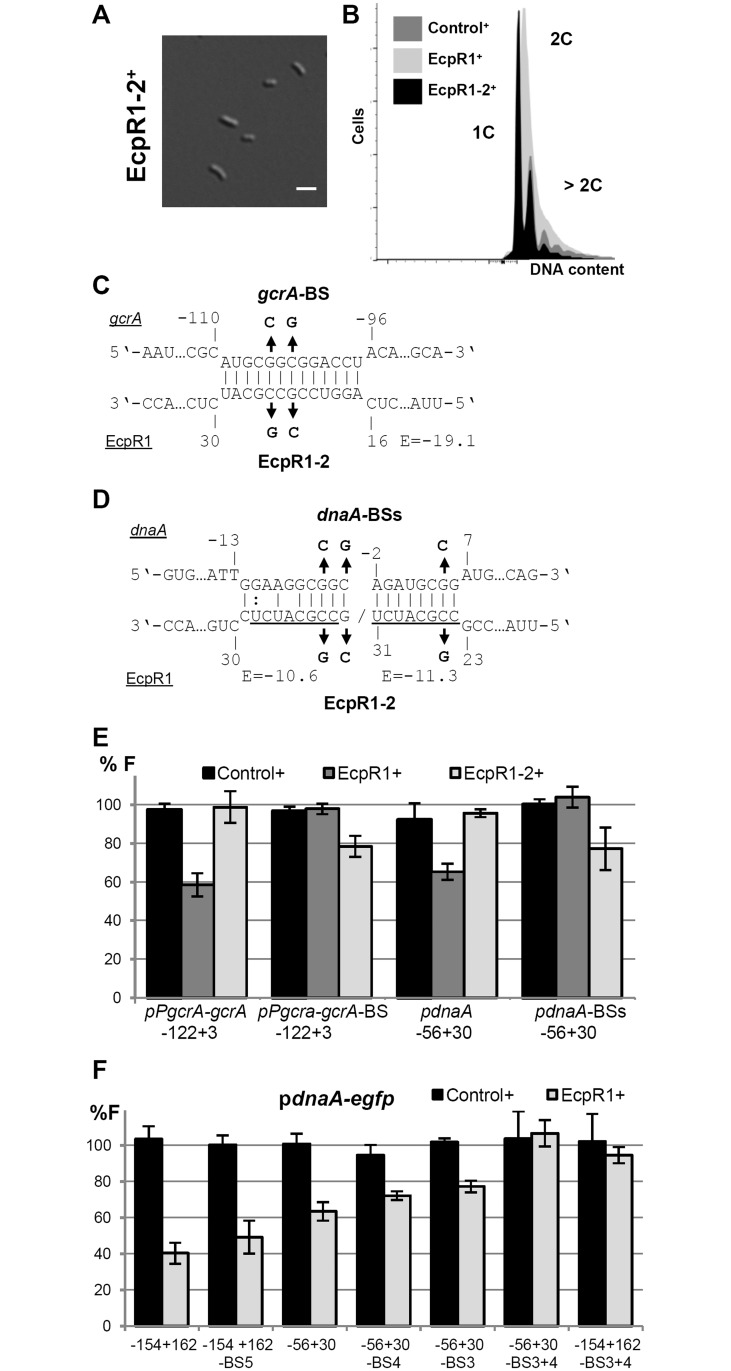
Validation of the predicted EcpR1 binding sites in the *gcrA* and *dnaA* mRNAs. Morphological phenotype **(A)** and DNA content **(B)** of Rm4011*ecpR1* overexpressing *ecpR1-2* carrying 2 nt exchanges in the predicted interaction region. The bar represents 2 μm. **(C, D)** Predicted duplexes between EcpR1 and either *gcrA* or *dnaA* mRNAs. Numbers denote positions relative to the AUG start codon of the mRNA and the second 5’-end of EcpR1. The predicted energy score (E) is indicated in kcal/mol. The nucleotide exchanges in the mRNAs of *gcrA* (*gcrA*-BS-*egfp*) and *dnaA* (p*dnaA*-BSs-*egfp*) as well as in EcpR1 (EcpR1-2) are indicated in bold. **(E, F)** Fluorescence measurements of 4011*ecpR1* co-transformed with *ecpR1*, *ecpR1-2*, or control SmelC812 overexpression plasmids and the indicated reporter plasmids. Reporter constructs carried either native mRNA sequences derived from *gcrA* or *dnaA* or variants with mutations in predicted EcpR1 binding sites (BS). Fragments are delineated in Fig 4. Reporter construct activities were determined as in Fig 4.

Furthermore, overexpression of *ecpR1-2*, a full-length mutant variant carrying changes in 2 nt in the first loop sequence SL1, did not cause the alterations in cell morphology and DNA content previously observed upon overproduction of EcpR1 (Figs 2B–2D and 5A–5E). As EcpR1-2 conserved the predicted secondary structure of EcpR1 and Northern hybridizations confirmed the same level of overproduction of the mutant and the wild type variant (Fig 2A), we exclude that instability of the mutant RNA was responsible for the regulatory deficiency of EcpR1-2. This implies that the GC-rich loop motif is responsible for the cell cycle progression defects observed upon *ecpR1* overexpression. The single substitution G_23_ to C_23_ in EcpR1 (EcpR1-1) was not sufficient to destroy the regulatory activity of this sRNA (S9D Fig).

Moreover, overexpression of *ecpR1-2* did not post-transcriptionally repress *gcrA* and *dnaA* in the same strain background and culture conditions previously applied for EcpR1 (Fig 5E). Concordantly, 2 nt changes in the predicted target region within the *gcrA* 5’-UTR of the reporter fusion construct pP*gcrA*_-*122+3*_-*egfp*, leading to construct pP*gcrA*_-*122+3*_-BS-*egfp*, abolished fluorescence diminution caused by EcpR1 overproduction (Fig 5C and 5E). Introduction of 3 to 5 nt changes into the predicted binding sites 3, 4, or 5 within the *dnaA* mRNA only slightly mitigated the *ecpR1* overexpression-induced repression of reporter construct activities (Fig 5F). In the reporter constructs, substitutions in binding sites 3 and 4 (S1C Fig) were designed to avoid severe effects on translation of the mRNA because these binding sites overlapped the RBS and the start codon. Combined mutations of binding sites 3 and 4 abolished the negative regulatory effect of EcpR1 overproduction on the reporter construct activity (Fig 5F). This implies that the predicted interaction sites 1, 2 and 5 are not required for EcpR1-mediated repression of *dnaA* under the conditions tested.

Combination of the changes in the EcpR1 binding sites within the *gcrA* or *dnaA* 5’-UTRs and EcpR1-2 carrying the compensatory changes in the proposed interaction region partially restored the regulatory function of EcpR1-2. This further confirms the identified interaction regions in sRNA and mRNA (Fig 5B–5E). However, changing CCG to AAT in loop 1 of EcpR1 (EcpR1-3) destroyed its regulatory activity as expected, but the compensatory changes of GGC to TTA in the *gcrA* 5’-UTR did not restore it (S9F Fig). Northern blots showed that levels of EcpR1-3^+^ and EcpR1^+^ are similar (S9A Fig). Lack of restored regulation by compensatory mutations has already been reported for other sRNA-mRNA pairs [49–52] implying that both sequence and structure of the two RNAs are important for their interactions. The changes introduced affect not only the E score of the interaction, which dropped from -19.1 to -14.1, but also the nature of EcpR1 pairing at this position, which probably constitutes the sRNA seed region. This suggests that the binding strength mediated by the GC-rich sequence composition is important for the sRNA-mRNA interaction. Altogether, these data validate *gcrA* and *dnaA* as targets of EcpR1 and strongly suggests that this regulation is mediated through base pairing of the conserved GC-rich, single stranded region of EcpR1 with complementary GC-rich sequences of the target mRNAs.

### EcpR1 function is Hfq-independent and requires RNase E to fully regulate *dnaA*

**Fig 6:**
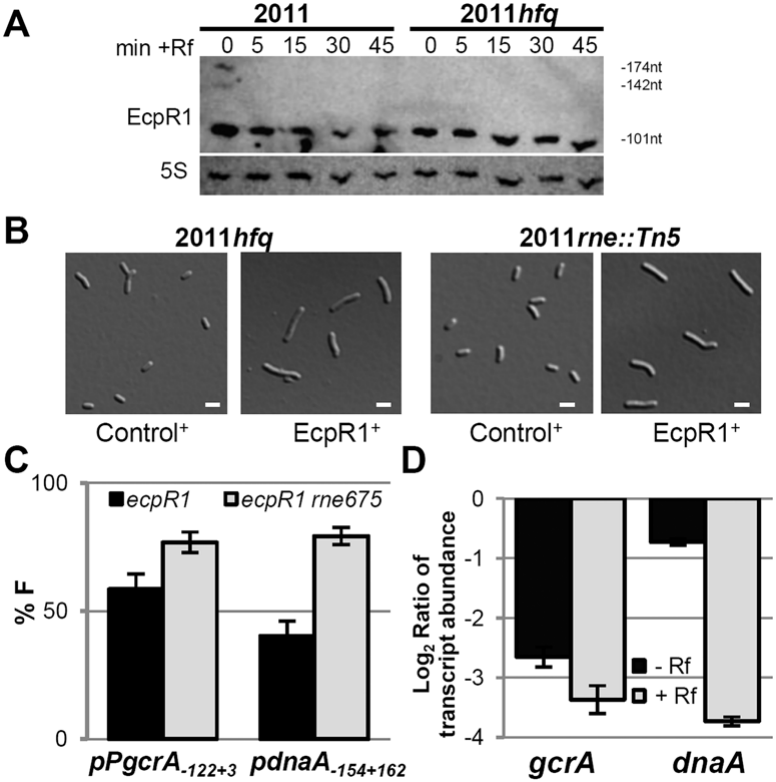
Hfq and RNase E activities are dispensable for EcpR1 overproduction-related cell elongation and post-transcriptional repression of *gcrA*. **(A)** Northern blot analysis of EcpR1 stability in Rm2011 and *hfq* mutant strains grown to early stationary phase (OD_600_ of 1.2, t = 0) and upon transcription arrest with Rf at indicated time points (in min). **(B)** Cell morphology of 2011*hfq* and 2011*rne*::*Tn5* mutants overexpressing either *ecpR1* (EcpR1^+^) or the control RNA gene SmelC812 (Control^+^) upon IPTG induction. Bars represent 2 μm. **(C)** Percentage of fluorescence in EcpR1 overproduction strains relative to the respective control strain overproducing SmelC812 in the Rm4011*ecpR1* or Rm4011*ecpR1 rne675* background co-transformed with plasmids carrying p*PgcrA-gcrA-egfp* or p*dnaA-154+162-egfp* translational fusions. **(D)** qRT-PCR analysis of *gcrA* and *dnaA* transcript abundance in Rm4011*ecpR1* EcpR1^+^ after transcription arrest with Rifampicin for 5 minutes. Values were normalized to the SMc01852 transcript and the levels in the IPTG induced control strain overexpressing the SmelC812 RNA gene. Results from three independent experiments are shown. Error bars indicate the standard deviation.

To further characterize the functional mechanism of EcpR1-dependent post-transcriptional regulation we tested the involvement of the RNA chaperone Hfq and the ribonuclease RNase E in this regulatory mechanism. Hfq is an RNA binding protein that canonically facilitates direct interaction of sRNAs and their mRNA targets and protects them from degradation in the absence of base pairing [53,54]. However, co-immunoprecipitation with epitope-tagged Hfq only detected 14% of the *S*. *meliloti trans*-sRNAs, excluding EcpR1, in cells grown under different stress conditions [55]. Accordingly, absence of Hfq did not compromise EcpR1 stability even 45 min after transcriptional arrest with rifampicin (Rf) as suggested by detection of similar levels of EcpR1 by Northern quantification in the Rm2011 wild type strain and the 2011*hfq* mutant (Fig 6A). In *S*. *meliloti*, knockout of *hfq* compromises growth, metabolism, motility, and stress adaptation in free-living bacteria [56,57]. In our study, microscopy analyses further showed abnormalities in cell morphology with some cells being filamentous and branched. Nevertheless, overexpression of *ecpR1* in 2011*hfq* caused cell elongation that was more severe than in the control (Fig 6B), suggesting that binding to Hfq is not required for EcpR1-mediated regulation of target mRNAs.

sRNAs associate with the C-terminal scaffold region of RNase E and other ribonucleases forming the so-called degradosome, which is recruited through base-pairing to the target mRNA to mediate its cleavage [58]. While the N-terminal catalytic domain of *E*. *coli* RNase E is essential for growth, the C-terminal region is dispensable and its deletion allows for testing the requirement of RNase E in sRNA-induced target mRNA degradation [58]. In *S*. *meliloti*, the C-terminal domain of RNase E is also non-essential, as either a mini-Tn*5* transposon insertion or a plasmid integration into codon 675 of *rne* led to viable cells, though moderately impaired in growth [59]. The 2011*rne*::Tn*5* mutant showed wild type morphology and displayed an elongated phenotype upon overexpression of *ecpR1* (Fig 6B). The same observation was made when comparing EcpR1 overproduction in strain 4011*ecpR1* versus 4011*ecpR1 rne675*. To further investigate whether this endoribonuclease is involved in EcpR1-mediated post-transcriptional regulation, the full-length reporter constructs pP*gcrA*_*122+3*_-*egfp* and p*dnaA*_*154+162*_-*egfp* were introduced to 4011*ecpR1 rne675* containing a plasmid either driving overproduction of EcpR1 or the control RNA SmelC812. A ~20% decrease in *gcrA* and *dnaA* reporter construct-mediated fluorescence was observed in Rm4011 *ecpR1 rne675* overexpressing *ecpR1* as compared to overproduction of SmelC812. In the 4011*ecpR1* strain carrying the complete *rne* gene the difference caused by EcpR1 overproduction was more pronounced for the *dnaA* reporter construct that showed a 39% lower reporter activity (Fig 6C). EcpR1-dependent decay of *gcrA* and *dnaA* mRNAs upon transcriptional arrest was assessed in 4011*ecpR1* either overexpressing *ecpR1* or the control RNA. Whereas decay of the *dnaA* mRNA was ~5-fold higher in the EcpR1 overproducing strain compared to the control strain after transcription inhibition, only a slight ~1.25-fold decrease in *gcrA* transcript levels was observed (Fig 6D). In summary, these data suggest that *dnaA* mRNA-EcpR1 interaction promotes RNase E-dependent mRNA degradation whereas EcpR1-mediated negative post-transcriptional regulation of *gcrA* is mostly independent of mRNA degradation and more likely due to translation inhibition of *gcrA*.

### Altered morphology caused by depletion of GcrA matches the elongated cell phenotype observed after EcpR1 overproduction

Recently, methylation-dependent binding of specific DNA motifs by orthologous GcrA proteins has been reported in several α-proteobacteria including *S*. *meliloti*, suggesting that this transcription regulator is functionally conserved in these bacteria [60]. Attempts to interrupt the *S*. *meliloti* 2011 *gcrA* coding region at the 98^th^ codon by plasmid integration failed to produce any colonies. To further investigate the role of *gcrA*, a deletion mutant was constructed in presence of a plasmid allowing for IPTG-induced expression of an ectopic copy of *gcrA* (2011*gcrA*-P_lac_ *gcrA*) since we also failed to obtain a *S*. *meliloti gcrA* deletion mutant. Strain 2011*gcrA*-P_lac_ *gcrA* was unable to divide in the absence of IPTG, suggesting that *gcrA* may be essential in *S*. *meliloti*.

**Fig 7:**
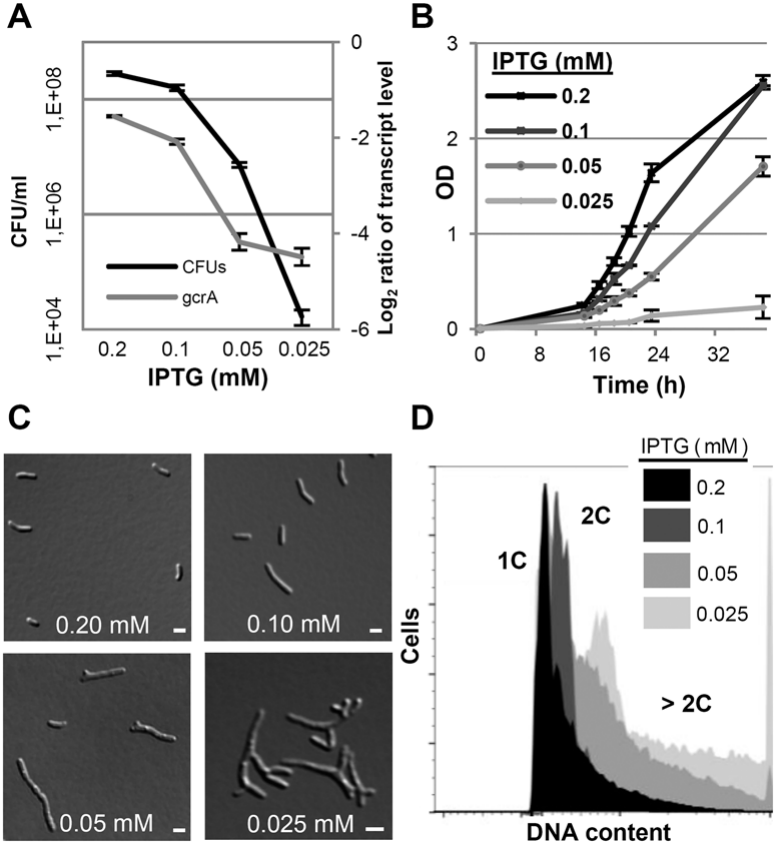
GcrA depletion phenotype in *S*. *meliloti*. qRT-PCR analysis of *gcrA* transcript abundance and colony forming units **(A)**, growth rate **(B)**, morphology phenotypes **(C)** and DNA content **(D)** of Rm2011*gcrA*-P_lac_ *gcrA* subjected to different IPTG concentrations for 16 hours. qRT-PCR values were normalized to the SMc01852 transcript and *gcrA* levels in overnight cultures of Rm2011. 1C and 2C indicate one and two genome equivalents, respectively. Error bars indicate the standard deviation. Bars denote 2 μm.

To study the GcrA depletion phenotype, two independent clones of 2011*gcrA*-P_lac_ *gcrA* were grown in TY rich medium supplemented with 0.5 mM IPTG until early logarithmic phase. Cells were washed and subsequently cultured with different IPTG concentrations leading to lower *gcrA* transcript levels compared to the wild type (Fig 7A). Wild type-like growth was restored at ≥0.2 mM IPTG while lower concentrations hampered growth and cell viability (Fig 7A and 7B). The majority of 2011*gcrA*-P_lac_ *gcrA* cells grown with ≥0.2 mM IPTG displayed wild type-like morphology and harboured one or two genome equivalents. However, bacteria grown with 0.1 mM IPTG became elongated and the DNA content of the cells increased (Fig 7B and 7C). In contrast to the linear filamentous growth of a *C*. *crescentus* temperature sensitive *gcrA* mutant [60], 2011*gcrA*-P_lac_ *gcrA* cells cultured with ≤0.05 mM IPTG showed a tree-shaped morphology characterized by multiple branches (Fig 7C). Interestingly, the decrease in *gcrA* transcript level and the linear filamentous cell morphology observed in the mid-range of the tested IPTG concentrations resembled the phenotypic effects of induced EcpR1 overproduction.

## Discussion

Microorganisms are often facing detrimental conditions unfavorable for cell proliferation such as biotic and abiotic stress factors or nutrient limitation. Therefore regulatory mechanisms adjusting replication initiation and cell cycle progression in response to environmental conditions are crucial for survival. Bacteria have evolved diverse mechanisms to couple perception of stress conditions to a cellular response that triggers a slow down or arrest of cell cycle progression [10]. The most prominent regulatory route for cell cycle control in response to nutrient deprivation involves the stringent response common to diverse bacteria. The stringent response second messenger ppGpp was shown to cause a G1 arrest in *E*. *coli*, *C*. *crescentus*, and *Bacillus subtilis* by modulating abundance or activity of proteins involved in DNA replication, such as DnaA or the primase DnaG. However, the underlying mechanisms are largely unknown. Recently, accumulation of unfolded proteins upon abiotic stress was reported to induce targeted degradation of DnaA resulting in cell cycle arrest in *C*. *crescentus* [61]. Inhibition of cell division mediated by the SOS response was observed in response to DNA damage gaining time for repair. Targeting of divisome components has been shown to be inherent to this DNA damage response in *E*. *coli* and *C*. *crescentus*.

In this study, we add *trans*-sRNA mediated regulation as another layer contributing to these diverse mechanisms linking stress factor sensing to the cell cycle engine. To the best of our knowledge, EcpR1 constitutes the first example of a *trans*-sRNA directly post-transcriptionally modulating expression of two cell cycle related genes in prokaryotes. Despite the effort invested in the model organism *C*. *crescentus* to identify sRNAs exhibiting cell cycle-dependent expression profiles [19], the connection between them and the cell cycle engine remained unproven.

To date, two antisense RNAs related to bacterial cell cycle genes have been identified: the defective prophage-encoded DicF RNA in *E*. *coli*, and asDnaA in *Salmonella enterica*. DicF inhibits translation of the cell-division protein FtsZ when overexpressed [62], while *asdnaA* is expressed in stationary phase and under other stress conditions and seems to increase stability of the *dnaA* mRNA by an unknown mechanism [63]. A few sRNAs have been reported to be involved in bacterial cell differentiation processes that may include modulation of cell cycle control. *trans*-sRNA Pxr negatively regulates fruiting body formation in *Myxococcus* [64]. In *Chlamydia*, the conserved IhtA sRNA translationally inhibits the histone-like protein Hc1 that is involved in compaction of the chromatin into metabolically inert forms during host infection [65,66]. In *E*. *coli*, the plasmid-encoded Rcd RNA indirectly regulates cell growth to ensure plasmid maintenance by binding to a protein involved in indole metabolism [67].

Quick responses to suddenly arising adverse conditions provide an adaptive advantage to the cell. Riboregulators have the potential to act faster as regulatory proteins since RNA is the first product of gene expression. The most prevalent mechanisms of *trans*-sRNA mediated riboregulation affect mRNA translation and stability, which also are most likely the modes of action of EcpR1 on the *gcrA* and *dnaA* target mRNAs in the α-proteobacterium *S*. *meliloti*. We speculate that affecting synthesis of cell cycle master regulators at this post-transcriptional level is an advantageous complementary mechanism to stress-stimulated proteolysis as reported for DnaA in the distantly related α-proteobacterium *C*. *crescentus* [61].

Most sRNAs are conserved only among closely related species, but EcpR1 shows a broad distribution within the Rhizobiales, including organisms with different lifestyles, such as pathogens (e.g. *Agrobacterium)* and diazotrophic plant endosymbionts. EcpR1 overproduction-induced perturbations of cell cycle progression in several species harboring members of the SmelC291 (EcpR1) RNA family also imply functional conservation of this sRNA. However, deletion of *ecpR1* did not cause significant differences in cell growth or viability, but attenuated competitiveness with the wild type. Since sRNAs primarily act to fine-tune stress responses that commonly rely on redundant bacterial pathways [8] sRNA mutants frequently do not show significant phenotypes under laboratory conditions.

The majority of the bacterial sRNAs characterized so far accumulate under stress conditions [68] as does EcpR1, suggesting that this sRNA likely constitutes an adaptive factor that contributes to prevent cell-cycle progression when cells must slow down proliferation. Tight control of EcpR1 levels are likely to be crucial since an excessive amount resulted in a considerable proportion of cells that were not able to resume growth after *ecpR1* overexpression had been stopped. This is in agreement with a more moderate induction of EcpR1 production under stress conditions in the native situation. We obtained evidence that transcription of *ecpR1* driven by an RpoD-type promoter is stimulated by ppGpp, placing EcpR1 in the stringent response regulon of *S*. *meliloti*. This finding is intriguing in light of the role of the stringent response in coupling nutrient status to cell cycle control.

Interestingly, the elongated phenotype of cells overexpressing *ecpR1* resembles that of differentiated nitrogen fixing bacteroids inside plant root nodules and recently, it has been found that nodule-specific cysteine-rich (NCR) peptides triggering rhizobial genome endoreduplication perturbed expression of *dnaA*, *gcrA*, and *ctrA* [69]. In our study, EcpR1 was not detected in *M*. *sativa* mature root nodules implying that *ecpR1* is not expressed in bacteroids. This is in agreement with a transcriptome study of individual zones of the root nodule which determined only low levels of EcpR1 in the symbiotic zone containing mature bacteroids and found the highest concentration of EcpR1 in the interzone where bacteroid differentiation occurs [70].

The confirmed target genes of EcpR1, *dnaA* and *gcrA*, encode key regulators of a complex regulatory circuit governing replication initiation and cell cycle progression. Despite subtle differences, the architecture of this regulatory circuit displays a high degree of similarity in *S*. *meliloti* and *C*. *crescentus* [25,26]. In *C*. *crescentus*, DnaA activates *gcrA* expression [13]. However, computational comparisons did not predict a significant DnaA binding motif in the promoter sequence of the *S*. *meliloti gcrA* gene, but upstream of *divJ* encoding a kinase/phosphatase involved in control of CtrA activity and upstream of SMc00888 encoding a DivK homolog [25,26]. GcrA controls expression of a multitude of target genes including *ctrA*. CtrA-binding motifs have been identified in the promoter regions of *pleC*, *minD*, SMc00888 and *fts*, and the *fla* genes [26]. In *S*. *meliloti*, transcriptome profiling and qRT-PCR assays suggest a direct or indirect effect of EcpR1 overproduction on a number of genes that are core components or known to be under control of this regulatory circuit, further supporting the modulating effect of EcpR1 in the regulatory context of cell cycle control. The enhanced levels of the *dnaA* 5’UTR caused by EcpR1 overproduction may be explained by mechanisms favoring accumulation of the 5’UTR (such as stabilization or attenuation) in conjunction with DnaA autoregulation as reported for *E*. *coli* [71] and feedback regulation increasing levels of DnaA in GcrA-depleted *C*. *crescentus* cells [72]. Such mechanisms may compensate for EcpR1-mediated negative post-transcriptional regulation of *dnaA*. Although significant, transcriptional changes of the cell cycle-related genes were rather low. In the non-synchronized cultures, this might have been due to heterogeneous expression of such genes dependent on the cell cycle state as has been described for *gcrA* in *S*. *meliloti* and other cell cycle-dependent genes whose transcription varies during cell cycle progression [25,46].

Computational target predictions for EcpR1 suggested several cell cycle related target mRNAs among the top 50 candidates (P<0.005), albeit transcriptome and *in vivo* interaction studies only provided evidence for a direct interaction with *gcrA* and *dnaA* mRNAs, ranking in positions 1 and 3, respectively. Still, we cannot exclude that further interactions occur which the two-plasmid assay failed to detect. Similarities between phenotypes caused by EcpR1 overproduction and modest GcrA depletion suggest that a decrease in GcrA concentration contributed to this perturbation of cell cycle progression. DnaA-depletion has been reported to go along with an increase in cell length, while DNA synthesis is arrested [16]. These elongated cells contained only one chromosome, in contrast to the *ecpR1* overexpressing cells that showed an increase in cell length and DNA content.

Although *gcrA* and *dnaA* promoter regions have been extensively studied in *C*. *crescentus* [13,72,73], the functions of the long 5’-UTRs are still unknown in both organisms. Here, we obtained evidence that these 5’-UTRs are involved in *trans*-sRNA mediated post-transcriptional regulation in *S*. *meliloti*. Our experiments indicate that degradation of the *gcrA* mRNA was not significantly promoted by *ecpR1* overexpression. Yet, the output of a reporter gene fused to the *gcrA* 5’-UTR was considerably reduced. This is indicative of EcpR1 rather affecting translational efficiency than stability of the *gcrA* mRNA. However, the single binding site for EcpR1 was identified close to the TSS far upstream of the RBS. sRNA-mediated translational control mostly involves its binding to sequences surrounding the RBS, preventing the ribosome from initiating translation. So far, alternative mechanisms of translational control have been poorly studied, but other models of sRNA repression, such as competing with a “RBS standby site” or pairing with a translation enhancer element have been proposed [74]. In contrast, stability of the *dnaA* mRNA was negatively affected by enhanced levels of EcpR1 and the regulatory effect of this sRNA was significantly alleviated by a C-terminal truncation of RNase E, suggesting that EcpR1 promotes *dnaA* mRNA degradation. Assuming that EcpR1-induced cell cycle perturbation is mainly due to translational inhibition of *gcrA*, these data are in agreement with maintaining this phenotype in the background of the RNase E truncation.

Computational analysis predicted five sequence motifs in the *dnaA* mRNA that are likely to form a stable duplex with EcpR1, which is an exceptionally high number for these types of interactions. Our data strongly suggests that the two binding sites overlapping the RBS and the start codon are sufficient for and synergistically enhance the regulatory effect of EcpR1 on the *dnaA* mRNA under the conditions tested. A conserved GC-rich sequence in loop 1 of EcpR1 was consistently found to be involved in the interactions with these two binding sites in the *dnaA* and one binding site in the *gcrA* mRNA. In bacteria and plants, multiple binding of a target mRNA by a *trans*-sRNA mediated by the same interaction region is a rare finding, although frequently observed for regulatory non-coding RNAs in animals. Binding of multiple target sequences in bacterial mRNAs has been reported, but usually involves different interaction regions of the sRNA. Examples are the MicF sRNA that binds to the *lpxR* mRNA both at the RBS and in the coding sequence [75], as well as the polycistronic mRNA *manXYZ* which is targeted at the RBS and in the intergenic region through overlapping interaction regions of the sRNA SgrS [76].

EcpR1 broadens the unprecedented discovery of prokaryotic sRNA functions of the last two decades. Although additional biological roles of EcpR1 remain to be investigated, stress-induced stimulation of EcpR1 production and its posttranscriptional effect on *gcrA* and *dnaA* suggest an additional level of regulation contributing to a rapid and robust response of the cell cycle machinery to adverse environmental conditions.

## Materials and Methods

### Bacterial strains, plasmids and growth conditions

Bacterial strains and plasmids are listed in S11 Table. *E*. *coli* strains were routinely grown at 37°C in LB medium and rhizobial strains at 30°C in complex tryptone yeast (TY) medium [77] or in modified MOPS-buffered minimal medium [78] (MOPS-MM: MOPS, 10 g l^-1^; mannitol, 10 g l^-1^; NH_4_Cl, 1 g l^-1^; NaCl, 0.1 g l^-1^; MgSO_4_, 0.246 g; CaCl_2_, 250 mM; FeCl_3_•6H_2_O, 10 mg l^-1^; H_3_BO_3_, 3 mg l^-1^; MnSO_4_•4H_2_O, 2.23 mg l^-1^; biotin, 1 mg l^-1^; ZnSO_4_•7H_2_O, 0.3 mg l^-1^; NaMoO_4_•2H_2_O, 0.12 mg l^-1^; CoCl_2_•6H_2_O, 0.065 mg l^-1^, pH 7.2). Nutrient-limiting MOPS (MOPSlim) was modified as follows: mannitol, 2 g l^-1^; NH_4_Cl, 0.3 g l^-1^; NaCl, 0.05 g l^-1^; MgSO_4_, 0.1 g l^-1^. MOPS-C and -N lack mannitol or ammonium chloride, respectively. Antibiotics were added to solid media when required to the following final concentrations (mg/ml): streptomycin (Sm) 100 for *Rhizobium* and 600 for *Sinorhizobium* strains; nalidixic acid (Nx) 10; ampicillin (Ap) 200; tetracycline (Tc) 10; gentamycin (Gm) 40; rifampicin (Rf) 50; chloramphenicol 20; and kanamycin (Km) 50 for *E*. *coli* and *Rhizobium* and 180 for *Sinorhizobium* strains. For liquid cultures, the antibiotic concentration was reduced to 50%. IPTG was added to a final concentration of 0.5 mM to exponential phase cultures (OD_600_ of 0.3 to 0.4), unless other conditions are indicated. For stress induction, media of exponentially growing cultures were modified as described [22] and harvested 1 hour later. Motility assays, were carried out by dispensing 3 μl aliquots of the corresponding bacterial suspension (OD_600_ of 0.9 to 1) on soft agar plates and incubating at 30°C for 5 days. Plant nodulation assays were basically performed as described before [79].

### RNA isolation and northern hybridization

RNA was isolated from bacterial cultures and from 28 days old *M*. *sativa* cv. Eugenia root nodules with the miRNeasy Mini Kit (Qiagen). Nodules covered with liquid nitrogen were ground to powder in a mortar before RNA isolation. For Northern blot detection of RNAs, 4 μg total RNA was separated on 10% polyacrylamide gels containing 7 M urea and transferred onto nylon membranes by semi-dry electroblotting. An EcpR1-specific DIG-labeled DNA probe was used for hybridization (50°C) and detection was performed using the DIG Luminescent Detection Kit (Roche) following the manufactures instructions. Size was determined in relation to an RNA molecular weight marker (NEB).

### Construction of the *S*. *meliloti* mutants and derivative strains

GeneSOEing was used to construct the marker-free deletion of the chromosomal *ecpR1* locus and the strain with mutations in the *ecpR1* σ^70^-dependent promoter -10 region using the internal complementary primers listed in S12 Table. The digested PCR fusion product containing *ecpR1* flanking sequences or the *ecpR1* locus region carrying changes in the promoter -10 region were cloned into suicide vector pK18mobsacB, respectively. Double cross-over events were selected as previously described [80] and checked for the targeted deletion by PCR, sequencing and Northern analyses. To create a conditional depletion mutant, the *gcrA* locus was also deleted by geneSOEing, but this deletion was introduced to *S*. *meliloti* harbouring plasmid pSRKGm containing the *gcrA* gene under control of the IPTG inducible P_lac_ promoter (P_lac_ *gcrA*). Double recombinants were selected on medium supplemented with IPTG and subsequently grown on agar with and without IPTG. Strains exhibiting IPTG-dependent growth were selected and the chromosomal *gcrA* deletion was checked by PCR amplification and sequencing of the *gcrA* chromosomal locus.

For IPTG induced overexpression of *ecpR1* an indirect *sinR*-*sinI* based system was applied. In *S*. *meliloti*, the LuxR-type transcription regulator SinR strongly activates the promoter of the N-acyl homoserine lactone synthase encoding gene *sinI* [81]. The complete sequence of the *sinR* gene and the *sinR-sinI* intergenic region containing the *sinI* promoter were fused to the TSS of the control sRNA gene SmelC812 or the corresponding 5’-end of *ecpR1* by geneSOEing. The resulting fragments were inserted into pSRKKm to generate the expression plasmids that were transferred by conjugation to Rm4011 (*expR* ^-^ *sinI* ^-^) to minimize background expression. A PCR-based mutation strategy was used to replace specific nucleotides within the corresponding plasmid constructs as described before [82] using the internal complementary primers listed in S12 Table.

### eGFP-mediated fluorescence constructs and assays

For construction of *ecpR1* promoter-*egfp* fusions the corresponding genomic fragments (Fig 1B) were amplified and cloned into plasmid pPHUtrap, a derivative of pPHU231 [83] containing a promoterless *sinI* 5’-UTR fused to *egfp*. *S*. *meliloti* cells carrying the *ecpR1* promoter fusions were grown until stationary phase and 100 μl of the cultures were transferred to a 96 well microtiter plate and measured as described below. To accurately compare the activities of the promoter fusions at different OD_600_ values (0.6, 1.2, and 2.8), cells harvested at OD_600_ of 1.2 and 2.8 were diluted to OD_600_ of 0.6 before being transferred to the 96 well microtiter plate for measurement.

To determine EcpR1 target mRNA regulation *in vivo*, plasmid pR_EGFP [44] was used to constitutively express 5’-UTR translational fusions of the predicted target genes from its native TSS [22]. The reporter plasmids were transferred by conjugation to Rm4011*ecpR1* harboring plasmids pSKControl^+^ or pSKEcpR1^+^. Three double transconjugants for each RNA-target fusion combination were grown to mid-exponential phase (OD_600_ of 0.3 to 0.4) and 100 μl aliquots of IPTG treated and untreated cultures were transferred to a 96 well microtiter plate and incubated at 30°C with shaking for 8 hours.

OD_600_, eGFP and mCherry-mediated fluorescence were measured in the Infinite M200 Pro microplate reader (Tecan). Fluorescence values were normalized to the culture OD_600_, and background F/OD ratios from strains harboring the corresponding empty plasmid (pPHUtrap or pR_EGFP) were subtracted from those mediated by each reporter construct.

### Competitive growth assay

For estimation of the relative fitness, Rm2011 and 2011*ecpR1* were labeled with *mCherry* or *egfp* by single integration of either plasmid pKOSm or pKOSe, both pK18mobII derivatives carrying P_T5_:*mCherry* or P_T5_:*egfp* cassettes follow by a T7 terminator site and a 800 bp fragment from *recG*. Strains were individually grown in MOPS or MOPSlim media starter cultures overnight and bacteria were then diluted in the same fresh media to OD_600_ of 0.005 and mixed at a ratio of 1:1 in a final volume of 30 ml. During a 4 weeks period, every seven days of incubation eGFP and mCherry fluorescence of the cultures were measured and the mixed population was diluted 1000-fold in fresh media. One and four weeks after the first mixed inoculation microscopy images were taken to determine the percentage of eGFP- and mCherry-labeled bacteria.

### Microarray-based gene expression profiling

Four independent bacterial cultures of Rm4011 carrying pSKEcpR1^+^ or pSKControl^+^ or either Rm2011 or 2011*ecpR1* were grown in 100 ml of the corresponding medium for each experiment. Cells were harvested in the indicated conditions (15 minutes, 1 hour, and 4 hours after IPTG induction or in the stationary phase of growth) and RNA was isolated. cDNA synthesis, Cy3- and Cy5 labeling, hybridization, image acquisition and data analysis were performed as previously described [84]. Normalization and t-statistics were carried out using the EMMA 2.8.2 microarray data analysis software [85]. Genes and 5’-/3’-UTRs with P-value≤0.05 and M≥0.7 or ≤−0.7 were included in the analysis. The M value represents the log_2_ ratio of both channels. Transcriptome data are available at ArrayExpress (Accession No. E-MTAB-3389).

### Quantitative RT-PCR analysis

qRT-PCR was carried out in a qTOWER Thermal Cycler (Analytik Jena, Germany) using the KAPA SYBR FAST One-Step qRT-PCR Kit and 50 ng of RNA per reaction (5 μl). The ratios of transcript abundance were calculated as the 2^–ΔCT^ mean average of 3 replicates, where CT indicates the level of gene expression in the specified strain relative to the expression in the control strain. The uniformly expressed gene SMc01852 [86] was used to normalize the gene expression data.

### Microscopy

Bacteria were visually examined by differential interference contrast and epifluorescence or highly inclined laminated optical sheet microscopy (Tokunaga) using a Nikon Eclipse Ti-E equipped with 100x CFI Apo TIRF Oil objective (numerical aperture of 1.49) with AHF HC filter sets F36-513 DAPI (excitation band pass 387/11 nm, beam splitter 409 nm, emission band pass 447/60 nm), F36-504 TxRed (ex bp 562/40 nm, bs 593 nm, em bp 624/40 nm) and F36-525 eGFP (exc bp 472/30 nm, beam splitter 495 nm, em bp 520/35 nm). Living cells grown to the desired condition were directly placed on 1% TY agarose pads. Images were acquired with an Andor iXon3 885 EMCCD camera. Image acquisition, measurements and adjustment were done with Nikon NIS elements 4.0 software. For time-lapse analysis images were acquired every 15 minutes at 30°C.

### Fluorescence-activated cell sorting (*FACS)*

To identify DNA content of single cells, 200 μl of culture grown to the desired condition was harvested and fixed in 70% cold ethanol. For examination, fixed cells were washed twice and resuspended in 200 μl of 50 mM sodium citrate buffer, and DNA was stained with 50 μg/ml Hoechst 33342. Acquisition was done on a BD Biosciences LSRII flow cytometer and analyzed using FlowJo 10 software. Each histogram represents the analysis of 50,000 cells.

### Bioinformatics tools

sRNA secondary structures were predicted with RNAfold [36] and represented with VARNA [87]. The full-length EcpR1 sequence was scanned for antisense interactions within several genomes using CopraRNA with standard parameters [28]. *S*. *meliloti* (NC_003047) was included as organism of interest in all rounds of genome-wide target predictions, first together with seven closely related *Rhizobiaceae* species belonging to the genera *Sinorhizobium* (NC_009636, NC_012587), *Agrobacterium* (NC_011985, NC_003063, NC_011988), and *Rhizobium* (NC_007761 and NC_008380). The second group included NC_008254, NC_014923, and NC_002678 from the genus *Mesorhizobium (Phyllobacteriaceae)* and the third group representatives of the *Xanthobacteriaceae* belonging to the genera *Starkeya* (NC_014217), *Xanthobacter* (NC_009720), and *Azorhizobium* (NC_009937). Finally, predictions included the same *Xanthobacteriaceae* representatives together with *Methyocella* (NC_011666) and *Beijerinckia* (NC_010581) (*Beijerinckiaceaceae*), and *Rhodomicrobium* (*Hyphomicrobiaceae*). Predicted individual sRNA-mRNA duplexes were further confirmed with IntaRNA [88] and RNAup [36]. Functional enrichment of EcpR1 top target candidates was assessed applying Fisher’s exact test. For this, the fisher.test function from R statistics [89] was employed with the “alternative” parameter set to “greater”. Based on homology search, 53 *S*. *meliloti* genes are cell cycle related. Of these, 50 are present in the total CopraRNA prediction list (length = 4962) and seven of these 50 are in the top predicted target list (length = 89) at P< = 0.01. In R notation, this leads to the following matrix for fisher.test function: matrix(c(7,43,82,4830),nrow = 2,ncol = 2). The *S*. *meliloti ecpR1*–100 region was BLASTed with default parameters against all currently available bacterial genomes and several regions exhibiting significant similarities (80–100% similarity) were used to generate automated alignments.

## Supporting Information

S1 Table CopraRNA results of *S. meliloti* target candidates predicted for the SmelC291 family (EcpR1) of homologous sRNAs present in closely related *Rhizobiaceae* species belonging to the genera *Sinorhizobium*, *Agrobacterium*, and *Rhizobium*. Cell cycle related candidates used for the enrichment analysis are denoted in bold and experimentally confirmed targets are underlined.
(PDF)

S2 Table Genes and 5’-/3’-UTRs differentially expressed 15 minutes after induction of EcpR1 overproduction (P-value≤0.05 and M≥0.7 or ≤−0.7). The M value represents the log_2_ ratio of transcript levels.
(PDF)

S3 Table Genes and 5’-/3’-UTRs displaying decreased expression 1 hour after induction of EcpR1 overproduction (P-value≤0.05 and M≥0.7 or ≤−0.7). The M value represents the log_2_ ratio of transcript levels.
(PDF)

S4 Table Genes and 5’-/3’-UTRs displaying increased expression 1 hour after induction of EcpR1 overproduction (P-value≤0.05 and M≥0.7 or ≤−0.7). The M value represents the log_2_ ratio of transcript levels.
(PDF)

S5 Table Genes and 5’-/3’-UTRs displaying decreased expression 4 hours after induction of EcpR1 overproduction (P-value≤0.05 and M≥0.7 or ≤−0.7). The M value represents the log_2_ ratio of transcript levels. Cell cycle related candidates are indicated in bold and experimentally confirmed targets are underlined.
(PDF)

S6 Table Genes and 5’-/3’-UTRs displaying increased expression 4 hours after induction of EcpR1 overproduction (P-value≤0.05 and M≥0.7 or ≤−0.7). The M value represents the log_2_ ratio of transcript levels. Cell cycle related candidates are indicated in bold and experimentally confirmed targets are underlined.
(PDF)

S7 Table Genes and 5’-/3’-UTRs displaying decreased expression in 2011*ecpR1* versus Rm2011 wild type growing in MOPS medium (P-value≤0.05 and M≥0.7 or ≤−0.7). The M value represents the log_2_ ratio of transcript levels. Cell cycle related candidates are denoted in bold and experimentally confirmed targets are underlined.
(PDF)

S8 Table Genes and 5’-/3’-UTRs displaying increased expression in 2011*ecpR1* versus Rm2011 wild type growing in MOPS medium (P-value≤0.05 and M≥0.7 or ≤−0.7). The M value represents the log_2_ ratio of transcript levels. Cell cycle related candidates are indicated in bold.
(PDF)

S9 Table Genes and 5’-/3’-UTRs displaying decreased expression in 2011*ecpR1* versus Rm2011 wild type growing in MOPSlim medium (P-value≤0.05 and M≥0.7 or ≤−0.7). The M value represents the log_2_ ratio of transcript levels. Cell cycle related candidates are denoted in bold and experimentally confirmed targets are underlined.
(PDF)

S10 Table Genes and 5’-/3’-UTRs displaying increased expression in 2011*ecpR1* versus Rm2011 wild type growing in MOPSlim medium (P-value≤0.05 and M≥0.7 or ≤−0.7). The M value represents the log_2_ ratio of transcript levels. Cell cycle related candidates are indicated in bold and experimentally confirmed targets are underlined.
(PDF)

S11 Table Bacterial strains and plasmids used in this study. (PDF)

S12 Table Oligonucleotides used in this study. (PDF)

S1 Fig Secondary structure and predicted interaction domains of EcpR1. **(A)** Secondary structure of the EcpR1 171 nt full length variant with a minimum free energy of -82.10 kcal/mol. Nucleotide positions relative to the first 5’-end are shown. SL, stem loop domain. The 13 nt region predicted to bind the *gcrA* mRNA is boxed. Estimated 5’- and 3’-ends of the four different EcpR1 variants are mapped below. **(B)** Visualization of the predicted interaction domains in the EcpR1 full length sequence. The density plot shows the relative frequency of a specific EcpR1 nucleotide position participating in the top predicted target interactions (P≤0.002). The alignments are shown for the top 20 targets in the EcpR1 prediction (*S*. *meliloti* and seven closely related *Rhizobiaceae*). The schematic alignment of homologous sRNAs and targets shows the predicted interaction domains: aligned regions are displayed in grey, gaps in white, and predicted interaction regions in different colors. The *S*. *meliloti* locus tag and gene name (N/A, not available) of the predicted targets are given on the right. **(C)** Predicted EcpR1 binding sites BS1 to BS5 of the *dnaA* mRNA. Nucleotide exchanges in the predicted binding sites BS3 to BS5 that were carried out in different *dnaA* reporter constructs are indicated by arrows and confirmed interactions are shown in bold (see results in Fig 5F). The predicted energy score (E) is indicated in kcal/mol.
(TIF)

S2 Fig Regulation of the *ecpR1* promoter activity. Northern blot detection of the EcpR1 transcript in Rm2011 wild type at different cell densities (OD_600_) in TY medium and minimal medium (MM) or in Rm4011 strain carrying pSKEcpR1^+^ 4 hours after induction with IPTG (EcpR1^+^) **(A)**, in TY medium and 28 days-old mature symbiotic nodules **(B)** and in the 2011Pσ^70^ *ecpR1* strain carrying a mutation in the -10 region of the σ^70^-type promoter in stationary growing and oxygen depleted bacteria in TY medium **(F)**. Plots underneath the Northern blot in (A) represent hybridization signal intensities relative to the level of the EcpR1 101 nt variant in Rm2011 growing in TY rich medium at OD_600_ of 0.6, which has been normalized to 1. Promoter alignment of the EcpR1–100 region in different *Sinorhizobium* strains **(C)**. RNAseq-detected EcpR1 5’-ends in Rm2011 are depicted by arrows and the predicted σ^70^- and σ^54^-dependent promoters are underlined. Nucleotide positions are numbered relative to the 5′1 end. Highly and weakly conserved nucleotides are represented as red or blue letters, respectively. Promoter consensus sequences derived from *S*. *meliloti* 1021 and fragments included in the *ecpR1* transcriptional fusions are indicated below. Means of relative fluorescence intensity values at different cell densities of Rm2011/pP_*ecpR1*__5’2 grown in TY **(D)** and of Rm2011 harbouring the corresponding pP_*ecpR1*_ and the empty vector control, *pleD* or *divK* overexpression plasmids grown in TY medium supplemented with IPTG **(G)**. The standard deviation represents at least three independent measurements of three double transconjugants grown in six independent cultures. Specific activities were normalized to the levels of the stationary phase cultures (OD_600_ of 2.8) (D) or to the cultures lacking IPTG (G) to yield percent relative fluorescence (%F). **(E)** Fluorescence microscopy of exponential and stationary phase Rm2011 cells carrying pP*ecpR1*_5’-2 in TY medium. Bars denote 2 μm.
(TIF)

S3 Fig Effect of *ecpR1* overexpression on *S. meliloti* generation time and recovery from stationary phase. **(A)** Time lapse microscopy of Rm4011 cells overexpressing either the control RNA gene SmelC812 (Control^+^) or *ecpR1* (EcpR1^+^) after addition of IPTG. Bars denote 2 μm. Cell numbers are indicated in brackets. Doubling times of ~2 hours and ~4 hours were determined for the control RNA gene and *ecpR1* overexpressing strains, respectively. **(B)** Abundance of normal-sized and elongated cells in EcpR1^+^ cultures treated with IPTG for 30 hours and proportion of stationary cells that resumed growth after washing of cells and transfer to fresh medium lacking the inductor. Proportions were determined by time-lapse microscopy (n = 500). **(C)** Colony forming units (CFUs) of indicated strains after 3 cycles of re-growing on TY medium supplemented with IPTG for 48 h.
(TIF)

S4 Fig *ecpR1* overexpression-induced phenotype in various α-proteobacteria harbouring an *ecpR1* homolog. Cell morphology **(A)** and DNA content **(B)** of different species overproducing either the control RNA SmelC812 (Control^+^) or EcpR1 (EcpR1^+^) 20 hours after addition of IPTG. Bars denote 2 μm.
(TIF)

S5 Fig 2011*ecpR1* growth and symbiotic phenotypes. Growth rates of Rm2011 and 2011*ecpR1* were compared in TY rich medium at 30°C **(A)**, 45°C **(B)** or after adding 0.4 mM of NaCl **(C)**. Cell viability (CFU/ml) of these two strains was compared after adding 10 mM H_2_O_2_ to logarithmic cultures in TY for 30 minutes or after growing in defined carbon-limited minimal medium (mannitol 2 g l^-1^) for 72 h **(D)**. Error bars indicate the standard deviation of at least two replicates. Symbiotic phenotype of *M*. *truncatula* inoculated with 2011 wild type or 2011*ecpR1*. Time course of *S*. *meliloti*-induced nodule production **(E)**. Percentage of plants developing nodules **(F)**, and showing a Fix^+^ phenotype **(G)**. Shoot length of plants growing in the absence of nitrogen 30 days after inoculation with *S*. *meliloti* 2011, 2011*ecpR1*, and uninoculated (control) **(H)**. Error bars indicate the standard error. All samples were collected from the same experiment (20 plants). Nodulation assays were repeated three times with similar results.
(TIF)

S6 Fig Fitness of Rm2011 wild type vs. 2011*ecpR1* mutant. Representative fluorescence microscopy images **(A-B)** and means of eGFP:mCherry fluorescence ratios **(C-D)** of 2011 *mCherry* mixed with either 2011*egfp* or 2011*ecpR1 egfp* cell cultures at a 1:1 ratio in MOPS **(A, C)** or MOPSlim media **(B, D)** at the indicated time points. Every 7 days the mixed population was diluted 1000-fold in fresh media. Standard deviation represents three determinations of three independent cultures. Bars denote 2 μm.
(TIF)

S7 Fig Target candidates *ctrA*, *minD*, *pleC* and *ftsZ* are not regulated by EcpR1 in *S. meliloti* 4011. Predicted thermodynamically favored antisense interaction regions in *ctrA* **(A)**, *minD* **(B)**, *pleC* **(C)** and *ftsZ1* **(D)** mRNAs, schematic representations of translation fusions to *egfp*, and fluorescence measurements mediated by these constructs in Rm4011*ecpR1* carrying pSKEcpR1^+^ or pSKControl^+^. Numbers denote positions relative to the AUG start codon of the mRNA and the second 5’-end of EcpR1. The predicted energy score (E) is indicated in kcal/mol. The standard deviation represents at least three independent determinations of three double transconjugants grown in six independent cultures.
(TIF)

S8 Fig Target candidates *divJ*, and *divK* are not regulated by EcpR1 in *S. meliloti* 4011. Predicted thermodynamically favored antisense interaction regions in *divJ* **(A)**, SMc00888 **(B)**, and *divK* **(C)** mRNAs, schematic representations of translation fusions to *egfp*, and fluorescence measurements mediated by these constructs in Rm4011*ecpR1* carrying pSKEcpR1^+^ or pSKControl^+^. Numbers denote positions relative to the AUG start codon of the mRNA and the second 5’-end of EcpR1. The predicted energy score (E) is indicated in kcal/mol. The standard deviation represents at least three independent determinations of three double transconjugants grown in six independent cultures.
(TIF)

S9 Fig Molecular and functional characterization of EcpR1 variants starting from the second 5’-end (EcpR1_5’2_), or carrying 1 and 3 nucleotide exchanges in the predicted interaction region (EcpR1-1 and EcpR1-3). Northern blot detection **(A)**, DNA content **(B)** and morphological phenotype **(C)** of Rm4011*ecpR1* overexpressing *ecpR1*_*5’2*_, *ecpR1-1* and *ecpR1-3*, or control SmelC812. The bar represents 2 μm. **(D, F)** Predicted duplexes between EcpR1 and the *gcrA* mRNA. Nucleotide exchanges in EcpR1 and the *gcrA* mutant variants are denoted in bold. **(E, G)** Fluorescence measurements of 4011*ecpR1* co-transformed with *ecpR1*, *ecpR1-1*, *ecpR1-3*, or control SmelC812 overexpression plasmids and the indicated reporter plasmids. The standard deviation represents at least three independent determinations of three double transconjugants grown in six independent cultures.
(TIF)
