# Supplementary material for: A Stress-Induced Small RNA Modulates Alpha-Rhizobial Cell Cycle Progression
Source: PLoS Genet. 2015 Apr 29;11(4):e1005153. doi: 10.1371/journal.pgen.1005153 (PMC4414408; doi:10.1371/journal.pgen.1005153)
Supplement: S1 Table — Cell cycle related candidates used for the enrichment analysis are denoted in bold and experimentally confirmed targets are underlined. (PDF) [file pgen.1005153.s001.pdf]

S1 Table. CopraRNA results of *S. meliloti* target candidates predicted for the SmelC291 family (EcpR1) of homologous sRNAs present in closely related *Rhizobiaceae* species belonging to the genera *Sinorhizobium*, *Agrobacterium*, and *Rhizobium*.

Rank	p-value	Gene ID (Name)	Energy [kcal/mol]	Position mRNA		Position sRNA		Annotation
				start	end	start	end	
**1**	**6.20 E-05**	**SMc02139 (*gcrA*)**	**-17.91**	**92**	**104**	**46**	**58**	cell cycle regulator
2	9.23 E-05	SMa1538	-10.64	181	189	51	59	oxidoreductase
**3**	**9.26 E-05**	**SMc01167 (*dnaA*)**	**-14.41**	**261**	**268**	**49**	**56**	**chromosomal replication initiatior**
4	3.64 E-04	SMc02702	-13.33	291	298	52	59	conserved hypothetical protein
**5**	**3.99 E-04**	**SMc02369 (*pleC*)**	**-11.01**	**199**	**209**	**48**	**60**	**sensor histidine kinase, DivK phosphatase**
6	4.18 E-04	SMa1896 (*msRA3*)	-17.07	238	255	44	63	methionine sulfoxide reductase A
7	4.94 E-04	SMa0204	-20.11	42	58	45	62	sensor histidine kinase
8	6.03 E-04	SMc03957 (*tolR*)	-15.43	235	243	48	56	transport transmembrane protein
9	6.93 E-04	SMb20887	-13.12	159	165	50	56	hypothetical transmembrane protein
10	7.30 E-04	SMc01441 (*hflK*)	-12.12	223	229	50	56	membrane bound protease
11	0.0010	SMc00416	-14.40	8	20	48	59	putative tetrapyrrole methylase
13	0.0012	SMc04210	-9.67	104	114	49	59	conserved hypothetical protein
14	0.0015	SMc01278 (*sugE*)	-11.61	17	31	99	111	chaperone-like protein
15	0.0015	SMa0503	-7.97	198	206	52	61	ABC transporter permease
16	0.0016	SMc02364 (*cycL*)	-16.96	146	157	49	59	cytochrome C-type biogenesis transmembrane protein
17	0.0016	SMc02906	-10.58	6	13	49	56	conserved hypothetical protein
18	0.0017	SMc00612	-14.10	173	184	44	56	hypothetical protein signal peptide
19	0.0019	SMb20719	-11.77	183	206	42	64	sugar uptake ABC transporter permease
**20**	**0.0021**	**SMc04296 (*ftsZ2*)**	**-11.29**	**268**	**274**	**50**	**56**	**cell division protein**
22	0.0024	SMc00088	-12.3	215	227	48	59	2-component receiver domain protein
23	0.0025	SMc04446 (*chvG*)	-7.29	230	250	39	60	signal transduction histidine kinase
24	0.0025	SMb20207 (*pqqD*)	-15.53	141	182	88	134	pyrroloquinoline quinone synthesis protein D
25	0.0026	SMc01919	-15.13	72	81	50	59	hypothetical transmembrane protein
26	0.0026	SMb20499	-13.57	265	271	50	56	glycerol-3-phosphate dehydrogenase
28	0.0027	SMb20329 (*thuA*)	-9.89	212	220	102	110	trehalose catabolism protein
29	0.0027	SMb20497 (*lyx*)	-14.44	291	298	49	56	L-xylulose kinase
30	0.0029	SMa1853	-14.94	102	112	50	60	conserved hypothetical protein
**31**	**0.0032**	**SMc00654 (*ctrA*)**	**-13.28**	**180**	**187**	**49**	**56**	**cell cycle transcriptional regulator**
32	0.0033	SMc03161	-12.24	262	269	49	56	oxidoreductase
33	0.0037	SMc00613 (*acvB*)	-10.36	247	257	52	63	conserved hypothetical protein
34	0.0040	SMc02687	-7.15	90	96	50	56	conserved hypothetical protein
35	0.0043	SMc03150	-11.22	105	147	87	118	transcriptional regulator LuxR family
36	0.0044	SMc01742	-12.84	280	286	50	56	conserved hypothetical protein
37	0.0050	SMb20081	-15.16	225	236	45	56	conserved hypothetical protein
**38**	**0.0053**	**SMb21523 (*minD*)**	**-16.68**	**139**	**151**	**49**	**60**	**putative cell division inhibitor**
39	0.0053	SMc00012 (*ctaG*)	-13.51	203	209	48	54	cytochrome C oxidase assembly protein
40	0.0054	SMc02158	-12.01	240	249	48	56	acyltransferase
41	0.0055	SMc01223 (*lrp3*)	-14.15	99	110	49	59	leucine-responsive regulatory protein. AsnC family
43	0.0056	SMc00582	-9.40	201	207	50	56	conserved hypothetical protein
44	0.0058	SMa5025	-15.25	275	295	44	62	conserved hypothetical protein
46	0.0060	SMc04005 (*pykA*)	-12.55	3	18	48	60	pyruvate kinase
47	0.0060	SMb20712 (*mocB*)	-8.68	167	175	52	60	rhizopine uptake ABC transporter substrate-binding protein precursor
48	0.0060	SMb21089	-11.39	165	187	102	121	carnitine dehydratase
49	0.0061	SMb20877	-12.67	223	229	50	56	oxidoreductase. possibly D-amino acid oxidase
50	0.0061	SMc02039	-11.51	8	33	39	69	oxidoreductase, short-chain dehydrogenase
51	0.0061	SMc01638	-9.88	237	259	42	62	bifunctional 3-hydroxyacyl-CoA dehydrogenase/thioesterase
52	0.0062	SMc02778	-12.52	286	295	49	58	hydroxyacid aldolase
53	0.0064	SMb21078	-11.17	184	191	49	56	glycosyltransferase
54	0.0064	SMa0303	-12.34	276	282	50	56	LysR family transcriptional regulator
**56**	**0.0065**	**SMc01874 (*ftsZ1*)**	**-12.71**	**271**	**277**	**50**	**56**	**cell division protein**
57	0.0066	SMb20633	-9.68	43	49	49	55	sugar uptake ABC transporter permease
58	0.0067	SMc02525 (*fdsB*)	-14.34	228	237	50	59	NAD-dependent formate dehydrogenase subunit beta
59	0.0067	SMc02520 (*glpD*)	-11.42	238	244	50	56	glycerol-3-phosphate dehydrogenase
60	0.0068	SMc02875	-14.01	225	236	49	59	conserved hypothetical protein
61	0.0070	SMc04058	-14.50	63	70	52	59	conserved hypothetical protein
62	0.0071	SMa0834 (*fdxB*)	-8.82	28	35	53	60	FdxB ferredoxin III
63	0.0072	SMc03798 (*galM*)	-8.64	152	158	49	55	aldose 1-epimerase
64	0.0074	SMc04055	-11.77	236	266	42	62	transcriptional regulator LysR family
65	0.0076	SMc02363 (*cycK*)	-10.84	280	289	51	60	cytochrome C-type biogenesis transmembrane protein
66	0.0076	SMa1521	-13.28	133	140	49	56	conserved hypothetical protein
67	0.0079	SMc00175	-15.12	230	242	48	60	ABC transporter ATP-binding protein

68	0.0079	SMb20986 (*narB*)	-14.13	187	194	49	56	nitrate reductase large subunit protein
69	0.0079	SMa1613	-9.19	286	295	55	64	Insertion sequence ISRm29
70	0.0081	SMb20116	-14.31	138	149	49	59	conserved hypothetical protein
71	0.0082	SMb20328 (*thuK*)	-14.45	146	153	49	56	trehalosemaltose transporter ATP-binding protein
73	0.0083	SMc01024 (*secG*)	-10.78	280	286	50	56	preprotein translocase subunit SecG
74	0.0085	SMc02370 (*pepN*)	-12.09	264	279	44	61	aminopeptidase N
75	0.0085	SMc00760 (*recA*)	-11.01	152	159	50	57	DNA strand exchange and recombination protein
76	0.0085	SMc03204 (*lpdA3*)	-7.62	121	132	50	61	dihydrolipoamide dehydrogenase
77	0.0090	SMc02368 (*glnE*)	-11.92	237	246	50	59	glutamate-ammonia-ligase adenylyltransferase
78	0.0092	SMb20300 (*cyaF7*)	-17.00	116	132	48	64	adenylate cyclase
79	0.0093	SMb20523	-8.46	117	123	52	58	diguanylate cyclase signal peptide
80	0.0094	SMc02022	-12.32	277	284	49	56	transcriptional regulator LacI family
81	0.0094	SMb21228	-11.29	199	207	52	60	nucleotide sugar epimerase dehydratase
82	0.0094	SMa0664	-11.46	192	200	48	56	conserved hypothetical protein
83	0.0095	SMc03111 (*pmi*)	-9.99	289	296	102	109	mannose-6-phosphate isomerase
84	0.0095	SMb20500	-11.25	290	298	50	59	aldoketo reductase
85	0.0096	SMb20604	-13.15	39	45	50	56	branched-chain amino acid ABC transporter permease
86	0.0096	SMc00833 (*glcE*)	-10.78	258	264	50	56	glycolate oxidase subunit protein
87	0.0097	SMc01725	-8.39	27	36	48	57	conserved hypothetical transmembrane protein
88	0.0098	SMc02976	-14.57	45	54	49	58	transcriptional regulator MarR family

Cell cycle related candidates used for the enrichment analysis are denoted in bold and experimentally confirmed targets are underlined.
