# Supplementary material for: A Stress-Induced Small RNA Modulates Alpha-Rhizobial Cell Cycle Progression
Source: PLoS Genet. 2015 Apr 29;11(4):e1005153. doi: 10.1371/journal.pgen.1005153 (PMC4414408; doi:10.1371/journal.pgen.1005153)
Supplement: S2 Table — The M value represents the log2 ratio of transcript levels. (PDF) [file pgen.1005153.s002.pdf]

S2 Table. Genes and 5'-/3'-UTRs differentially expressed 15 minutes after induction of EcpR1 overproduction (P-value ≤ 0.05 and M ≥ 0.7 or ≤ −0.7).

Gene ID	Name	Description	M value	Region
Metabolism (3)				
SMc03253		L-proline cis-4-hydroxylase	-2.98	5´UTR
SMc02896	*ilvE1*	Probable branched-chain amino acid aminotransferase	-1.12	3´UTR
SMa1243	*azu1*	Azu1 pseudoazurin	-2.10	CDS
Information storage and processing (1)				
SMc00323		Probable 30S Ribosomal protein S15	-0.72	CDS
Poorly characterized (2)				
SMb20848		Conserved hypothetical membrane protein	-1.94	CDS
SMc00769		Conserved hypothetical protein	-1.28	CDS

Genes were classified according to COG (cluster of orthologous groups).

The M value represents the $\log_2$ ratio of transcript levels.
