# Supplementary material for: A Stress-Induced Small RNA Modulates Alpha-Rhizobial Cell Cycle Progression
Source: PLoS Genet. 2015 Apr 29;11(4):e1005153. doi: 10.1371/journal.pgen.1005153 (PMC4414408; doi:10.1371/journal.pgen.1005153)
Supplement: S3 Table — The M value represents the log2 ratio of transcript levels. (PDF) [file pgen.1005153.s003.pdf]

S3 Table. Genes and 5'-/3'-UTRs displaying decreased expression 1 hour after induction of EcpR1 overproduction (P-value ≤ 0.05 and M ≥ 0.7 or ≤ −0.7).

Gene ID	Name	Description	M value	Region
Cellular processes and signaling (3)				
SMc00168	*sinR*	Qs system, AHL autoinducer-binding, *luxR* family	-1.07	5´UTR
SMc00023		Putative DSBA oxidoreductase	-0.92	CDS
SMc01335	*amiC*	Probable N-acetylmuramoyl-L-alanine amidase	-0.77	5´UTR
Metabolism (12)				
SMb20820	*mocF*	Putative ferredoxin reductase	-3.21	5´UTR
SMb20327	*thuG*	Probable trehalosemaltose transporter permease	-1.88	CDS
SMa0101		Amidohydrolase	-1.59	CDS
SMb20817		Putative transcriptional regulator, LacI family	-1.10	5´UTR
SMc03201	*bkdAa*	Probable 2-oxoisovalerate dehydrogenase	-0.97	CDS
SMc02503		Putative saccharopine dehydrogenase	-0.96	CDS
SMb20326	*thuF*	Probable trehalosemaltose transporter permease	-0.95	CDS
SMb20819	*mocE*	Putative ferredoxin of the Rieseke type protein	-0.91	CDS
SMc03132	*ordL3*	Oxidoreductase	-0.88	CDS
SMc03253		L-proline cis-4-hydroxylase	-0.84	5´UTR
SMc03061	*aglE*	Alpha-glucosides periplasmic ABC transporter	-0.73	CDS
SMb21121	*ivdH*	Putative isovaleryl-CoA dehydrogenase	-0.71	CDS
Information storage and processing (1)				
SMc01336	*rne*	Ribonuclease E	-0.77	5´UTR
Poorly characterized (4)				
SMc03107		Conserved hypothetical protein	-1.26	CDS
SMb20848		Conserved hypothetical membrane protein	-1.17	CDS
SMa1262		Conserved hypothetical pirin-like protein	-1.04	CDS
SMc01449		Conserved hypothetical protein	-0.79	CDS

The M value represents the $\log_2$ ratio of transcript levels.
