# Supplementary material for: A Stress-Induced Small RNA Modulates Alpha-Rhizobial Cell Cycle Progression
Source: PLoS Genet. 2015 Apr 29;11(4):e1005153. doi: 10.1371/journal.pgen.1005153 (PMC4414408; doi:10.1371/journal.pgen.1005153)
Supplement: S4 Table — The M value represents the log2 ratio of transcript levels. (PDF) [file pgen.1005153.s004.pdf]

S4 Table. Genes and 5'-/3'-UTRs displaying increased expression 1 hour after induction of EcpR1 overproduction (P-value ≤ 0.05 and M ≥ 0.7 or ≤ −0.7).

Gene ID	Name	Description	M value	Region
Cellular processes and signaling (2)				
SMc03005		Conserved hypothetical protein	1.69	CDS
SMc02833	*mepA*	Putative murein endopeptidase transmembrane	1.58	5´UTR
Metabolism (13)				
SMa0203		Putative ABC transporter	2.59	CDS
SMb20712	*ibpA*	Putative myo-inositol ABC transporter	2.14	5´UTR
SMa0077		Hypothetical protein	1.71	CDS
SMb20712	*mocB*	Putative rhizopine uptake ABC transporter precursor	1.40	CDS
SMc00432	*iolB*	Putative myo-inositol catabolism protein	1.27	CDS
SMb21338		Putative molybdopterin binding protein	1.12	CDS
SMc00549		Hypothetical protein	1.10	CDS
SMb20072		ABC transporter, periplasmic solute-binding	0.92	5´UTR
SMc02520	*glpD*	Putative glycerol-3-phosphate dehydrogenase	0.88	CDS
SMc00781	*iolA*	Methylmalonate-semialdehyde dehydrogenase	0.82	CDS
SMc00433	*iolE*	Putative myo-inositol catabolism protein	0.78	CDS
SMc02518		Putative glycerol-3-phosphate ABC transporter	0.73	CDS
SMc03858	*pheAa*	Putative chorismate mutase	0.71	CDS
Information storage and processing (11)				
SMc02489	*xerC*	Putative tyrosine recombinase	2.40	5´UTR
SMb20847		Putative DNA-binding transcriptional regulator	1.49	CDS
SMc01164	*iolR*	Putative transcriptional repressor of myo-inositol	0.87	5´UTR
SMc01310	*rpsJ*	Probable 30S ribosomal protein S10	0.83	CDS
SMc01298	*rplX*	Probable 50S ribosomal protein L24	0.79	CDS
SMc01309	*rplC*	Probable 50S ribosomal protein L3	0.78	CDS
SMc03859	*rpsP*	Probable 30S ribosomal protein S16	0.78	CDS
SMc01308	*rplD*	Probable 50S ribosomal protein L4	0.76	CDS
SMc00335	*rpsA*	30S ribosomal protein S1	0.73	5´UTR
SMc02101	*rpsB*	Probable 30S ribosomal protein S2	0.71	CDS
SMc01299	*rplN*	Probable 50S ribosomal protein L14	0.70	CDS
Poorly characterized (10)				
SMc01108		Putative tRNA (guanine-N(7)-)-methyltransferase	2.36	3´UTR
SMc02221		Conserved hypothetical protein	1.98	5´UTR
SMc01163		Putative oxidoreductase	1.46	CDS
SMc01200		Transmembrane protein. Periplasm Signal	1.43	CDS
SMb20422		Putative alcohol dehydrogenase	1.35	3´UTR
SMc02056		Conserved outer membrane protein	1.31	CDS
SMc00656		Acyl-CoA N-acyltransferase.	1.18	CDS
SMb20899	*idhA*	Hypothetical myo-inositol dehydrogenase protein	0.83	CDS
SMc00332		Hypothetical protein	0.72	CDS
SMc02051		Conserved hypothetical protein	0.71	CDS

The M value represents the $\log_2$ ratio of transcript levels.
