# Supplementary material for: A Stress-Induced Small RNA Modulates Alpha-Rhizobial Cell Cycle Progression
Source: PLoS Genet. 2015 Apr 29;11(4):e1005153. doi: 10.1371/journal.pgen.1005153 (PMC4414408; doi:10.1371/journal.pgen.1005153)
Supplement: S5 Table — The M value represents the log2 ratio of transcript levels. Cell cycle related candidates are indicated in bold and experimentally confirmed targets are underlined. (PDF) [file pgen.1005153.s005.pdf]

S5 Table. Genes and 5'-/3'-UTRs displaying decreased expression 4 hours after induction of EcpR1 overproduction (P-value ≤ 0.05 and M ≥ 0.7 or ≤ −0.7).

Gene ID	Name	Description	M value	Region
Cellular processes and signaling (6)				
**SMc00888**		**Putative 2-component receiver domain protein**	**-0.99**	**CDS**
**SMc00887**		**Diguanylate cyclase/phosphodiesterase**	**-0.90**	**CDS**
SMc03090	*chew3*	Putative chemotaxis protein	-0.87	5´UTR
SMc00023		Putative DSBA oxidoreductase	-0.74	CDS
SMc02868		Putative multidrug efflux system	-0.73	5´UTR
**SMc00059**	***divJ***	**Histidine protein kinase (cell division)**	**-0.71**	**CDS**
Metabolism (11)				
SMc01020		Putative oxidoreductase	-3.48	CDS
SMb20338		Putative transporter	-2.16	CDS
SMb20484	*supA*	Putative ABC transporter periplasmic sugar-binding	-1.65	5´UTR
SMb21168		Putative inner membrane transport protein	-1.52	CDS
SMa1163		Cation transport P-type ATPase	-0.95	CDS
SMc02677	*proC*	Probable pyrroline-5-carboxylate reductase	-0.87	CDS
SMc01971	*puuC*	Putative gamma-glutamyl-gamma-aminobutyraldehyde dehydrogenase	-0.80	5´UTR
SMa0855	*nodP1*	ATP-sulfurylase small subunit	-0.75	5´UTR
SMb21192	*cbbA2*	Putative fructose-bisphosphate aldolase	-0.71	3´UTR
SMc04386	*aatB*	Aspartate aminotransferase B	-0.70	3´UTR
Information storage and processing (9)				
SMc01336	*rne*	Ribonuclease E	-1.78	5´UTR
SMc02851		Transcriptional regulator, MarR family	-1.36	CDS
SMb20665		Partial transposase of insertion ISRm17 protein	-1.13	CDS
**SMc00021**	***ccrM***	**Cell cycle-regulated adenine DNA methyltransferase**	**-1.13**	**3´UTR**
SMc00289	*cspA5*	Putative cold shock transcription regulator	-0.89	5´UTR
SMc03958	*tolQ*	Putative transport transmembrane protein	-0.82	5´UTR
SMc01714		Hypothetical transmembrane protein	-0.77	CDS
SMc00108		Putative acetyltransferase	-0.76	5´UTR
SMc03874		Hypothetical protein	-0.75	5´UTR
Cell motility (8)				
SMc03038	*flaB*	Flagellin B protein	-2.51	CDS
SMc03037	*flaA*	Flagellin A protein	-2.32	CDS
SMc03049	*flgL*	Putative flagellar hook-associated protein	-1.92	CDS
SMc04114	*pilA1*	Putative pilin subunit protein	-1.36	CDS
SMc03051	*flbT*	Putative flagellin synthesis repressor protein	-1.19	CDS
SMc03052	*flbD*	Putative basal-body rod modification protein	-1.09	CDS
SMc03039	*flaD*	Flagellin D protein	-0.90	CDS
SMc03050	*flaF*	Putative flagellin synthesis regulator	-0.87	CDS
Poorly characterized (18)				
SMc03107		Conserved hypothetical protein	-2.83	CDS
SMb20848		Conserved hypothetical membrane protein	-2.34	CDS
SMc00239		Conserved hypothetical transmembrane protein	-2.30	CDS
SMc01001		Hypothetical transmembrane protein	-2.04	5´UTR
SMcORF4		Hypothetical protein	-1.94	5´UTR
SMb21211		Putative membrane protein	-1.41	5´UTR
SMc03746		Hypothetical protein	-1.28	CDS
SMa0661		Conserved hypothetical protein	-0.98	5´UTR
SMa0667		Conserved hypothetical protein	-0.93	5´UTR
SMc02392		Hypothetical protein	-0.87	5´UTR
SMc03161		Glucose-fructose oxidoreductase	-0.83	5´UTR
<u>**SMc02139**</u>	<u>***gcrA***</u>	<u>**Cell cycle regulator**</u>	<u>**-0.76**</u>	<u>**5´UTR**</u>
SMc00507		Hypothetical protein	-0.76	CDS
SMa0907		Conserved hypothetical protein	-0.75	5´UTR
SMc03832		Conserved hypothetical signal peptide protein	-0.72	CDS
SMc04010		Conserved hypothetical protein	-0.71	5´UTR
SMb22001	*msbA2*	Surface saccharide ABC transporter ATP-binding	-0.70	3´UTR
SMc03013		Conserved hypothetical protein	-0.70	CDS

The M value represents the $\log_2$ ratio of transcript levels.

Cell cycle related candidates are indicated in bold and experimentally confirmed targets are underlined.
