# Supplementary material for: A Stress-Induced Small RNA Modulates Alpha-Rhizobial Cell Cycle Progression
Source: PLoS Genet. 2015 Apr 29;11(4):e1005153. doi: 10.1371/journal.pgen.1005153 (PMC4414408; doi:10.1371/journal.pgen.1005153)
Supplement: S6 Table — The M value represents the log2 ratio of transcript levels. Cell cycle related candidates are indicated in bold and experimentally confirmed targets are underlined. (PDF) [file pgen.1005153.s006.pdf]

S6 Table. Genes and 5’-/3’-UTRs displaying increased expression 4 hours after induction of EcpR1 overproduction (P-value ≤ 0.05 and M ≥ 0.7 or ≤ −0.7).

Gene ID	Name	Description	M value	Region
Cellular processes and signaling (2)				
SMc02833	*mepA*	Putative murein endopeptidase transmembrane	1.15	5´UTR
SMc03941		Conserved hypothetical transmembrane protein	0.86	5´UTR
Metabolism (10)				
SMa0203		ABC transporter	3.73	CDS
SMc01631		ABC transporter ATP-binding protein	3.51	CDS
SMa0077		Galactose mutarotase and related enzyme	1.62	CDS
SMc00549		Hypothetical protein	1.35	CDS
SMb21338		Putative molybdopterin binding protein	1.14	CDS
SMc03776	*proB1*	Probable glutamate 5-kinase	0.95	CDS
SMa1408		Putative dehydratase	0.84	CDS
SMc00781	*iolA*	Methylmalonate-semialdehyde dehydrogenase	0.83	CDS
SMc00595	*ndk*	Probable nucleoside diphosphate kinase	0.81	CDS
SMc02686	*prsA*	Probable ribose-phosphate pyrophosphokinase	0.73	CDS
Information storage and processing (5)				
SMb20847		Probable transcriptional regulator	1.65	CDS
**SMc01167**	***dnaA***	**Chromosomal replication initiator**	**1.42**	**5´UTR**
SMc02912	*nusA*	Probable N utilization substance protein A	1.04	CDS
SMa0181	*cspA7*	CspA7 cold shock protein	0.89	CDS
SMc02802		Putative DNA polymerase III, delta subunit	0.80	CDS
Poorly characterized (8)				
SMc01518		Hypothetical protein	2.82	CDS
SMc00656		Conserved hypothetical protein	2.64	CDS
SMc02221		Conserved hypothetical protein	2.32	5´UTR
SMc01200		Conserved hypothetical protein, required for motility	2.25	CDS
SMc02056		Conserved hypothetical protein	1.59	CDS
SMc03005		Hypothetical transmembrane protein	1.18	CDS
SMc00591		Conserved hypothetical protein	0.86	CDS
SMa0649		Hypothetical/unknown signal peptide protein	0.82	CDS

The M value represents the $\log_2$ ratio of transcript levels.
Cell cycle related candidates are indicated in bold and experimentally confirmed targets are underlined.
