# Supplementary material for: A Stress-Induced Small RNA Modulates Alpha-Rhizobial Cell Cycle Progression
Source: PLoS Genet. 2015 Apr 29;11(4):e1005153. doi: 10.1371/journal.pgen.1005153 (PMC4414408; doi:10.1371/journal.pgen.1005153)
Supplement: S7 Table — The M value represents the log2 ratio of transcript levels. Cell cycle related candidates are denoted in bold and experimentally confirmed targets are underlined. (PDF) [file pgen.1005153.s007.pdf]

S7 Table. Genes and 5'-/3'-UTRs displaying decreased expression in 2011*ecpR1* versus Rm2011 wild type growing in MOPS medium (P-value ≤ 0.05 and M ≥ 0.7 or ≤ −0.7).

Gene ID	Name	Description	M value	Region
Cellular processes and signaling (4)				
SMc00999		Type IV pilus assembly PilZ-like protein	-0.79	CDS
SMc01410		Putative lipoprotein transmembrane	-0.76	5´UTR
**<u>SMc01167</u>**	***<u>dnaA</u>***	**<u>Chromosomal replication initiator</u>**	**<u>-0.74</u>**	**<u>5´UTR</u>**
SMc02833	*mepA*	Putative murein endopeptidase transmembrane protein	-0.67	5´UTR
Metabolism (7)				
SMb21438		TRAP-type transporter, periplasmic solute-binding	-1.30	CDS
SMc02450	*argJ*	Probable Arginine biosynthesis bifunctional protein	-0.84	CDS
SMc04239		Hypothetical protein	-0.83	5´UTR
SMc04028	*gltB*	Probable glutamate synthase NADPH large chain	-0.80	5´UTR
SMb21635	*paaZ*	Aldehyde dehydrogenase (NAD+)	-0.76	CDS
SMc04294		Putative peptide ABC transporter	-0.73	CDS
SMc01118		Putative peptidase	-0.68	CDS
Information storage and processing (2)				
SMb20162		Probable transcriptional regulator	-1.28	5´UTR
SMb21601		Putative transcriptional regulator	-0.77	5´UTR
Poorly characterized (5)				
SMb20273		Hypothetical protein	-1.05	5´UTR
SMb21456		Hypothetical protein	-0.99	CDS
SMb21413		Hypothetical protein	-0.83	CDS
SMa0604		Conserved hypothetical protein	-0.79	5´UTR
SMb20010		Predicted hydrolase or acyltransferase	-0.71	CDS

The M value represents the log_2 ratio of transcript levels.
Cell cycle related candidates are denoted in bold and experimentally confirmed targets are underlined.
