# Supplementary material for: A Stress-Induced Small RNA Modulates Alpha-Rhizobial Cell Cycle Progression
Source: PLoS Genet. 2015 Apr 29;11(4):e1005153. doi: 10.1371/journal.pgen.1005153 (PMC4414408; doi:10.1371/journal.pgen.1005153)
Supplement: S8 Table — The M value represents the log2 ratio of transcript levels. Cell cycle related candidates are indicated in bold. (PDF) [file pgen.1005153.s008.pdf]

S8 Table. Genes and 5'-/3'-UTRs displaying increased expression in 2011*ecpR1* versus Rm2011 wild type growing in MOPS medium (P-value ≤ 0.05 and M ≥ 0.7 or ≤ −0.7).

Gene ID	Name	Description	M value	Region
Cellular processes and signaling (4)				
SMc01187	*rlpA*	Putative rare lipoprotein A precursor	0.82	CDS
SMc04205		Putative iron/heme transport protein	0.77	CDS
**SMc02369**	***pleC***	**Sensor histidine kinase, DivK phosphatase**	**0.75**	**CDS**
SMc00399	*corA1*	Probable magnesium and cobalt transport	0.72	5´UTR
Metabolism (8)				
SMc01875	*lpxc*	Probable N-acetylglucosamine deacetylase	0.97	5´UTR
SMc00231	*glmS*	Glucosamine--fructose-6-phosphate	0.90	CDS
SMc02124	*cysI*	Putative sulfite reductase	0.88	CDS
SMc02093	*lpxD*	Probable glucosamine N-acyltransferase	0.83	CDS
SMc03179	*phaA1*	PH adaptation potassium efflux system transmembrane	0.78	CDS
SMc02123		Sulfate or sulfite assimilation protein	0.75	CDS
SMb20151		Protein-tyrosine phosphatase	0.77	CDS
SMc01880	*panC*	Probable pantoate--beta-alanine ligase	0.71	CDS
Information storage and processing (4)				
SMc01294	*rplF*	Probable 50S ribosomal protein L6	1.84	CDS
SMc02551	*cysS*	Probable cysteinyl-tRNA synthetase	0.78	5´UTR
SMc01268	*lipB*	Probable octanoyltransferase	0.77	CDS
SMa1749		Putative transcriptional regulator	0.75	5´UTR
Poorly characterized (11)				
Sma1200		Conserved hypothetical protein	0.94	5´UTR
SMc02582		Conserved hypothetical protein	0.82	CDS
SMa1608		Conserved hypothetical protein	0.82	3´UTR
SMa1803		TRm2011-2a transposase	0.75	CDS
SMb21090		Conserved hypothetical membrane-anchored protein	0.75	CDS
SMc04178		Conserved hypothetical protein	0.73	CDS
SMc01637		Conserved hypothetical protein	0.72	CDS
SMc02221		Conserved hypothetical protein	0.72	CDS
SMa1585		Hypothetical protein	0.70	CDS
SMc02735		Hypothetical protein	0.70	CDS

The M value represents the $\log_2$ ratio of transcript levels.

Cell cycle related candidates are indicated in bold and experimentally confirmed targets are underlined.
