# Supplementary material for: A Stress-Induced Small RNA Modulates Alpha-Rhizobial Cell Cycle Progression
Source: PLoS Genet. 2015 Apr 29;11(4):e1005153. doi: 10.1371/journal.pgen.1005153 (PMC4414408; doi:10.1371/journal.pgen.1005153)
Supplement: S9 Table — The M value represents the log2 ratio of transcript levels. Cell cycle related candidates are denoted in bold and experimentally confirmed targets are underlined. (PDF) [file pgen.1005153.s009.pdf]

S9 Table. Genes and 5'-/3'-UTRs displaying decreased expression in 2011*ecpR1* versus Rm2011 wild type growing in MOPSlim medium (P-value ≤ 0.05 and M ≥ 0.7 or ≤ −0.7).

Gene ID	Name	Description	M value	Region
Metabolism (6)				
SMa0376		Hydrolase	-1.44	CDS
SMb21224	*nodQ*	Putative sulfate adenylyltransferase	-0.80	CDS
SMc00873	*kup1*	Probable KUP system potassium uptake protein	-0.76	CDS
SMb21223	*nodP2*	Putative sulfate adenylyltransferase subunit 2 protein	-0.73	CDS
SMa0203		Putative ABC transporter	-0.72	CDS
SMa0855	*nodP1*	Sulphate adenylyltransferase, small subunit	-0.70	CDS
Cellular processes and signaling (3)				
**Smc01167**	***dnaA***	**Chromosomal replication initiator**	**-0.89**	**5´UTR**
SMb21240		Putative MPA1 family protein	-0.78	CDS
SMc00047	*bla*	Putative beta-lactamase signal peptide protein	-0.71	CDS
Information storage and processing (1)				
SMb20078		Probable response regulator	-0.86	3´UTR
Poorly characterized (7)				
SMc03149		Hypothetical protein	-1.69	5´UTR
SMb20829		Putative secreted calcium-binding protein	-1.15	CDS
SMc00281		Hypothetical signal peptide protein	-0.97	CDS
SMb20518		Predicted chitinase	-0.92	3´UTR
SMa0380		Conserved hypothetical protein	-0.88	CDS
SMb20079		Probable response regulator	-0.79	3´UTR
SMa0364		Hypothetical protein	-0.75	CDS
SMc00734		Probable transporter	-0.74	CDS
SMb20841		Hypothetical membrane-anchored protein	-0.72	CDS

The M value represents the log_2 ratio of transcript levels.

Cell cycle related candidates are denoted in bold and experimentally confirmed targets are underlined.
