# Supplementary material for: A Stress-Induced Small RNA Modulates Alpha-Rhizobial Cell Cycle Progression
Source: PLoS Genet. 2015 Apr 29;11(4):e1005153. doi: 10.1371/journal.pgen.1005153 (PMC4414408; doi:10.1371/journal.pgen.1005153)
Supplement: S10 Table — The M value represents the log2 ratio of transcript levels. Cell cycle related candidates are indicated in bold and experimentally confirmed targets are underlined. (PDF) [file pgen.1005153.s010.pdf]

S10 Table. Genes and 5'-/3'-UTRs displaying increased expression in 2011*ecpR1* versus Rm2011 wild type growing in MOPSlim medium (P-value ≤ 0.05 and M ≥ 0.7 or ≤ −0.7).

Gene ID	Name	Description	M value	Region
Cellular processes and signaling (8)				
**SMc02369**	***pleC***	**Sensor histidine kinase, DivK phosphatase**	**1.02**	**CDS**
SMb20941	*exsA*	MsbA-like saccharide exporting ABC transporter protein	0.82	5´UTR
SMa1337		ABC transporter, periplasmic solute-binding protein	0.76	CDS
SMc00794		Putative 2-component receiver domain protein	0.75	5´UTR
SMa1686		Two-component response regulator	0.73	CDS
SMc00185		Putative ABC transporter ATP-binding transmembrane	0.72	5´UTR
SMc00857	*sohB*	Probable proteinase	0.71	5´UTR
SMa0114		Putative sensory transduction regulatory protein	0.70	CDS
Metabolism (11)				
SMb20158		Hypothetical ABC transporter	3.22	CDS
SMb20156		Putative ABC transporter ATP-binding protein	3.11	CDS
SMb20157		Hypothetical ABC transporter	2.89	CDS
SMb20155		Putative ABC transporter permease	2.57	5´UTR
SMc03253		L-proline cis-4-hydroxylase	2.31	CDS
SMa1223	*fixM*	Flavoprotein oxidoreductase	1.87	CDS
SMb20151		Protein-tyrosine phosphatase	1.52	CDS
SMc01845	*mltB2*	Membrane-bound lytic murein transglycosylase B	1.18	CDS
SMc01871	*ddl*	Probable D-alanine ligase, cell shape wall biogenesis	1.16	CDS
SMc02755	*ahcY*	Adenosylhomocysteina hydrolase	0.81	5´UTR
SMb20316		ABC transporter, periplasmic solute-binding protein	0.73	3´UTR
Information storage and processing (13)				
SMa1225	*fixK1*	Transcriptional regulator	1.86	CDS
SMa0762	*fixK2*	Transcriptional regulator	1.41	CDS
**SMc01858**	***mraW***	**S-adenosyl-L-methionine-dependent methyltransferase**	**1.34**	**CDS**
**SMc01857**	***mraZ***	**Cell division protein**	**1.30**	**CDS**
SMc01260		Putative transcriptional regulator, merR family protein	1.07	3´UTR
SMc03863	*rplS*	Probable 50S ribosomal protein L19	1.02	5´UTR
SMc01295	*rpsH*	Probable 30S ribosomal protein S8	0.82	CDS
SMa0789		Transcriptional regulator, GntR family	0.82	CDS
SMc01842		Probable transcriptional regulator	0.82	CDS
SMc01295	*rpsH*	Probable 30S ribosomal protein S8	0.82	CDS
SMa0789		Transcriptional regulator, GntR family	0.82	CDS
**SMc00561**	***dnaB***	**Probable replicative DNA helicase**	**0.75**	**5´UTR**
SMc01287	*rpsM*	Probable 30S ribosomal protein S13	0.70	CDS
Poorly characterized (12)				
SMb21285		Transthyretin-like protein	1.33	CDS
SMc01902		Hypothetical protein	0.95	CDS
SMb20041		Hypothetical protein	0.90	5´UTR
SMb20222		Hypothetical protein	0.84	5´UTR
SMc02401		Hypothetical transmembrane protein	0.83	3´UTR
SMa1178		Conserved hypothetical protein	0.80	CDS
SMc02081		Hypothetical protein	0.78	5´UTR
SMc00800		Hypothetical transmembrane protein	0.75	CDS
SMb21281		Putative integral membrane transporter protein	0.75	5´UTR
SMb20066		Hypothetical protein	0.71	5´UTR
SMb20082		Conserved hypothetical protein	0.74	CDS
SMa2237		Hypothetical protein	0.74	5´UTR

The M value represents the $\log_2$ ratio of transcript levels.

Cell cycle related candidates are indicated in bold and experimentally confirmed targets are underlined.
