# Supplementary material for: A Stress-Induced Small RNA Modulates Alpha-Rhizobial Cell Cycle Progression
Source: PLoS Genet. 2015 Apr 29;11(4):e1005153. doi: 10.1371/journal.pgen.1005153 (PMC4414408; doi:10.1371/journal.pgen.1005153)
Supplement: S11 Table — (PDF) [file pgen.1005153.s011.pdf]

S11 Table. Bacterial strains and plasmids used in this study.

Strain/Plasmid	Relevant Characteristics	Source or reference
S. meliloti		
Rm2011	Nx^r, Sm^r, *expR*$^-$; Sm resistant derivative of *S. meliloti* SU47. Referred to as wild type in this study.	[90]
2011*ecpR1*	*ecpR1* deletion mutant	This work
2011Pσ70*ecpR1*	2011 carrying changes in the *ecpR1* σ^{70}-dependent promoter -10 region	This work
2011*rpoN::Tn5*	2011 carrying a mini-Tn5 inserted in *rpoN* Km^r	[91]
2011*mcherry*	2011 labeled with *mCherry* by a single chromosomal integration of pKOSm	This work
2011*egfp*	2011 labeled with *egpf* by a single chromosomal integration of pKOSe	This work
2011*ecpR1 egfp*	2011*ecpR1* labeled with *egpf* by a single chromosomal integration of pKOSe	This work
2011*relA*	*relA* deletion mutant	[38]
Sm2B6015	Rm2011 *visN* mutant	[42]
gcrA -eGFP	2011 carrying the fusion protein GcrA-EGFP	[46]
2011*gcrA*-P_{lac}*gcrA*	*gcrA* deletion mutant carrying P_{lac}*gcrA*	This work
2011*hfq*	*hfq* insertion mutant, Gm^r	[92]
Rm4011	2011 derivative strain, *expR*/*sinI* double mutant. Nx^r, Sm^r	[81]
4011*ecpR1*	*ecpR1* deletion mutant	This work
4011*ecpR1 rne675*	2011 carrying pK18mobII inserted in the 675th codon of *rne*, Km^r	[59]
S. medicae WSM419	$Acid^t$, sardinian isolate; Nx^r, Cm^r	[93]
S.fredii NGR234	Broad-host-range bacterium isolated from *Lablab purpureus*, Rf^r	[94]
A. tumefaciens C58	Wild-type, isolated from a cherry tree (*Prunus*) tumor Nx^r	[95]
A. radiobacter ATCC19358^T	*A. radiobacter* type strain, isolated from soil. Nx^r	[96]
R. etli CFN42^T	*R. etli* type strain, isolated from *Phaseolus* Nx^r, Sm^r	[97]
R. tropici CIAT899^T	*R. tropici* type strain, isolated from *P. vulgaris*. Sm^r	[98]
E. coli		
DH5α	F– *endA1 supE44 thi-1*l-*recA1 gyrA96 relA1 deoR*D*(lacZYA-argF)U169*	[99]
S17-1	*E. coli* 294 Thi RP4-2-Tc::Mu-Km::Tn7 integrated into the chromosome	[100]
Plasmids		
pPHUtrap	pPHU231 containing *sinI* 5´UTR fused to *egfp*, Tc^r	Matthew McIntosh
pP*ecpR1*_5'1-204	pPHUtrap with *ecpR1* promoter-*egfp* transcriptional fusion of the whole IGR	This work
pP*ecpR1*_5'1	pPHUtrap with *ecpR1* promoter-*egfp* transcriptional fusion until 1st 5'-end	This work
pP*ecpR1*_5'2	pPHUtrap with *ecpR1* promoter-*egfp* transcriptional fusion until 2nd 5'-end	This work
pP*ecpR1*_5'2-Pσ70	pTSS2-*egfp* carrying changes in the σ^{70}-dependent promoter -10 region	This work
pK18*mobsacB*	Suicide plasmid in *S. meliloti*, *sacB*, *oriV*, Km^r	[80]
pKdel*ecpR1*	pK18*mobsacB* with *ecpR1* flanking regions for geneSOEing	This work
pKPσ70*ecpR1*	pK18*mobsacB* with *ecpR1* locus region carrying changes in the σ^{70}-dependent promoter -10 region for double recombination	This work
pKdel*gcrA*	pK18*mobsacB* with *gcrA* flanking regions for geneSOEing	This work
pK18mobII	Suicide vector; mob lacZ Km^r	[101]
pKOSm	pK18mobII with P_{T5}:*mCherry* cassette fused to *recG*	Oliver Schauer
pKOSe	pK18mobII with P_{T5}:*egfp* cassette fused to *recG*	Pornsri Charoenpanich
pK18ins*gcrA*$_{300}$	pK18mobII carrying an internal fragment of *gcrA*, nt 4–297; Km^r	This work
pSRKKm	pBBR1MCS-2 derivative with a Plac promoter, *lacIq*, *lacZa*$^+$, Km^r	[102]
pRel$_{Sm}$	pSRKm carrying the Rel$_{Sm}$ coding sequence	[38]
pSRKKm *divK*	pSRKKm carrying the *divK* coding sequence	[32]
pSKControl$^+$	pSRKKm carrying the *smel812* coding sequence fused to *sinR*-P$_{sinI}$	This work
pSKEcpR1$^+$	pSRKKm carrying the *ecpR1* coding sequence starting from 5'1 fused to *sinR*-P*sinI*	This work
pSKEcpR1$_{5'2}^+$	pSRKKm carrying the *ecpR1* coding sequence starting from 5'2 fused to *sinR*-P*sinI*	This work
pSKEcpR1-1$^+$	pSKEcpR1$^+$ carrying 1 nt change in the first conserved loop	This work
pSKEcpR1-2$^+$	pSKEcpR1$^+$ carrying 2 nt changes in the first conserved loop	This work

pSKEcpR1-3^{+}	pSKEcpR1^{+} carrying 3 nt changes in the first conserved loop	This work
pSRKGm	pBBR1MCS-2 derivative with a Plac promoter, *lacIq*, *lacZa*+, Gm^{r}	[102]
P_{lac}*gcrA*	pSRKGm carrying the $gcrA_{Sm}$ coding sequence	This work
pSGControl^{+}	pSRKGm with *smel812* coding sequence fused to *sinR*-P_{sinI}	Jan Philip Schlüter
pSGEcpR1^{+}	pSRKGm with the *ecpR1* coding sequence fused to *sinR*-P_{sinI}	Lars-Ole Loehr
pWBT	pSRKGm carrying the T5 promoter sequence downstream the *lac* promoter	Matthew McIntosh
pWBT*pleD*	pWBT carrying the $pleD_{Sm}$ coding sequence	Simon Schäper
pR_EGFP	Reporter fusion plasmid for cloning of sRNA targets, Tc^{r}, Ap^{r}	[44]
p$gcrA_{-122+3}$-*egfp*	pR_EGFP expressing the *gcrA::egfp* translational fusion from TSS (-130)	This work
pP$gcrA_{-122+3}$-*egfp*	pR_EGFP expressing the *gcrA::egfp* translational fusion from its own promoter (-223, relative to the AUG)	This work
p$dnaA_{-17+30}$-*egfp*	pR_EGFP expressing the *dnaA::egfp* translational fusion from TSS1	This work
p$dnaA_{-56+30}$-*egfp*	pR_EGFP expressing the *dnaA::egfp* translational fusion from TSS2	This work
p$dnaA_{-70+30}$-*egfp*	pR_EGFP expressing the *dnaA::egfp* translational fusion from TSS3	This work
p$dnaA_{-154+30}$-*egfp*	pR_EGFP expressing the *dnaA*::*egfp* translational fusion from -154	This work
p$dnaA_{-198+30}$-*egfp*	pR_EGFP expressing the full *dnaA::egfp* translational fusion from putTSS4	This work
p$dnaA_{-198+162}$-*egfp*	pR_EGFP expressing the full *dnaA::egfp* translational fusion to +162	This work
p$ctrA_{-26+93}$-*egfp*	pR_EGFP expressing the *ctrA::egfp* translational fusion from TSS1	This work
p$ctrA_{-69+93}$-*egfp*	pR_EGFP expressing the *ctrA::egfp* translational fusion from TSS2	This work
p$minD_{-105+3}$-*egfp*	pR_EGFP expressing the *minD::egfp* translational fusion	This work
p$pleC_{-153+132}$-*egfp*	pR_EGFP expressing the *pleC::egfp* translational fusion	This work
p$ftsZ_{-99+108}$-*egfp*	pR_EGFP expressing the *ftsZ::egfp* translational fusion	This work
p$divJ_{-112+126}$-*egfp*	pR_EGFP expressing the *divJ::egfp* translational fusion	This work
pSMc00888$_{-235+57}$-*egfp*	pR_EGFP expressing the SMc00888*::egfp* translational fusion	This work
p$divK_{-85+45}$-*egfp*	pR_EGFP expressing the *divK::egfp* translational	This work
p*gcrA-BS-egfp*	$pgcrA_{-122+3}$-*egfp* carrying 2 nt compensatory changes in *ecpR1* interaction region	This work
p*gcrA-ATT-egfp*	$pgcrA_{-122+3}$-*egfp* carrying 3 nt changes in *ecpR1* interaction region	This work
p$dnaA_{-154+162}$-*egfp*	pR_EGFP expressing the *dnaA::egfp* translational fusion from -154 to +162	This work
p$dnaA_{-154+162}$-*BS5-egfp*	$pdnaA_{-154+162}$-*egfp* carrying 5 nt changes in *ecpR1* predicted binding site 5	This work
p$dnaA_{-56+30}$-*BS4-egfp*	p$dnaA_{-56+30}$-*egfp* carrying 5 nt changes in *ecpR1* binding site 4	This work
p$dnaA_{-56+30}$-*BS3egfp*	p$dnaA_{-56+30}$-*egfp* carrying 3 nt changes in *ecpR1* binding site 3	This work
p$dnaA_{-56+30}$-*BS3+4-egfp*	p$dnaA_{-56+30}$-*egfp* carrying 8 nt changes in *ecpR1* binding sites 3 and 4	This work
p$dnaA_{-154+162}$-*BS3+4-egfp*	p$dnaA_{-154+162}$-*egfp* carrying 8 nt changes in *ecpR1* binding sites 3 and 4	This work
p*dnaA-BSs-egfp*	$pdnaA_{-56+30}$-*egfp* carrying 3 nt compensatory changes in *ecpR1* binding sites 3 and 4	This work

Supporting references

90. Casse F, Boucher, C., Julliot, J.S., Michel, M., and Denarie, J. (1979) Identification and characterization of large plasmids in *Rhizobium meliloti* using agarose-gel electorphoresis. J Gen Microbiol 113: 229–242.
91. Pobigaylo N, Wetter D, Szymczak S, Schiller U, Kurtz S, et al. (2006) Construction of a large signature-tagged mini-Tn5 transposon library and its application to mutagenesis of *Sinorhizobium meliloti*. Appl Environ Microbiol 72: 4329-4337.
92. Voss B, Hölscher M, Baumgarth B, Kalbfleisch A, Kaya C, et al. (2009) Expression of small RNAs in Rhizobiales and protection of a small RNA and its degradation products by Hfq in *Sinorhizobium meliloti*. Biochem Biophys Res Commun 390: 331-336.
93. Howieson JGaE, M. A. (1986) Acid tolerance in the *Rhizobium meliloti–Medicago* symbiosis. Aust J Agric Res 37: 55–64.
94. Jarvis BD, Downer HL, Young JP (1992) Phylogeny of fast-growing soybean-nodulating rhizobia support synonymy of *Sinorhizobium* and *Rhizobium* and assignment to *Rhizobium fredii*. Int J Syst Bacteriol 42: 93-96.
95. Hamilton RH, Fall MZ (1971) The loss of tumor-initiating ability in *Agrobacterium tumefaciens* by incubation at high temperature. Experientia 27: 229-230.
96. Popoff MY, Kersters K, Kiredjian M, Miras I, Coynault C (1984) Taxonomic position of *Agrobacterium* strains of hospital origin. Ann Microbiol (Paris) 135A: 427-442.
97. Quinto C, De La Vega H, Flores M, Leemans J, Cevallos MA, et al. (1985) Nitrogenase reductase: A functional multigene family in *Rhizobium phaseoli*. Proc Natl Acad Sci U S A 82: 1170-1174.

98. Martinez-Romero E, Segovia L, Mercante FM, Franco AA, Graham P, et al. (1991) *Rhizobium tropici*, a novel species nodulating *Phaseolus vulgaris* L. beans and *Leucaena* sp. trees. Int J Syst Bacteriol 41: 417-426.
99. Grant SG, Jessee J, Bloom FR, Hanahan D (1990) Differential plasmid rescue from transgenic mouse DNAs into *Escherichia coli* methylation-restriction mutants. Proc Natl Acad Sci U S A 87: 4645-4649.
100. Simon R, Priefer U, Pühler A (1983) A Broad Host Range Mobilization System for *In Vivo* Genetic Engineering: Transposon Mutagenesis in Gram Negative Bacteria. Nature Biotechnology 1: 784-791.
101. Tauch A, Zheng Z, Pühler A, Kalinowski J (1998) *Corynebacterium striatum* chloramphenicol resistance transposon Tn5564: genetic organization and transposition in *Corynebacterium glutamicum*. Plasmid 40: 126-139.
102. Khan SR, Gaines J, Roop RM, Farrand SK (2008) Broad-host-range expression vectors with tightly regulated promoters and their use to examine the influence of TraR and TraM expression on Ti plasmid quorum sensing. Appl Environ Microbiol 74: 5053-5062.
