# Supplementary material for: A Stress-Induced Small RNA Modulates Alpha-Rhizobial Cell Cycle Progression
Source: PLoS Genet. 2015 Apr 29;11(4):e1005153. doi: 10.1371/journal.pgen.1005153 (PMC4414408; doi:10.1371/journal.pgen.1005153)
Supplement: S12 Table — (PDF) [file pgen.1005153.s012.pdf]

S12 Table. Oligonucleotides used in this study.

Primer name	Sequence (5´ -> 3´)	Use in this study
Smc291NP_F	CAGGCAATGCTGATAAGCGAGG	*ecpR1* Northern probe
Smc291NP_R	TTCCGGCGCTTGAACCTGAA	
5SRNA_F	TGGTTCTGGCGGGGTGGCTGC	5S Northern probe
5SRNA_R	TACTCTCCCGCGTCTTAAGACGAA	
Xp291-204 _F1	ccac AAGCTT CCGCCGCACATGCTGAGCTT	Construction of *epcR1* promoter-*egfp* transcriptional fusions
Xp291_F2	cgct AAGCTT CGACACTGTCCGATTCTGAA	
HP291-5'-1_R1	cgca TCTAGA TTCCTTCCTCGCTTATCAGC	
Hp291- 5'-2 _R2	cgcg TCTAGA ATCATGGGGTAACCGAATGCT	
P291KO70_F	TTTTTAGTTTCAGGACCGATTGATAAGCGAGGAAGGAAAC	
P291KO70_R	ATCGGTCCTGAAACTAAAAAATCAAT	
1Smc291KOSmaI_F2	ccag CCCGGG ACGGACTACATCATGCGACC	Construction of pKdel*ecpR1*
2FusSmelC291KO_R	CTCGCTTATCAGCATTGCCTG	
3FusSmC291KO_F	CAGGCAATGCTGATAAGCGAGCTTCCGTTCGTTTCAGGTTC	
4Smc291KOHind_R2	gcgt AAGCTT CAGCGAATCGCAGGCAGCG	
291pKO70SacI_F	TTTTTAGTTTCAGGGAGCTCTGATAAGCGAGGAAGGAAAC	Construction of pKPσ70*ecpR1*
291pKO70SacI_R	GAGCTCCCTGAAACTAAAAAAAAATCAAT	
259up291*pleD*_F	GCTCTATCAGGCGAAGAACG	Confirming *ecpR1* mutation
271down291*rpmg*_R	GCTACCACCATCAAGATCAAGC	
1*gcrA*KOHind_F	ccag AAGCTT TGACGTGGAACTTCTCGACGTG	Construction of pKdel*gcrA*
2Fus*gcrA*KO_R	GGAATCCGTCACCGCTCTCCG	
3Fus*gcrA*KO_F	CGGAGAGCGGTGACGGATTCCGCCTACCAGCCCTCTGCCGA	
4*gcrA*KOBamHI_R	gcgt GGATCC GAAGATTGCCGTAGTCGAAGA	
200up*gcrA*_F	TGCAACGCTCCGACAGCAAGT	Confirming *gcrA* chromosome mutation
799down*gcrA*_R	CATGACGGCTGTCGTGCGTAGA	
Hpk18insgcrAF	ccag AAGCT TAACTGAAGAAGCTGTGGTCGGA	Construction of pk18ins*gcrA*$_{300}$
EcopkinsgcrA300R	atat GAATT CCAGGTCGACTGCGAGCTCTTC	
*sinR*_NdeIF	gcca CATATG GCTAATCAACAGGCTGTC	Construction of the *sinR*-P*sinI* fusion system for overexpression
TSS3_28bp_b_*sinIR*	GTAGCGATGCTGTCAGGCTC	
SmelC812_fwd	GAGCCTGACAGCATCGCTACATCCTTGTCGTCCCTGCCTTGCATAC	Construction of pSKControl^{+} and pSGControl^{+}
SmelC812_XbaI_rev	tctg TCTAGA GAGAAGTCGTCCGCTTTCTCTG	
SmelC291_ fwd	GAGCCTGACAGCATCGCTACGCGAGGAAGGAAACTTCCACAG	Construction of pSKEcpR1^{+} and pSGEcpR1^{+}
SmelC291_ XbaI_rev	tctg TCTAGA TTCCGGCGCTTGAACCTGAAAC	
SmelC291TSS2_F	GAGCCTGACAGCATCGCTACTTACCCCATGATGCTCAGGTC	Construction of pSKEcpR1$_{5'2+}$
C291IRKO1_F	CATGATGCTCAGGTCCCCCGCATCTCCTGGGTCGTGG	Construction of pSKEcpR1-1^{+}
C291IRKO1_R	GGGACCTGAGCATCATGGGGTA	
C291IRKO2_F	CATGATGCTCAGGTCCCCGGC ATCTCCTGGGTCGTGGGGT	Construction of pSKEcpR1-2^{+}
C291IRKO2_R	CGGGGACCTGAGCATCATGGGGTA	
C291IRKO3_F	CATGATGCTCAGGTCCTAAGCATCTCCTGGGTCGTGGGGTC	Construction of pSKEcpR1-3^{+}
C291IRKO3_R	TTAGGACCTGAGCATCATGGGGTAA	
*gcrA*OEI_NdeI_F	gcca CATATG AACTGGACTGACGAGCGG	Construction of P$_{lac}$*gcrA*
*gcrA*OEI_BamHI _R2	tctc GGATCC CGTCGTGCATCCAGAGCGGAATGAG	
BHI5UTR*gcrA*_F2	cggc GGATCC CATTCAATTACTAGACGCATG	Construction of the 5´UTR-*egfp* fusions
Hp*gcrA*+5UTR_F	tcgc AAGCTT ATGCAACGCTCCGACAGCAAGT	
5UTR*gcrA*Nh_R2	acta GCTAGC CATCTCTGCACTCCCCGTTCTCG	
5UTR*dnaA*-198_F	atct GGATCC GCGAGGGGACATTTGCTTGAGG	
5UTR*dnaA*-154_F	tata GGATCC GCGTGCCGTGGCGGGATATG	
5UTR*dnaA*-70_F	tcta GGATCC GCGGGCGGGCATAAAAGATGAG	
5UTR*dnaA*-56_F	caga GGATCC AAGATGAGGCCGCCAGAAATGAC	
5UTR*dnaA*-17_F	caga GGATCC AATTGGAAGGCGGCAAGATG	
5UTR*dnaA*+162_R2	cacg GCTAGC AAACCAACTTGCGAAGACATC	
5UTR*dnaA*+30_R6	atta GCTAGC ACCTCCAGGTGCCGTCGCCA	
BglII5UTR*ctrA*-26_F	atta AGATCT CTTGGGCGGCGGAAAAGGG	
BglII5UTR*ctrA*-69_F	atta AGATCT AGGCAACGACTGGATCCGTA	
5UTR*ctrA*Nh+93_R2	ccga GCTAGC ATCTGTGGTGTAGACGTTGA	
5UTR*minD*-105_F	actt GGATCC TTAACTGAGCCGGTTGCCGG	
5UTR*minD*+5_R	ctac GCTAGC CATCTTCCCGCTCCTGTTG	
BHI5UTR-170*pleC*_F	gact GGATCC CCTGTGATTCATTGAGTCAG	
Nh5UTR*pleC*+135_R	tgca GCTAGC CATTGCGAGCTTCTGCGACGC	
BamHI*ftsZ1*-99F	gac GGATCC TTCTAGATCGGTCGGCATATC	
NheI*ftsZ1*+108R	gtag GCTAGC GGCGGTGATCATGTTGTTGACG	
5UTR*divJ*-112_F	gatt GGATCC TGCGAGATTCGGGTCATCACG	
5UTR*divJ*+126_R2	aact GCTAGC CAAGGATACGGCAGCGACGGC	
5UTR00888-235_F	cggc GGATCC ACAGCAACCTGAATGATCGTCGG	
5UTR00888+57_R	atta GCTAGC GCTTGCCTCCAGAATGATCCT	
5UTR*divK*-96_F	atgc GGATCC AGCCTGGCGATCGACGAAATG	

5UTR*divk*+45_R2	gccaGCTAGC CATATTCAGCTCGTTATCCTC	
*gcrA*BSKOA_ F2	AATTACTAGACGCATGCTAAGGACCTAGTCACCAAAAGTATTAT	
*gcrA*BSKOA_R	TTAGCATGCGTCTAGTAATTGAATG	
gcrAIRKOTTA_ F2	AATTACTAGACGCATGCTTAGGACCTAGTCACCAAAAGTATTAT	
gcrAIRKOTTA_ R	TTAGCATGCGTCTAGTAATTGAATG	
*dnaA*BS5KOB_ F	AGCAATCAGTCTCAGGAGTCTGGGGAAAAACACGACATGCGGCA	
*dnaA*BS5KOB_R	AGACTCCTGAGACTGATTGCTTCCG	
*dnaA*BS4KOE_ F	TGGAAGGCGGCAGAATGATTATGAATTTGGCGACGGCACC	
*dnaA*BS4KOE_R	ATCATTCTGCCGCCTTCCAATTTCATTATTCCGATGCCG	
*dnaA*BS3KOA_ F	ATAATGAAATTGGAAATCAGCAAGATGCGGATGAATTTGGC	
*dnaA*BS3KOA_R	GCTGATTTCCAATTTCATTATTCCGAT	
dnaABS3_4KOE_ F	TTGGAAATCAGCAGAATGATTATGAATTTGGCGACGGCACC	
dnaABS3_4KOE_R	AATCATTCTGCTGATTTCCAATTTCATTATTCCGATGCCG	
*dnaA*BSKO1-2_ F	ATAATGAAATTGGAAGGCCGGAAGATGCCGATGAATTTGGC	
*dnaA*BSKO1-2_R	GGCATCTTCCGGCCTTCCAATTTCATTATTCCGAT	
PCR 1	CGGGCCTCTTCGCTATT	
PCR 2	TTAGCTCACTCATTAGG	Plasmids for sequencing
Egfp-139_rev	GATGAACTTCAGGGTCAGCTTG	
RT*ecpR1*+30_F	TTACCCCATGATGCTCAGGTC	
RT*ecpR1*+131_R	GTCGACTGGTCCGGTTGCAG	
RT5UTR*gcrA*-122_F	AATTACTAGACGCATGCGGC	
RT5UTR*gcrA*+20_R	CGCTCGTCAGTCCAGTTCAT	
RT*gcrA*+209_F	ACCGAACTACGCTGCGCGTT	
RT*gcrA*+325_R	TGTCGATCTGCAGCTCCTGGT	
RT5UTR*dnaA*1-432_F	ATTCCACGTCGATCAATCAA	
RT5UTR*dnaA*1-318_R	GAGCCTCCTTCTTCCTGCTCCA	
RT5UTR*dnaA*2-248_F	ACGGTTCCGGTCGGCTTGAA	
RT5UTR*dnaA*2-154_R	CTGCGCTCATTCGCTTGCCT	
RT*dnaA*+263_F	TGATCACCACGCTCGTCCAG	
RT*dnaA*+374_R	CGGCAACGCTCTCTTCAGGA	
RT*ctra*+308_F	CCACAAGGACGAGCTGGTGG	
RT*ctrA*+400_R	GGTCTTGGCGTCCAGGTTGA	
RT *divJ*+388_F	GCGATGCGAGTGAGTATCTGA	qRT-PCR studies
RT *divJ*+511_R	GATGTCCACGGCAGAACGCT	
RT5UTR00888-235_F	ACAGCAACCTGAATGATCGTCGG	
RT5UTR00888+57_R	GCTTGCCTCCAGAATGATCCT	
RTSMc00888+493_F	TCTGCGTCAAGGACCGTAACC	
RTSMc00888+654_R	CTCCAGGACGTGGCGATCCGA	
RT*ftsZ*_F	AACCAGAACCTCTTCCGCATCGC	
RT*ftsZ*_R	TCCTTGACCATGAGGTCGGT	
RT*pleC*_F	TCTCATCGTGGCGATCTCACG	
RT*pleC*_R	CCTCGGCGAAGAGTTCCGATC	
RT*minD*_F	CCTCGCCGTCATCGTCACCAAT	
RT*minD*_R	TCAGCAGCAGGTGCTTCTCGA	
RTSMc01852+244_F	TCACCAACACTGCCGACTGC	
SMc01852+355down	TCGTGTGCAGGATGCTGATG	

Small case indicates linker regions and restriction site sequences are underlined.
