# Supplementary figures and images for: A Stress-Induced Small RNA Modulates Alpha-Rhizobial Cell Cycle Progression
Source: PLoS Genet. 2015 Apr 29;11(4):e1005153. doi: 10.1371/journal.pgen.1005153 (PMC4414408; doi:10.1371/journal.pgen.1005153)

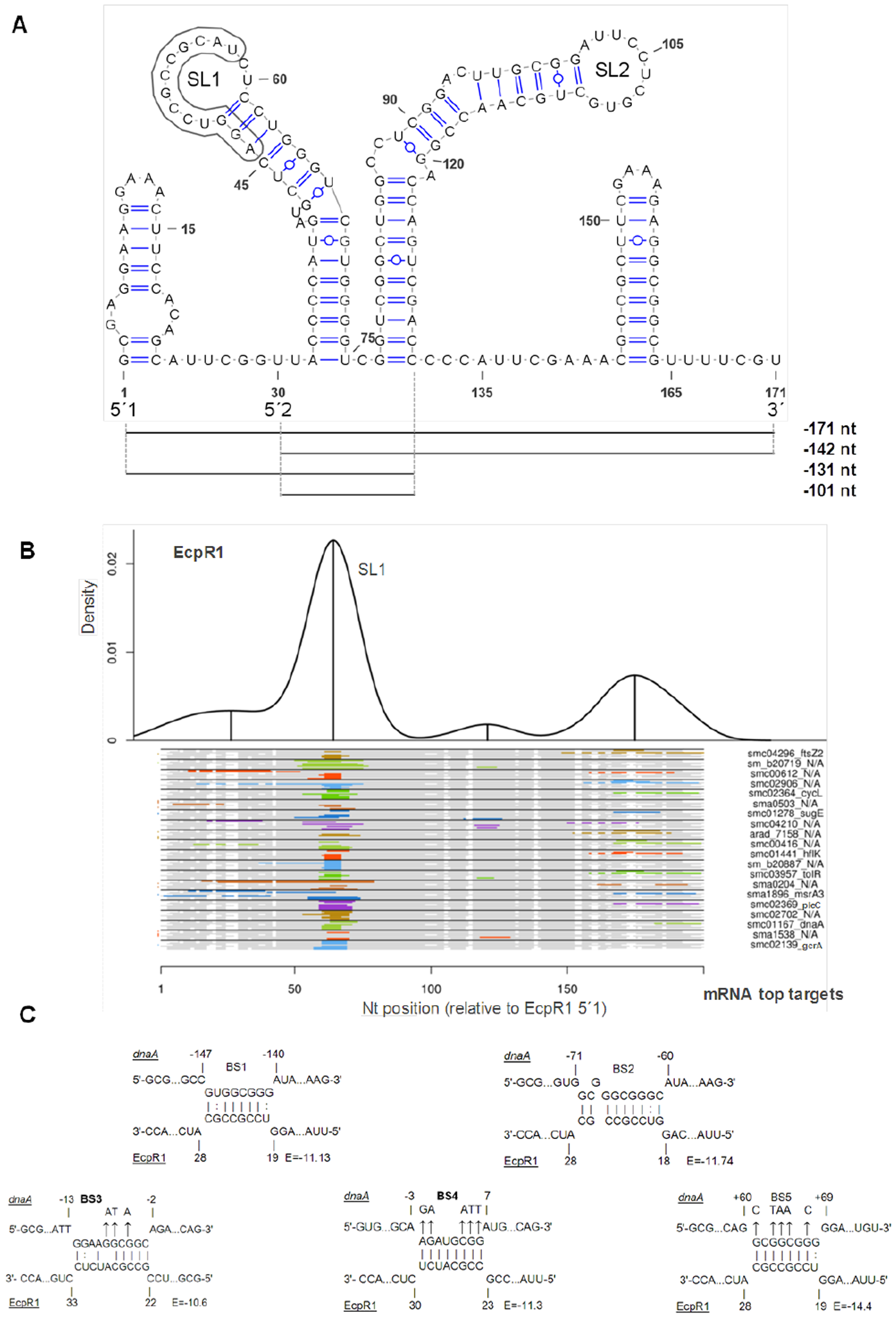

Supplement: S1 Fig — (A) Secondary structure of the EcpR1 171 nt full length variant with a minimum free energy of -82.10 kcal/mol. Nucleotide positions relative to the first 5’-end are shown. SL, stem loop domain. The 13 nt region predicted to bind the gcrA mRNA is boxed. Estimated 5’- and 3’-ends of the four different EcpR1 variants are mapped below. (B) Visualization of the predicted interaction domains in the EcpR1 full length sequence. The density plot shows the relative frequency of a specific EcpR1 nucleotide position participating in the top predicted target interactions (P≤0.002). The alignments are shown for the top 20 targets in the EcpR1 prediction (S. meliloti and seven closely related Rhizobiaceae). The schematic alignment of homologous sRNAs and targets shows the predicted interaction domains: aligned regions are displayed in grey, gaps in white, and predicted interaction regions in different colors. The S. meliloti locus tag and gene name (N/A, not available) of the predicted targets are given on the right. (C) Predicted EcpR1 binding sites BS1 to BS5 of the dnaA mRNA. Nucleotide exchanges in the predicted binding sites BS3 to BS5 that were carried out in different dnaA reporter constructs are indicated by arrows and confirmed interactions are shown in bold (see results in Fig 5F). The predicted energy score (E) is indicated in kcal/mol. (TIF) [file pgen.1005153.s013.tif]

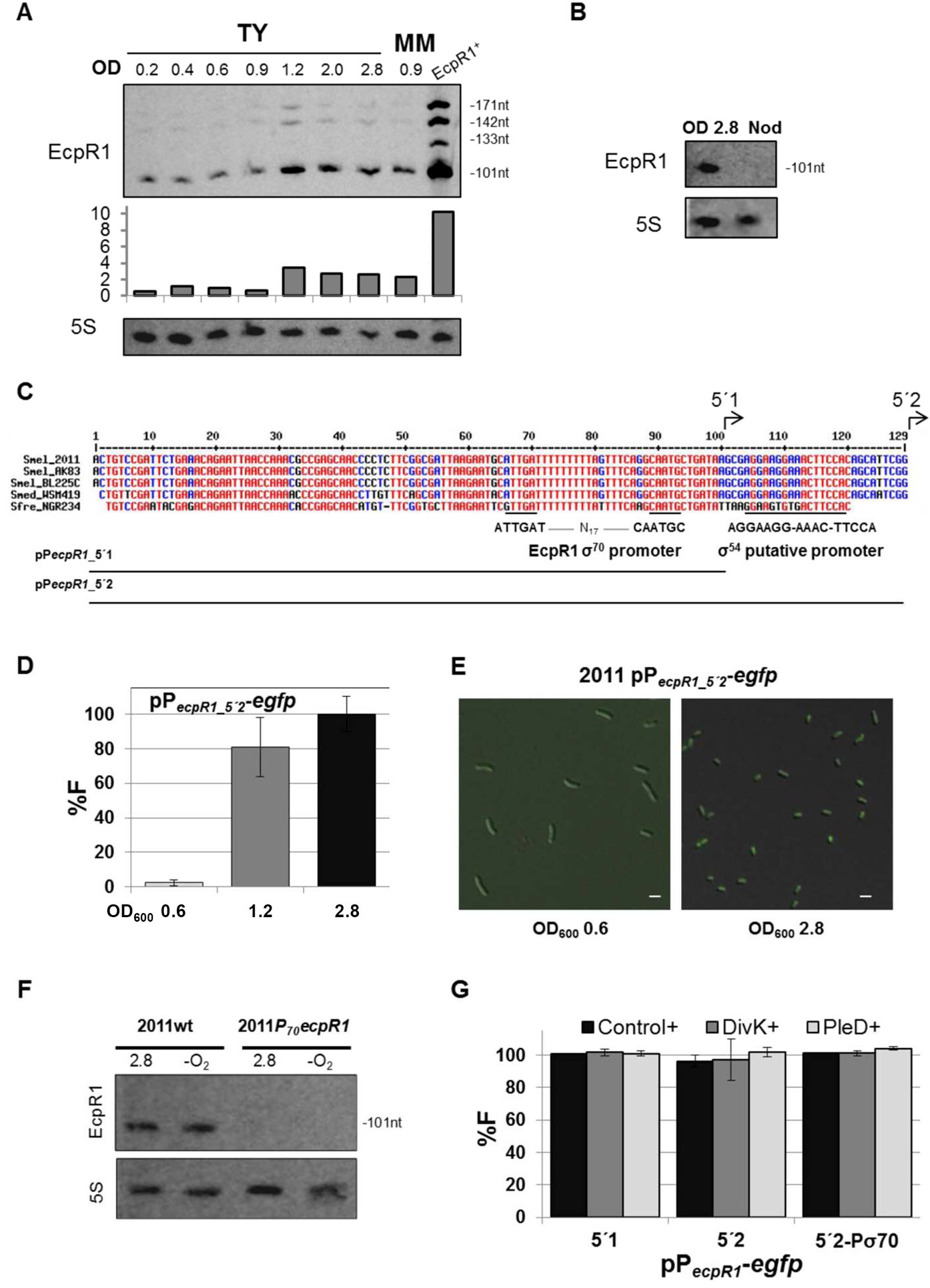

Supplement: S2 Fig — Northern blot detection of the EcpR1 transcript in Rm2011 wild type at different cell densities (OD600) in TY medium and minimal medium (MM) or in Rm4011 strain carrying pSKEcpR1+ 4 hours after induction with IPTG (EcpR1+) (A), in TY medium and 28 days-old mature symbiotic nodules (B) and in the 2011Pσ70 ecpR1 strain carrying a mutation in the -10 region of the σ70-type promoter in stationary growing and oxygen depleted bacteria in TY medium (F). Plots underneath the Northern blot in (A) represent hybridization signal intensities relative to the level of the EcpR1 101 nt variant in Rm2011 growing in TY rich medium at OD600 of 0.6, which has been normalized to 1. Promoter alignment of the EcpR1–100 region in different Sinorhizobium strains (C). RNAseq-detected EcpR1 5’-ends in Rm2011 are depicted by arrows and the predicted σ70- and σ54-dependent promoters are underlined. Nucleotide positions are numbered relative to the 5′1 end. Highly and weakly conserved nucleotides are represented as red or blue letters, respectively. Promoter consensus sequences derived from S. meliloti 1021 and fragments included in the ecpR1 transcriptional fusions are indicated below. Means of relative fluorescence intensity values at different cell densities of Rm2011/pPecpR1_5’2 grown in TY (D) and of Rm2011 harbouring the corresponding pPecpR1 and the empty vector control, pleD or divK overexpression plasmids grown in TY medium supplemented with IPTG (G). The standard deviation represents at least three independent measurements of three double transconjugants grown in six independent cultures. Specific activities were normalized to the levels of the stationary phase cultures (OD600 of 2.8) (D) or to the cultures lacking IPTG (G) to yield percent relative fluorescence (%F). (E) Fluorescence microscopy of exponential and stationary phase Rm2011 cells carrying pPecpR1_5’-2 in TY medium. Bars denote 2 μm. (TIF) [file pgen.1005153.s014.tif]

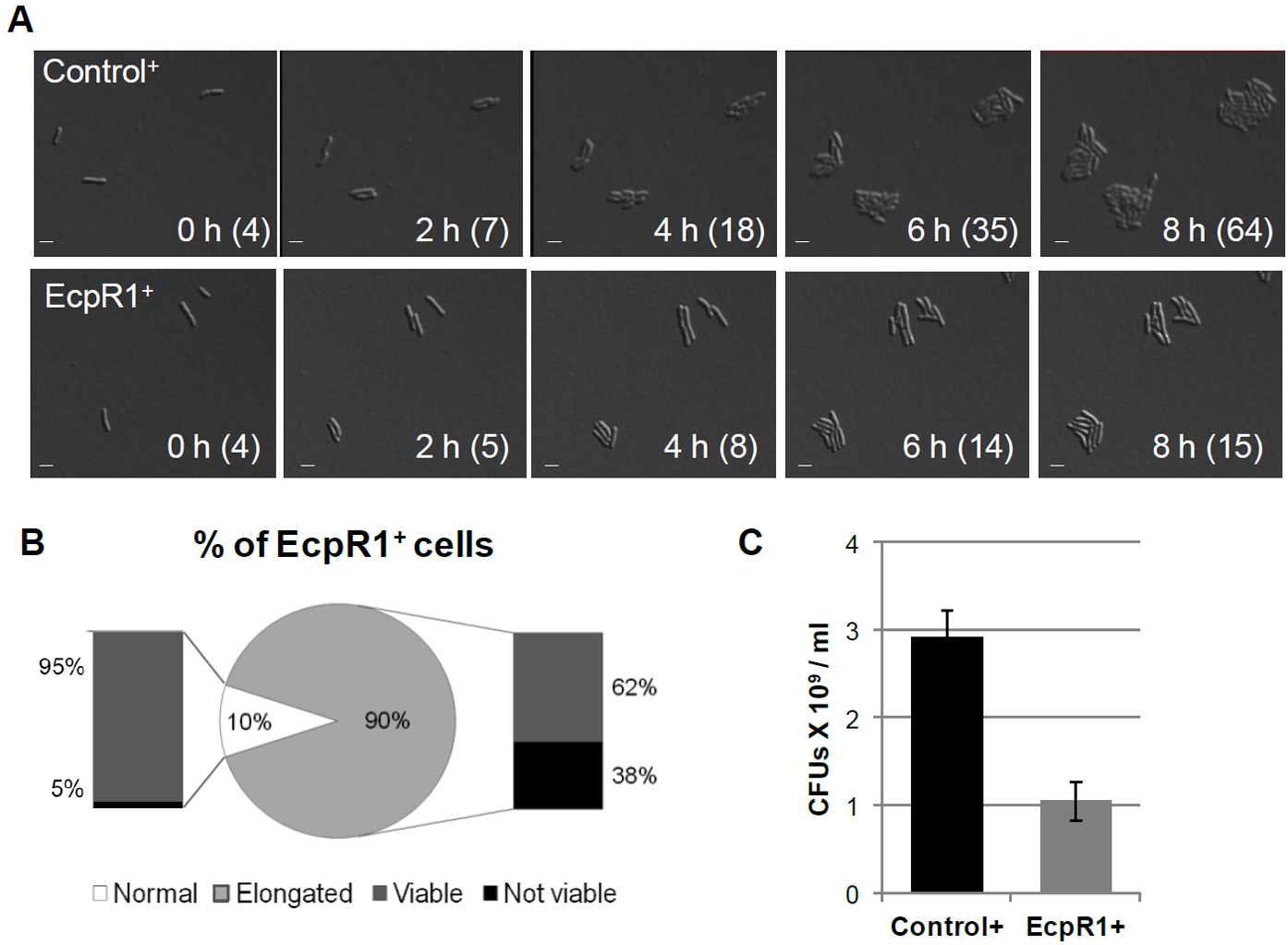

Supplement: S3 Fig — (A) Time lapse microscopy of Rm4011 cells overexpressing either the control RNA gene SmelC812 (Control+) or ecpR1 (EcpR1+) after addition of IPTG. Bars denote 2 μm. Cell numbers are indicated in brackets. Doubling times of ~2 hours and ~4 hours were determined for the control RNA gene and ecpR1 overexpressing strains, respectively. (B) Abundance of normal-sized and elongated cells in EcpR1+ cultures treated with IPTG for 30 hours and proportion of stationary cells that resumed growth after washing of cells and transfer to fresh medium lacking the inductor. Proportions were determined by time-lapse microscopy (n = 500). (C) Colony forming units (CFUs) of indicated strains after 3 cycles of re-growing on TY medium supplemented with IPTG for 48 h. (TIF) [file pgen.1005153.s015.tif]

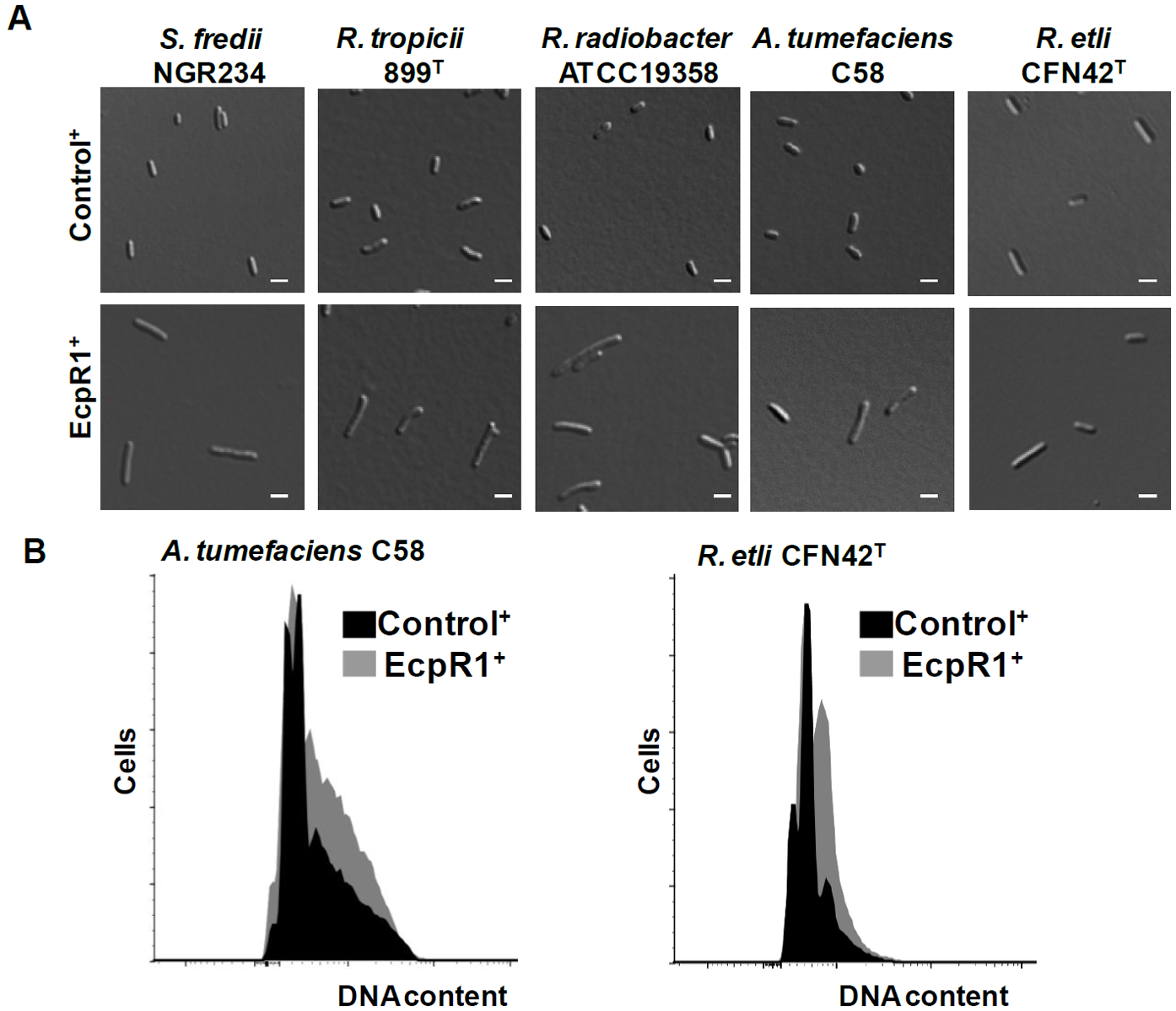

Supplement: S4 Fig — Cell morphology (A) and DNA content (B) of different species overproducing either the control RNA SmelC812 (Control+) or EcpR1 (EcpR1+) 20 hours after addition of IPTG. Bars denote 2 μm. (TIF) [file pgen.1005153.s016.tif]

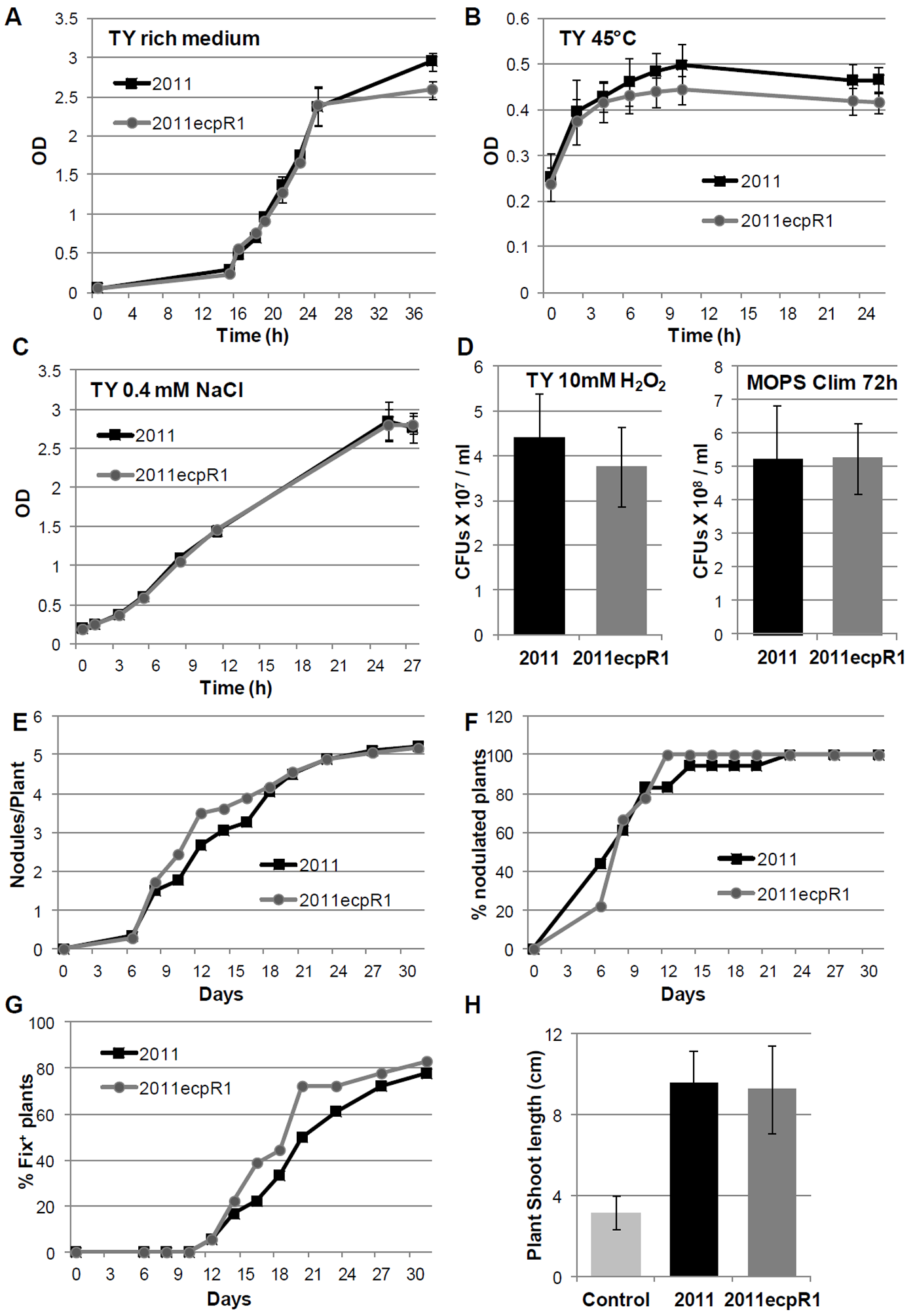

Supplement: S5 Fig — Growth rates of Rm2011 and 2011ecpR1 were compared in TY rich medium at 30°C (A), 45°C (B) or after adding 0.4 mM of NaCl (C). Cell viability (CFU/ml) of these two strains was compared after adding 10 mM H2O2 to logarithmic cultures in TY for 30 minutes or after growing in defined carbon-limited minimal medium (mannitol 2 g l-1) for 72 h (D). Error bars indicate the standard deviation of at least two replicates. Symbiotic phenotype of M. truncatula inoculated with 2011 wild type or 2011ecpR1. Time course of S. meliloti-induced nodule production (E). Percentage of plants developing nodules (F), and showing a Fix+ phenotype (G). Shoot length of plants growing in the absence of nitrogen 30 days after inoculation with S. meliloti 2011, 2011ecpR1, and uninoculated (control) (H). Error bars indicate the standard error. All samples were collected from the same experiment (20 plants). Nodulation assays were repeated three times with similar results. (TIF) [file pgen.1005153.s017.tif]

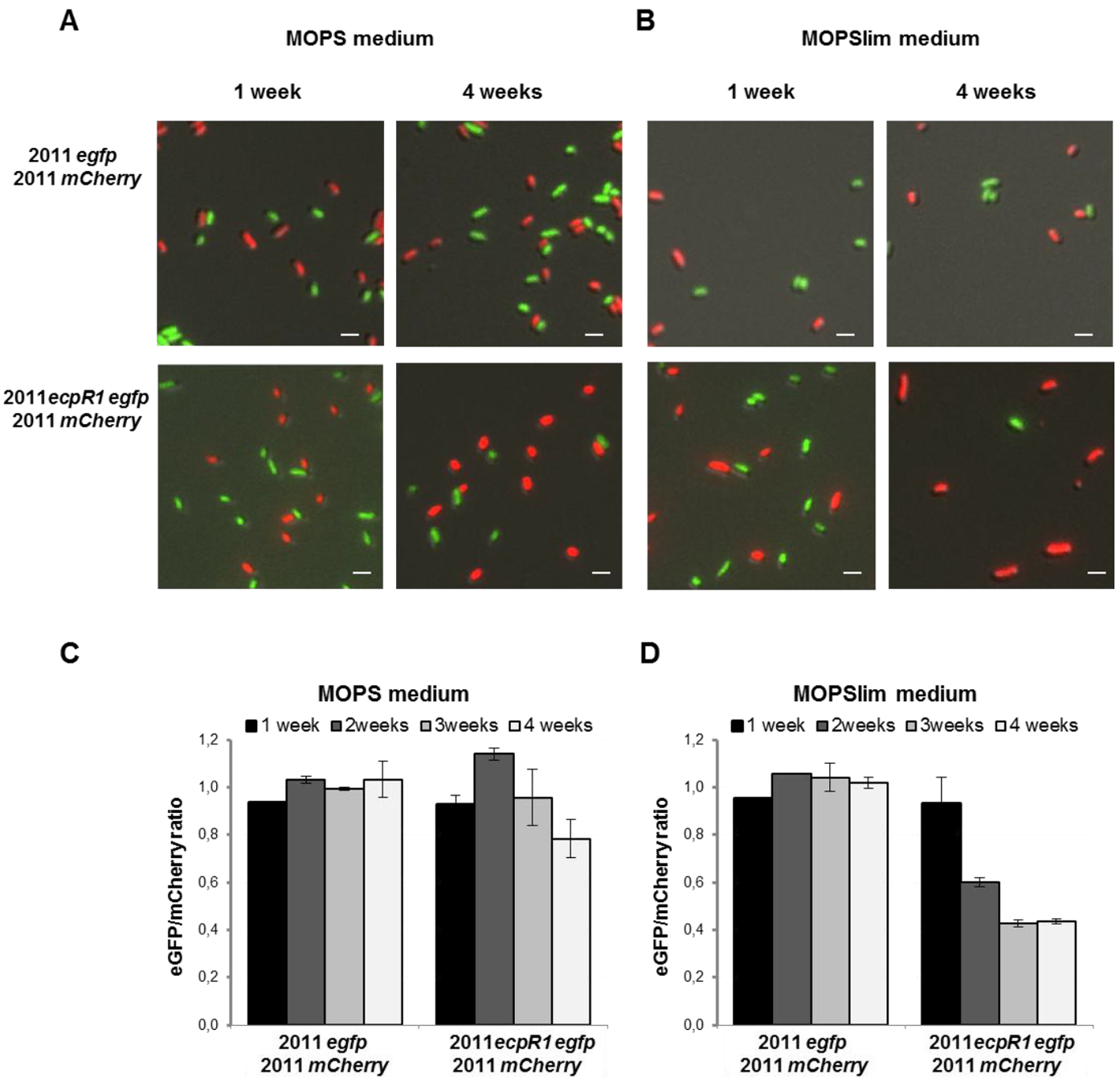

Supplement: S6 Fig — Representative fluorescence microscopy images (A-B) and means of eGFP:mCherry fluorescence ratios (C-D) of 2011 mCherry mixed with either 2011egfp or 2011ecpR1 egfp cell cultures at a 1:1 ratio in MOPS (A, C) or MOPSlim media (B, D) at the indicated time points. Every 7 days the mixed population was diluted 1000-fold in fresh media. Standard deviation represents three determinations of three independent cultures. Bars denote 2 μm. (TIF) [file pgen.1005153.s018.tif]

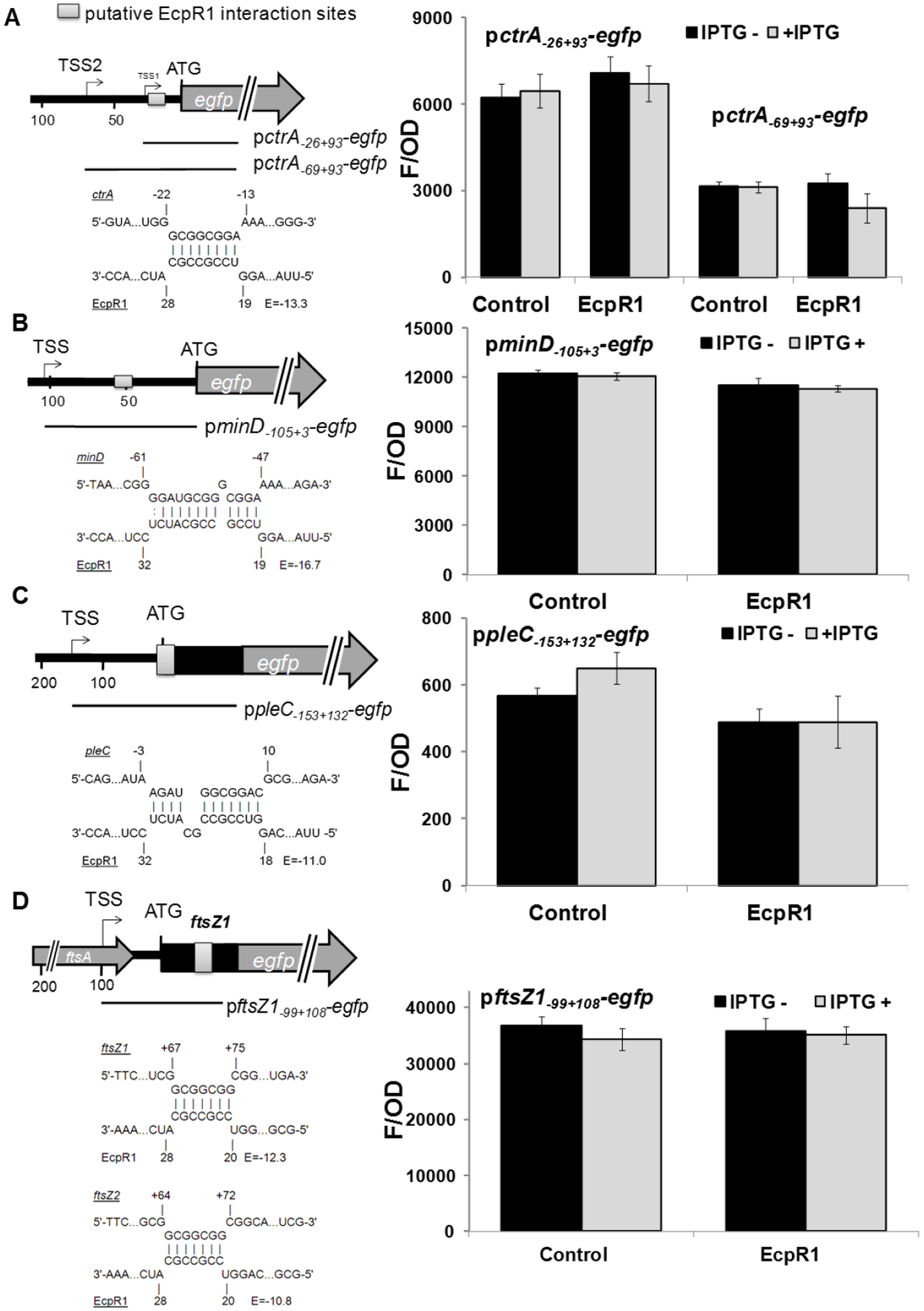

Supplement: S7 Fig — Predicted thermodynamically favored antisense interaction regions in ctrA (A), minD (B), pleC (C) and ftsZ1 (D) mRNAs, schematic representations of translation fusions to egfp, and fluorescence measurements mediated by these constructs in Rm4011ecpR1 carrying pSKEcpR1+ or pSKControl+. Numbers denote positions relative to the AUG start codon of the mRNA and the second 5’-end of EcpR1. The predicted energy score (E) is indicated in kcal/mol. The standard deviation represents at least three independent determinations of three double transconjugants grown in six independent cultures. (TIF) [file pgen.1005153.s019.tif]

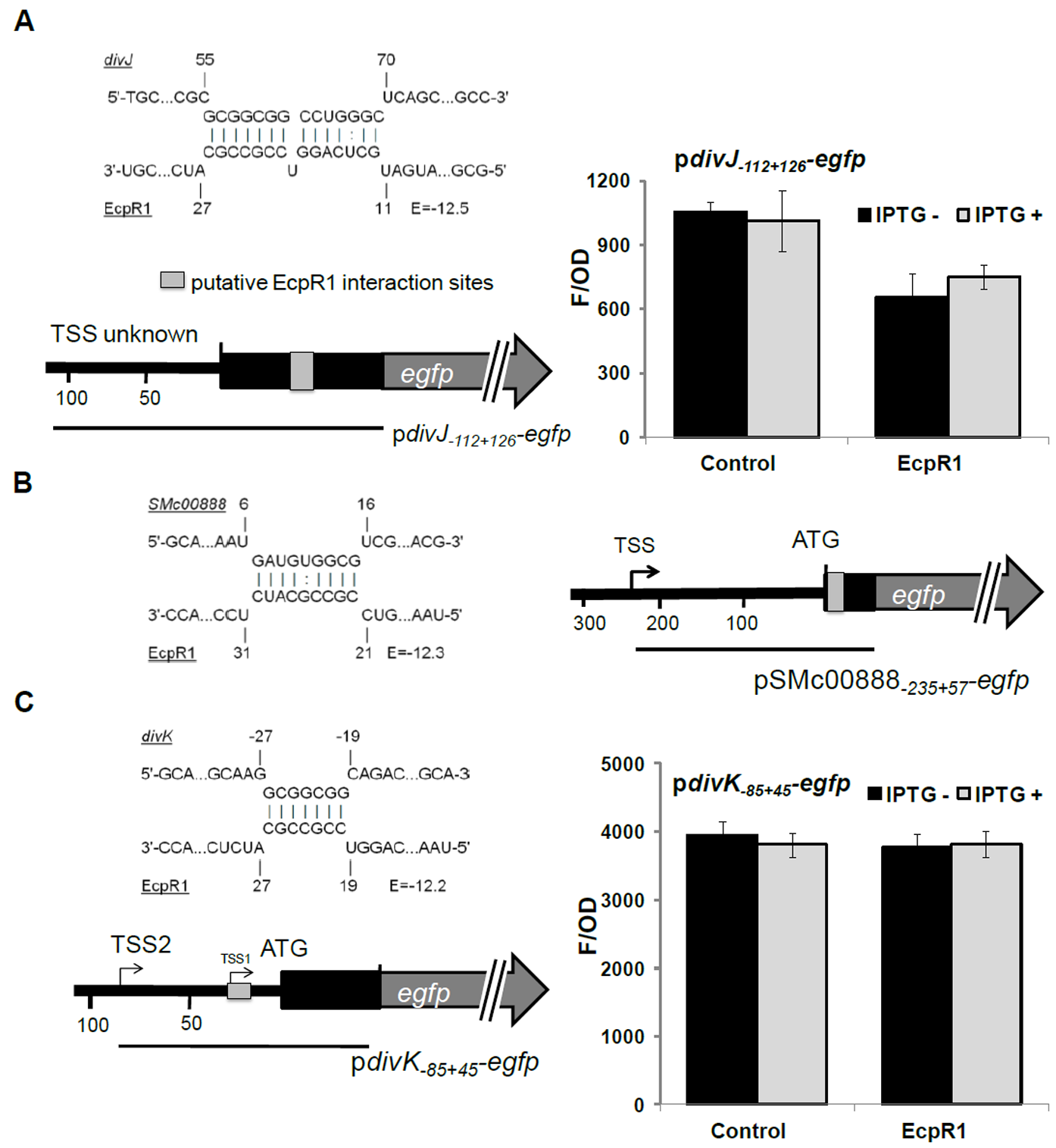

Supplement: S8 Fig — Predicted thermodynamically favored antisense interaction regions in divJ (A), SMc00888 (B), and divK (C) mRNAs, schematic representations of translation fusions to egfp, and fluorescence measurements mediated by these constructs in Rm4011ecpR1 carrying pSKEcpR1+ or pSKControl+. Numbers denote positions relative to the AUG start codon of the mRNA and the second 5’-end of EcpR1. The predicted energy score (E) is indicated in kcal/mol. The standard deviation represents at least three independent determinations of three double transconjugants grown in six independent cultures. (TIF) [file pgen.1005153.s020.tif]

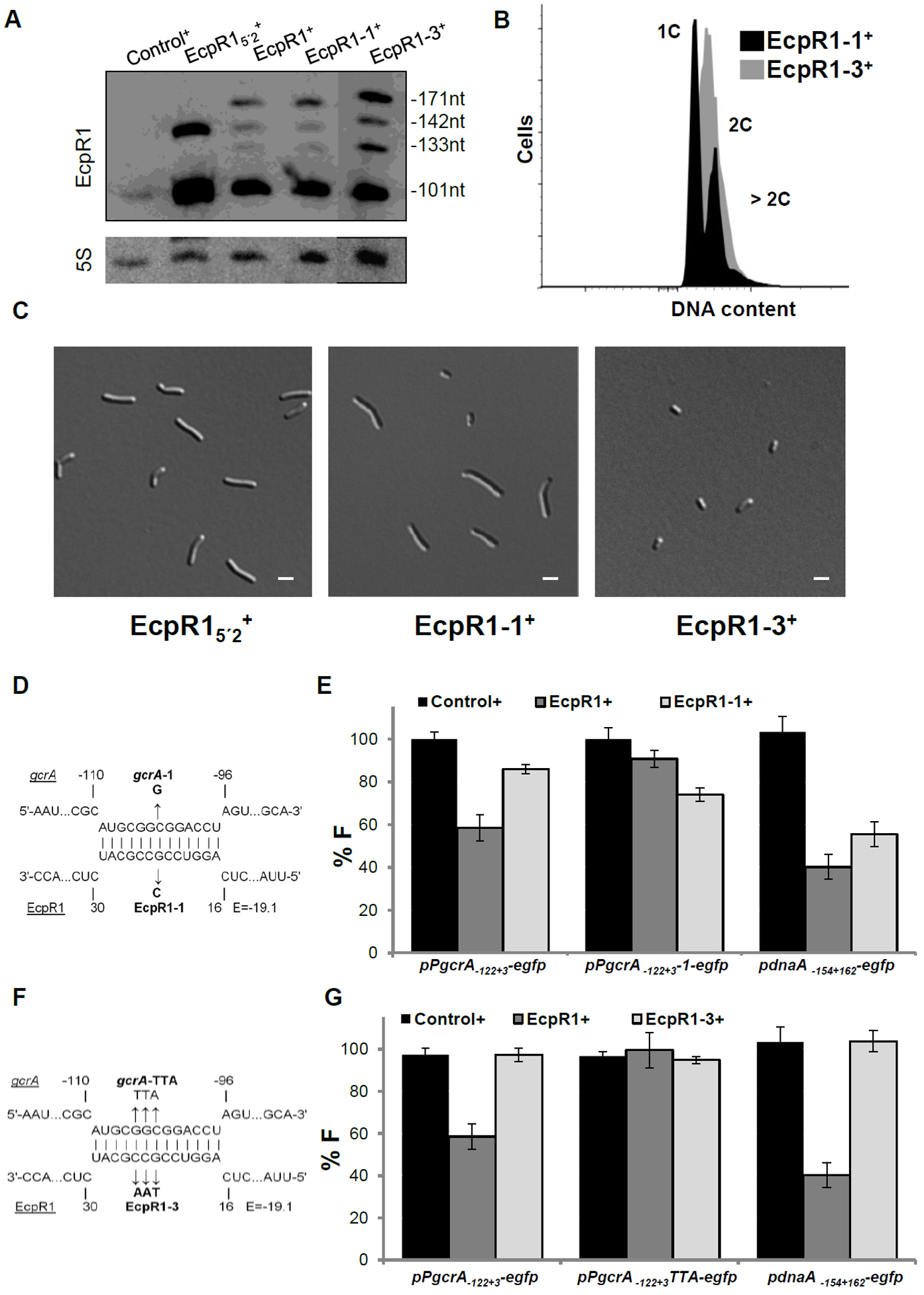

Supplement: S9 Fig — Northern blot detection (A), DNA content (B) and morphological phenotype (C) of Rm4011ecpR1 overexpressing ecpR1 5’2, ecpR1-1 and ecpR1-3, or control SmelC812. The bar represents 2 μm. (D, F) Predicted duplexes between EcpR1 and the gcrA mRNA. Nucleotide exchanges in EcpR1 and the gcrA mutant variants are denoted in bold. (E, G) Fluorescence measurements of 4011ecpR1 co-transformed with ecpR1, ecpR1-1, ecpR1-3, or control SmelC812 overexpression plasmids and the indicated reporter plasmids. The standard deviation represents at least three independent determinations of three double transconjugants grown in six independent cultures. (TIF) [file pgen.1005153.s021.tif]
